# From Molecular to Polymeric Donors: Prolonged Charge Separation in Modular Photoredox-Active Ru(II) Polypyridyl-Type Triads

**DOI:** 10.1021/acs.inorgchem.4c03693

**Published:** 2024-11-25

**Authors:** Alexander Kleine, Charlotte Mankel, Andrea Hainthaler, Maria Wächtler, Benjamin Dietzek-Ivanšić, Ulrich S. Schubert, Michael Jäger

**Affiliations:** † Laboratory of Organic and Macromolecular Chemistry (IOMC), Friedrich Schiller University Jena, Humboldtstr. 10, 07743 Jena, Germany; ‡ Institute for Physical Chemistry (IPC), Friedrich Schiller University Jena, Helmholtzweg 4, 07743 Jena, Germany; § Center for Energy and Environmental Chemistry Jena (CEEC Jena), Friedrich Schiller University Jena, Philosophenweg 7a, 07743 Jena, Germany; ∥ Research Department Functional Interfaces, Leibniz Institute of Photonic Technology Jena, Albert-Einstein-Straße 9, 07745 Jena, Ger-many; ⊥ Chemistry Department and State Research Center OPTIMAS, RPTU Kaiserslautern-Landau, Erwin-Schrödinger-Straße 46, 67663 Kai-serslautern, Germany

## Abstract

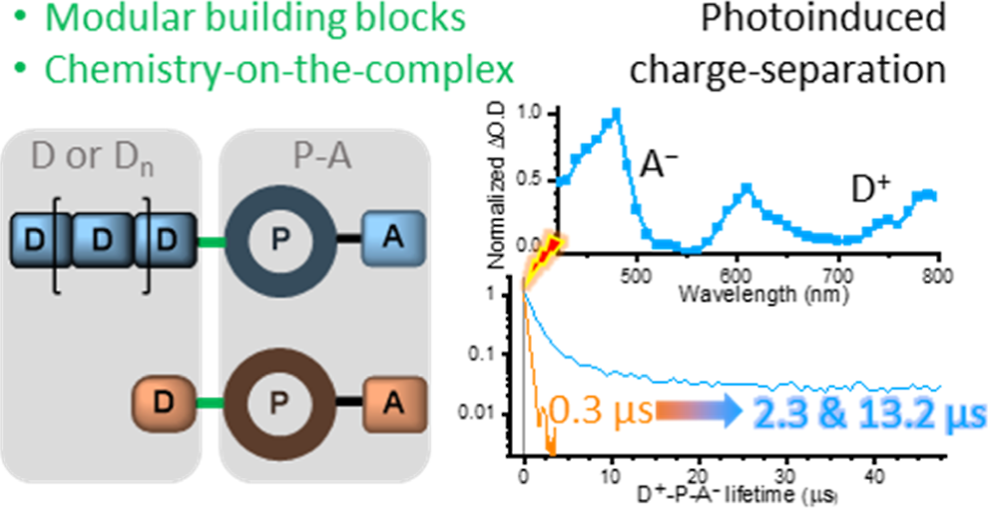

In this contribution, the divergent modular synthesis of photoredox-active dyads, triads and a tetrad descending from one ligand precursor is presented by combining “chemistry-on-the-ligand”, stepwise complexation and “chemistry-on-the-complex” with minimal synthetic efforts. In the final step, Pd-mediated borylation and subsequent Suzuki–Miyaura cross-coupling was employed to introduce the different (multi)donor moieties at the preassembled P–A dyad subunit. The (spectro-)electrochemical data revealed preserved redox properties of the subunits and minimal driving force for oxidative quenching by the naphthalene diimide-based (NDI) acceptor and, thus, high-energy charge separated (CS) states. Time-resolved transient absorption and emission data revealed the formation of long-lived CS states in the polymer-based triads, i.e., the CS lifetime is extended by 2 orders of magnitude in comparison to the molecular triad. The long-lived CS state (13.2 μs) of the conjugated polycarbazole (Carb_*n*_) multidonor demonstrates that the rational modular design and efficient synthesis of advanced photoredox-active assemblies can be readily achieved by late-stage diversification utilizing the “chemistry-on-the-complex” approach.

## Abstract 2

A modular chemistry-on-the-complex approach enabled the divergent synthesis of a series of (multi)donor-photosensitizer-acceptor via Suzuki–Miyaura cross coupling from a functionalized Ru photosensitizer unit. After excitation, the formation of the charge separation was confirmed by transient absorption spectroscopy, despite the incomplete formation assigned to the marginal driving force. In contrast to the unimolecular donor triad, a polymer-based donor revealed a significant prolongation of the charge separation into the microsecond time scale.

## Introduction

**Scheme 1:**
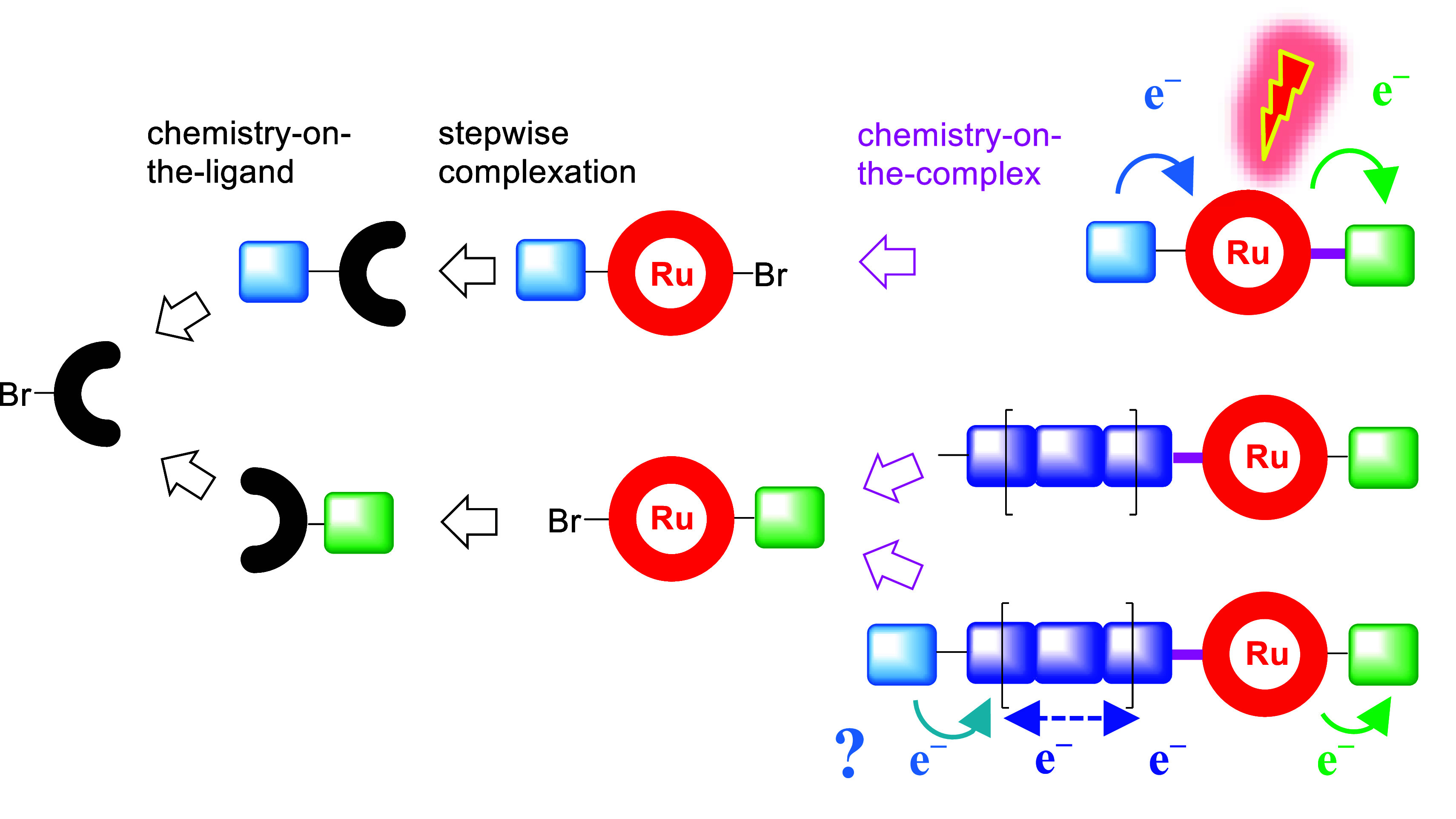
Simplified Schematic Representation of the Retrosynthetic Modular Assembly and Photo-Induced Charge Separation Via sequential “chemistry-on-the-ligand”, stepwise complexation, and “chemistry-on-the-complex” for late-stage diversification. Typically via excitation, oxidative quenching and secondary electron transfer in molecular systems (top right), (vectorial) charge transport with polymer-based multidonor and/or terminal donor sites (bottom, right).

Modern functionalized Ru(II)-polypyridyl-type complexes enable the efficient modular synthesis of photoredox-active assemblies for vectorial electron transfer, descending from both their beneficial photophysical properties and inherent structural features. The archetypical sensitizer [Ru(bpy)_3_]^2+^ (bpy is 2,2′-bipyridine) combines strong absorption in the visible light region, long excited state lifetimes (τ = 1000 ns in the absence of oxygen) and attractive redox properties for subsequent charges separation,^1^ while the [Ru(tpy)_2_]^2+^ (tpy is 2,2′:6′,2″-terpyridine) congener enables the facile construction of linear donor-photosensitizer-acceptor (D–P–A) assemblies but suffers from short-lived excited state lifetimes (τ = 0.25 ns).^2^ The latter limitation was alleviated by an enhanced bite angle of the tridentate ligand,^3^ leading to a remarkably long-lived excited state (τ = 3 μs) for the prototypical [Ru(dqp)_2_]^2+^ (dqp is 2,6-di(quinolin-8-yl)pyridine) complex.^4^ The utility of this sensitizer for vectorial photoinduced charge separation was demonstrated for “molecular” D–P–A triads.^5,6^ The schematic presentation of D–P–A assemblies are depicted in Scheme 1 (top, right). After excitation, primary and secondary electron transfer steps lead to a fully charge-separated (CS) state with the oxidized donor and the reduced acceptor site. The axial arrangement of donor and acceptor at the central Ru^II^ ensures maximum spatial separation for long-lived CS. In addition, the linkage pattern and mutual distance dictate the degree of interaction and, thus, affect the precise energy levels and rate constants of the electron transfer steps.

The first example of a [Ru(dqp)_2_]^2+^-based triad featured a methylene-bridged donor and a xylyl- or amide-tethered acceptor unit, leading to a charge fully charge-separated (CS) state with the oxidized donor and the reduced acceptor site with a CS lifetime of 140 or 200 ns, respectively.^5^ In the meantime, Wenger and co-workers reported 10-fold prolonged charge separation lifetimes and the associated quantum yields in two molecular triads in order to discriminate energy-storing and energy-wasting electron transfer reactions.^6^ In view of exploiting the generated charges,^6^ e.g., in coupled catalytic processes,^8^ further charge transport and/or charge accumulation by additional functional units are required. Toward this task, the single donor (D) and acceptor (A) units were replaced by redox-active side-chain-decorated polymers (D_*n*_ and A_*m*_) resulting in prolonged charge separation lifetimes in the microsecond range (400 and 2400 ns).^9^ Next, replacing the donor polymer with a conjugated poly(carbazole) further prolonged the charge separation lifetime (700 and 7200 ns).^10^ The biexponential kinetics indicate that at least two recombination pathways exist, which appears plausible in view of the conformational flexibility and multiple redox units (*n* and *m*) in the donor (D_*n*_) and acceptor polymer (A_*m*_). Notably, the recently reported simple isolation of strict monodisperse redox-active oligomers^11^ will offer the opportunity to tune charge separation with the highest possible stoichiometric precision in such assemblies. Prior to this, we embarked on exploring structurally more defined assemblies based on a preserved “–P–A” subunit featuring a single “molecular” donor site, a “polymer”-based multidonor, or the combination of both by a single terminal donor attached to the multidonor.

In this work, we explore the modular synthetic building block approach for the syntheses of photosensitizer assemblies with different donor moieties on a preassembled “–P–A” subunit. In this series, the role of the donor subunit will be assessed and the effect of a single vs multiple acceptor unit will be discussed. For this goal, the retrosynthetic analysis identified a single ligand precursor (Scheme 1), followed by diversification via “chemistry-on-the-ligand”, stepwise complexation, and “chemistry-on-the-complex”^12^ to combine design flexibility and minimal synthetic efforts. Hence the identical linkage pattern within the series is preserved, which is often challenging to achieve and/or limited by synthetic constrains,^6^ but is highly advantageous for fully consistent data interpretation in terms of structural and electronic features of the connectivity between subunits (vide infra). The Pd-mediated borylation and subsequent Suzuki–Miyaura cross-coupling reactions in the final steps are explored, including a simplified purification process, which constitute to date a major practical challenge to prepare efficiently novel photoredox-active assemblies. To warrant for a rational development and a comprehensive analysis with respect to scattered preceding reports, we deliberately selected as donor units a molecular triarylamine-based moiety (TARA), or an end-functionalized poly(carbazole)-based multidonor with a tolyl- or a TARA-end group. We will demonstrate that long-lived charge separation up to a few μs can be achieved by implementing a polymer-based donor which enables the systematic future rational design without excessive synthetic burdens.

## Results and Discussion

### Synthetic Approach

The modular assembly toward the triads consists of three stages (Scheme 1): (i) diversification of the bromide functionalized ligand (dqp-Ph-Br) via Pd-catalyzed borylation and subsequent Suzuki–Miyaura coupling with a halide-decorated NDI (NDI is *N*-(2′-ethylhexyl)-naphthalene-1:8,4:5-diimide-*N*′-yl) or TARA (TARA is *N*,*N*-bis(4-methoxyphenyl)aniline-4-yl) unit, respectively, (ii) stepwise complexation using first the dqp-Ph-Br ligand and second the NDI- or TARA-decorated ligands from the previous step, and (iii) the late-stage functionalization applying the Pd-mediated borylation and Suzuki–Miyaura coupling with the desired complementary redox active unit. For this task, the protocols and some intermediates from a previous report were utilized.^7^ For simplicity, architectures will be abbreviated as, e.g., Br-Ph/Ph-Ph-NDI for [(Br-Ph-dqp)Ru(dqp-Ph-Ph-NDI)]^2+^, i.e., “/” denotes [(-dqp)Ru(dqp-)]^2+^.

### Chemistry-on-the-Ligand

**Scheme 2:**
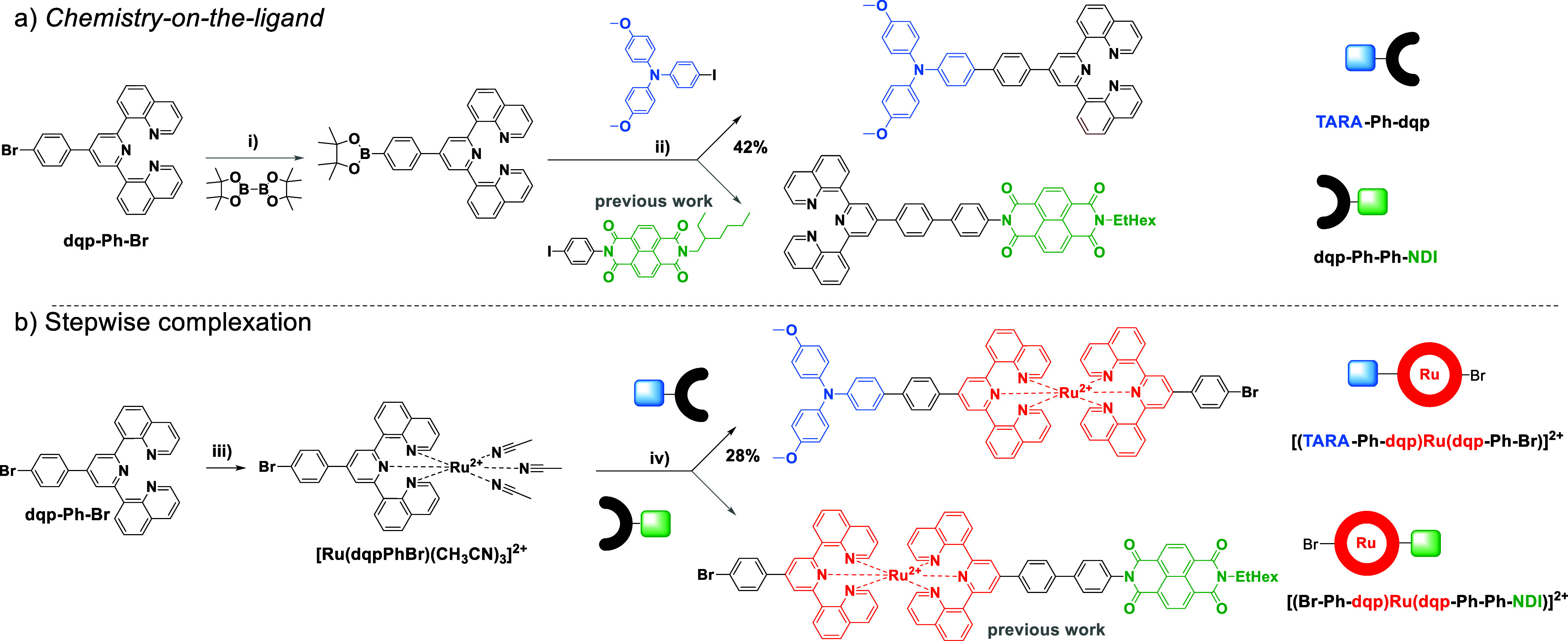
Schematic Representation of Chemistry-on-the-Ligand and Stepwise Complexation (i) Pd(dppf)Cl_2_, KOAc, DMSO, 80 °C, 6 h. (ii) SPhos Pd G3, SPhos, K_3_PO_4_, toluene/H_2_O 20:1, 50 °C, overnight. dqp-Ph-B(pin) and dqp-Ph-Ph-NDI reproduced for clarity (gray arrows).^7^ (iii) [RuCl_3_(iPrSPh)_2_(MeOH)], CH_3_CN, 130 °C, μwave irradiation, 2.5 h, then + AgNO_3_, EtOH, H_2_O, 80 °C, overnight. (iv) DMF, 120 °C, 2 d. [(Br-Ph-dqp)Ru(CH_3_CN)_3_]^2+^ and [(Br-Ph-dqp)Ru(dqp-Ph-Ph-NDI)]^2+^ reproduced for clarity (gray arrow).^7^ (a) Synthesis to donor-/acceptor-functionalized ligands via “chemistry-on-the-ligand” and (b) “stepwise complexation” with a Br-functionalized monotridentate Ru intermediate.

The bromo-functionalized ligand (dqp-Ph-Br) serves as the universal building block for Pd-mediated reactions. First, the selective borylation using a commercially available diboron reagent facilitated the introduction of the boronic ester unit.^6^ Due to the anhydrous conditions, no undesired dimerization via potential Suzuki–Miyaura cross-coupling was observed. Next, the boronic pinacol ester intermediate (dqp-Ph-Bpin) was converted under aqueous Suzuki–Miyaura coupling conditions with a TARA-I (Scheme 2a top) or a Ph-I-decorated NDI unit (Scheme 2a bottom).^7^ In line with previous results, the yield for the electron-rich donor moiety (42%) is significantly lower than the one reported for the electron-deficient acceptor moiety (86%).^7^ This finding may be explained by the lowered rate of oxidative addition for the electron-rich TARA-substrate,^13^ or undesired side reactions.

**Scheme 3:**
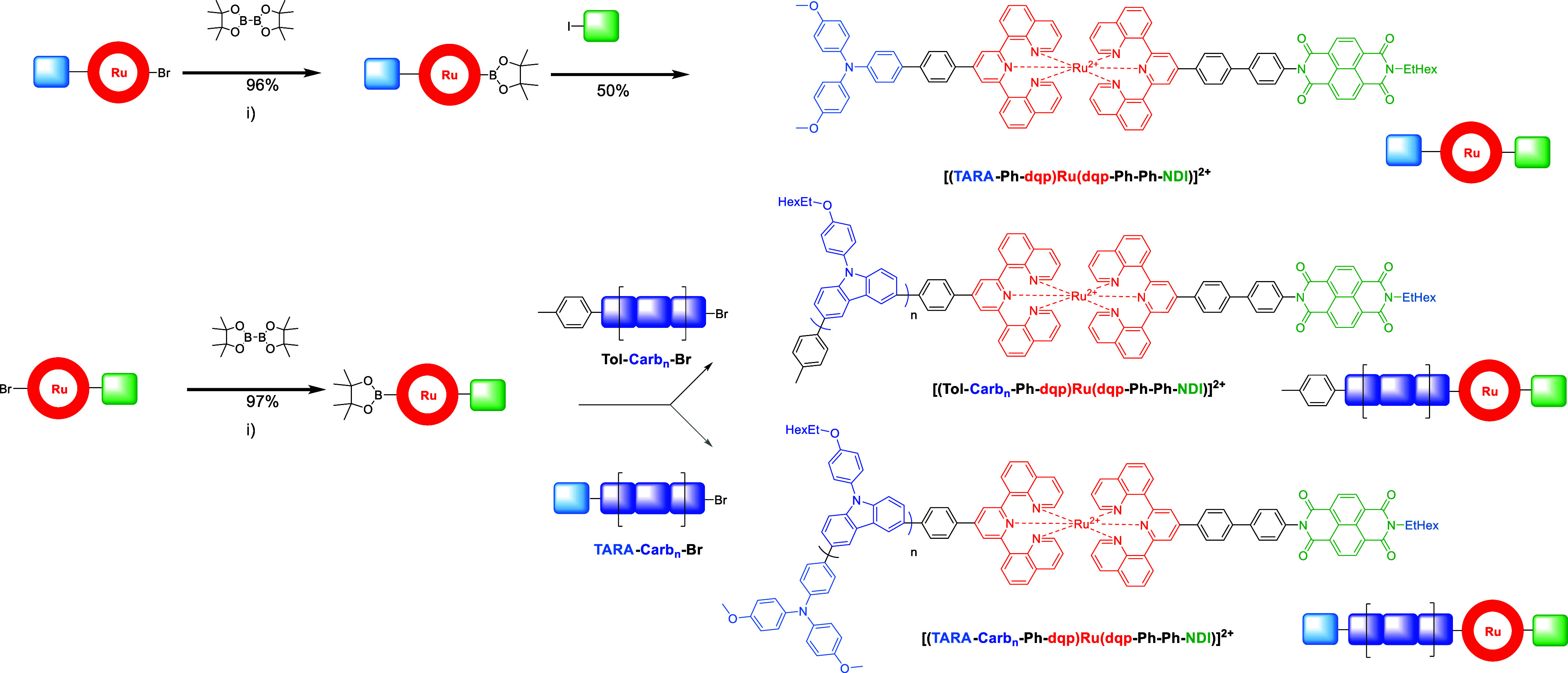
Schematic Representation of the Triad and Tetrad Syntheses via Chemistry-on-the-Complex Using Borylation/Suzuki–Miyaura Cross-Coupling Steps (i) Pd(dppf)Cl_2_, KOAc, DMF, 80 °C, overnight. (ii) RuPhos Pd G3, RuPhos, K_3_PO_4_, THF/H_2_O 20:1, 50 °C, overnight.

### Stepwise Complexation

With this ligand set in hand, the stepwise complexation was performed. First, the coordination of one dqp-Ph-Br ligand leads to the *mono*-tridentate Ru intermediate, which was subsequently converted to the heteroleptic bis-tridentate Ru dyads bearing TARA (Scheme 2b top) or as previously reported for NDI units, respectively (Scheme 2b, bottom). Notably, the purification of the two dyads required manual aqueous column chromatography (SiOH, CH_3_CN/H_2_O/KNO_3(aq., sat.)_ 40:4:1), anion metathesis and subsequent crystallization to remove minute impurities. Despite the low yields (28% for TARA-Ph/Ph-Br), the benefit arises in subsequent reaction steps with simplified purification and higher yields (vide infra). In addition to the heteroleptic complexation, a 2-fold complexation with dqp-Ph-Br starting from [Ru(CH_3_CN)_6_]^2+^ yielded the homoleptic complex (see Supporting Information) as a reference compound for the photophysical characterization (vide infra).

### Chemistry-on-the-Complex

With the two bromo-functionalized dyads in hand, the introduction of the complementary redox-active moieties was performed (Scheme 3). Notably, the same borylation/Suzuki–Miyaura cross-coupling methodology as for the chemistry-on-the-ligand can be applied (vide supra),^6^ as detailed recently for a related [Ru(dqp)_2_]^2+^ based complex.^7^ The bromo-group of the donor dyad (TARA-Ph/Ph-Br) was converted into the respective boronic pinacol ester (TARA-Ph/Ph-Bpin) complex under anhydrous conditions in excellent yield (96%). Notably, the isolation required only washing with aqueous NaPF_6_ and water as well as precipitation in diethyl ether. The molecular triad was obtained upon coupling with the NDI-Ph-I acceptor unit in good yields (50%). In contrast to the stepwise complexation stage, the isolation of the target compound was achieved by flash column chromatography using organic eluent mixtures (SiOH, CH_2_Cl_2_/MeOH) instead of cumbersome manual aqueous chromatography (vide supra), however, the lower yield is ascribed to loss upon purification. In analogy, the acceptor dyad (Br-Ph/Ph-Ph-NDI) was converted into the boronic ester intermediate and subsequently coupled to α-,ω-decorated poly(carbazole)s, which were readily prepared according to literature procedures.^14^ An excess of the bromo-equipped polymer was used to maximize the functionalization of the dyad complex, and the target compounds were obtained applying the same general purification procedure to also remove unreacted polymer remainings. It is noteworthy to highlight the simplicity and success of the chemistry-on-the-complex approach, which depends on the removal of unreacted dicationic Ru complexes as well as noncharged organic compounds. Notably, the presence of an organo-solubilizing polymer chain simplified the chromatographic purification with respect to the complexation stage, i.e., nonaqueous salt-free eluents can be used directly without the need of anion metathesis. A similar beneficial effect has been observed for multiarylated [Ru(dqp)_2_]^2+^-based complexes.^15^ In summary, the chemistry-on-the-complex strategy ensures identical linkage patterns as well as a minimal number of synthetic steps including simplified isolation protocols and high yields. Hence, this late-stage diversification complements existing routes and circumvents many of the inherent synthetic limitations.^6,12^

The identity and purity of the Ru dyads and triads were confirmed by ^1^H NMR-spectroscopy (Figures S1–S23), HR-ESI-ToF or MALDI-ToF mass spectrometry (Figures S24–S35), and analytical size-exclusion chromatography (SEC) coupled to UV–vis detection (Figures S36–S45, Table S1). In general, the ^1^H NMR spectra display the characteristic proton resonances of the subunits, i.e., the NDI unit, the TARA-unit, and Ru sensitizer, as well as the corresponding Bpin-intermediates with matching integral ratios. The HR-ESI-ToF data directly confirm the composition of the target compounds (TARA-Ph/Ph-Ph-NDI in Figure 1a, top), evidencing the clean borylation and successful subsequent Suzuki–Miyaura coupling step on the basis of absent detectable amounts of Ru byproducts as well as matching experimental and simulated isotopic patterns of the target structures.

**Figure 1:**
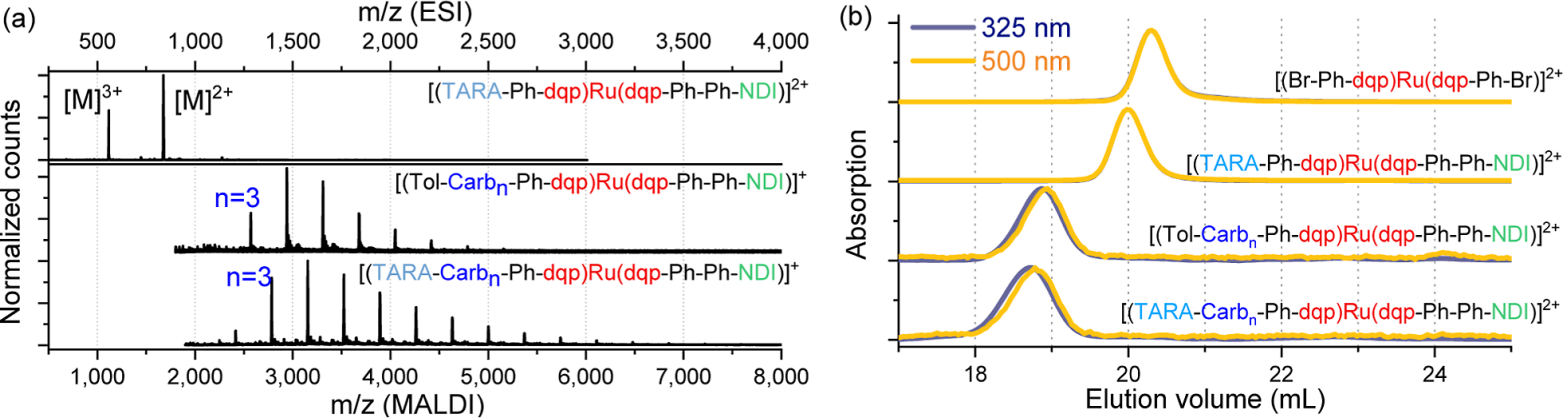
(a) Normalized HR-ESI-ToF and MALDI-ToF mass spectra (DCTB + NaI): (top) molecular triad TARA-Ph/Ph-Ph-NDI, (middle) polymer-based triad Tol-Carb_*n*_-Ph/Ph-Ph-NDI, and (bottom) polymer-based tetrad TARA-Carb_*n*_-Ph/Ph-Ph-NDI. See Supporting Information for full assignments. (b) Offset normalized size-exclusion chromatograms at 325 nm (blue) and 500 nm (yellow) detection (DMAc +0.08 wt % NH_4_PF_6_) of the reference complex Br-Ph/Ph-Br, molecular triad TARA-Ph/Ph-Ph-NDI, the polymer-based triad Tol-Carb_*n*_-Ph/Ph-Ph-NDI, and the polymer-based tetrad TARA-Carb_*n*_-Ph/Ph-Ph-NDI (top to bottom). Note the superposition of both wavelength traces for the molecular reference complex and triad, and the systematic effect of the chain length (*n*) as additional absorbance at 325 nm leading to an apparent shift toward shorter elution volumes. See Supporting Information (Chapter 7 and Scheme S1) for more details.

In case of the polymer triad and tetrad, MALDI-ToF mass spectrometry was applied (Figure 1a, middle/bottom). A monomodal distribution was observed with an apparent maximum assigned to 4 carbazole repeat units assuming negligible mass discrimination effects. More importantly, the successful linkage is proven by isotope simulations of the intact assemblies. The compounds were also analyzed by SEC using a UV/vis detector from 250 to 600 nm.

Figure 1b displays representative wavelengths in the UV (325 nm) and vis region (500 nm). In all cases, the characteristic ^1^MLCT absorption around 500 nm was detected, as well as π–π* transitions around 325 nm. In the case of the molecular reference complex Br-Ph/Ph-Br and the molecular triad TARA-Ph/Ph-Ph-NDI, the anticipated perfect superposition of the 325 nm and the 500 nm trace was observed. In the case of the polymer-based triad and tetrad, an apparent offset of the 325 nm trace toward lower elution volumes was observed. This observation is readily explained by the higher relative contribution of the (multi)donors to the overall absorption intensity in the UV region, as expected for a larger number of repeating units and, thus, a lower resulting elution volume (see Supporting Information, Chapter 7 and Scheme S1 for explanation). The closer inspection of the polymer-based triad and tetrad indicates a slightly larger average chain length for the tetrad, in qualitative agreement with the broader envelope of the MALDI-ToF distribution found for higher molar masses. In summary, the analytical characterization results unambiguously confirmed the successful modular linkage via the chemistry-on-the-complex approach. This simple opportunity extends the modular approach to connect, to purify, and to analytically confirm the linkage of tailor-made polymer blocks with photosensitizer units, with preserved linkage pattern to provide a consistent series to elucidate light-induced charge separation and descending processes (vide infra).

### (Spectro-)Electrochemical Analysis

The electrochemical potentials of the redox-active subunits were determined by cyclic voltammetry in dichloromethane containing 0.1 M TBAPF_6_ vs ferrocene^I/0^ as reference. The molecular triad TARA-Ph/Ph-Ph-NDI reveals the typical redox processes in accordance with related literature data (Figures S46 and S47a).^10,16,17^ The mildest reduction occurs at the NDI unit at −1.05 V (NDI^0/–I^), while the [Ru]-ligand-centered reduction is found at −1.71 V ([Ru]^II/I^). The mildest oxidation is assigned to TARA at +0.23 V and the [Ru]-based oxidation ([Ru]^III/II^) occurs at +0.73 V (Table 1, entry A). In case of the polymer-based triad Tol-Carb_*n*_-Ph/Ph-Ph-NDI, the formal half-wave-potentials of the carbazole polymer’s oxidation is assigned to ca. +0.31 V, which is cathodically shifted by approximately 100 mV in agreement with reported values upon replacement of the *N*-alkyl- by an *N*-*para*-anisyl substituent (Table 1, entry B).^18^ Notably, such an assignment of the one-electron oxidation potential for polymers should be treated with caution, since the electrochemical response represents a superposition of accumulative redox processes (vide infra).^19^ In case of the polymer-based tetrad TARA-Carb_*n*_-Ph/Ph-Ph-NDI, the single TARA-end group undergoes oxidation at ca. +0.17 V (Table 1, entry C, Figure S47b), which is slightly shifted with respect to the molecular triad due to the influence of the electron-withdrawing [Ru]-fragment vs electron-releasing carbazole units. These assignments were confirmed by spectro-electrochemical analysis, which revealed the formation of the TARA radical cation (at 445 and 710 nm) prior to Carb_*n*_-centered oxidation (at 414 nm and >900 nm) (Figure S48b). Notably, the electrochemical properties were found fully consisted with the corresponding subunits, highlighting that assemblies can be designed from the building block without undesired alteration of the redox properties.

**Table 1:** Electrochemical Data of [Ru(dqp)_2_]^2+^-Based Triads Including Reference Data^a^

entry	ref.	Ru^II/I^ (V)	*A* (V)	*D* (V)	Ru^III/II^ (V)
A	TARA-Ph/Ph-Ph-NDI	–1.71	–1.05	0.23	0.73
B	Tol-Carb_*n*_-Ph/Ph-Ph-NDI	–1.71^b^	–1.05^b^	0.31^c^	0.73^b^
C	TARA-Carb_*n*_-Ph/Ph-Ph-NDI	–1.71^b^	–1.05^b^	0.17^c^	0.73^b^
D	PTZ/Xyl-BQ^[^^5^^]^	–1.70^d^	–0.80^d^	0.40^e^	0.71
E	PTZ/amide-BQ^[^^5^^]^	–1.74^d^	–0.78^d^	0.38^e^	0.73
F	Carb’_*n*_-Bn-trz-Ph/O-Bn-NDI_*m*_^[^^10^^]^	–1.79^f^	–1.03^g^	0.22^c^	0.63^h^
G	TARA’_*n*_-Bn-O/Ph-trz-Bn-NDI_*m*_^[^^9^^]^	–1.79^f^	–1.03^g^	0.20^i^	0.63^h^

a Data from cyclic voltammetry in CH_2_Cl_2_ if not stated otherwise.

b Taken from TARA-Ph/Ph-Ph-NDI and assumed to be invariant.

c CV data from D_*n*_.

d DPV data in CH_3_CN.

e CV data in CH_3_CN.

f CV data of P–A_*n*_ in DMF from ref (20).

g DPV data of A_*n*_ in DMF from ref (21).

h ^i^DPV data of P–D_*n*_ in CH_2_Cl_2_ from ref (21).

i Taken from ref (17). NDI is *N*-(2′-ethylhexyl)-naphthalene-1:8,4:5-diimide-*N*′-yl. PTZ is phenothiazine-*N*-methylene-yl. BQ is benzoquionone-2-yl. Carb_*n*_ is poly(*N*-(4′-(2″-ethylhexyloxy)phenyl)-carbazole-3,6-diyl). Carb’_*n*_ is poly(*N*-(2′-ethylhexyl)-carbazole-3,6-diyl). TARA is *N*,*N*-bis(4-methoxyphenyl)aniline-4-yl. TARA’_*n*_ is poly(4-(*N*,*N*-bis(4′-butylphenyl)amino)styrene). “/” denotes [(−Phdqp)Ru(dqpPh−)]^2+^ framework. See Scheme 4b for visualization of state diagrams. For discussion of literature-reported data, see Table 3 and text.

### Steady State Absorption and Emission

**Figure 2:**
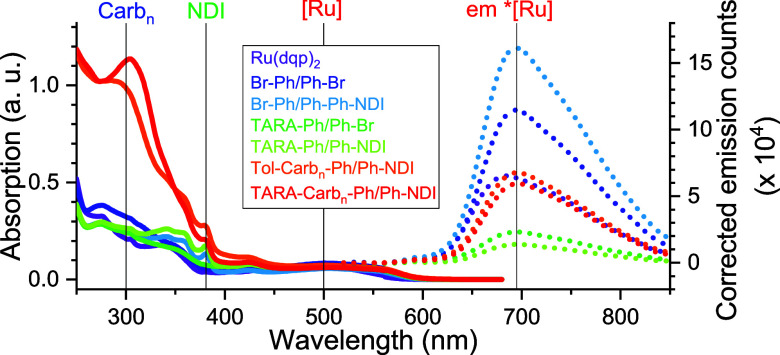
Normalized baseline-corrected absorption (solid) and baseline- and absorption-corrected emission spectra (dotted) of dyads/triads and references (in CH_2_Cl_2_ after multiple freeze–pump–thaw cycles). Black lines indicate characteristic bands.

Figure 2 depicts the UV/vis absorption profiles of the functionalized Ru complexes featuring a broad ^1^MLCT band centered around 500 nm including a bathochromic shoulder around 550 nm due to the π-extended ligand framework in comparison to [Ru(dqp)_2_]^2+^.^15,16^ In the presence of NDI units, the corresponding π–π* transition band is found at around 381 nm, while the poly(carbazole) motif leads to intense absorption around 300 nm.^10^ Hence, the donor and acceptor units’ absorbance occur in the UV region, enabling the excitation at 500 nm to selectively populate the [Ru]-sensitizer’s ^1^MLCT manifold, followed by intersystem crossing to reach the intrinsically long-lived emissive ^3^MLCT state(s).^4^ The steady state emission spectra of the complexes are centered around 695 nm, i.e., a comparable ^3^MLCT excited state energy (1.78 eV) at room temperature is found for the series. This finding is in line with previous studies, which revealed a similar energetic stabilization behavior due to the π-extended ligand framework of the phenylene units with respect to the nonfunctionalized [Ru(dqp)_2_]^2+^ (685 nm, 1.81 eV).^15,16^ The effect of substituents on the ^3^MLCT emission quantum yields can be ascribed to two effects: (a) π-extended ligand frameworks generally lead to an increased quantum yield, and (b) electron-releasing substituents typically lower the quantum yield.^16^ Indeed the Br-Ph/Ph-Br-decorated complex features a 1.7-fold emission quantum yield compared to [Ru(dqp)_2_]^2+^. In line, replacing one bromide with the electron-donating TARA unit reduces the emission quantum yield. Contrary, the replacement of one bromide by Ph-NDI leads to enhanced emission instead—in qualitative agreement with a more extended π-system and suggesting inefficient oxidative quenching in line with time-resolved data (vide infra). Hence, the Br-Ph/Ph-Ph-NDI dyad serves as the common reference fragment to assess the effect of the different donor units in the corresponding triads. Replacing the remaining bromide leads to significantly reduced emission quantum yields of the D–P–A assemblies in the order TARA (9%), TARA-Carb_*n*_ (37%), and Tol-Carb_*n*_ (42%) and, thus, correspond to quenching efficiencies of 91%, 63%, or 58% with respect to Br-Ph/Ph-Ph-NDI (Table 2, relative Φ_em_).

**Table 2:** Photophysical Properties of Studied Sensitizer, Dyads, Triads and Tetrad^a^

entry	type	substituent pattern	relative Φ_em_^b^	τ_em_ (wavelength)	τ_TA_ (wavelength)	global fit τ_TA_ (state)^c^
A	P′	Ru(dqp)_2_	0.41	1.99 μs (670 nm)	1.98 μs (470 nm)	n.d
B	P	Br-Ph/Ph-Br	0.71	1.63 μs (690 nm)	1.68 μs (470 nm)	n.d
C	P–A	Br-Ph/Ph-Ph-NDI	1.00	1.72 μs (670 nm)	1.77 μs (470 nm)	1.74 μs (aMLCT)
D	D–P	TARA-Ph/Ph-Br	0.14	1.66 μs (670 nm)	1.71 μs (470 nm)	n.d
E^d^	D–P	TARA-Ph/Ph-Br	n.d	3.11 μs (700 nm)	3.24 μs (470 nm)	2.85 μs (aMLCT)
F	D–P–A	TARA-Ph/Ph-Ph-NDI	0.09	1.20 μs (690 nm)	0.17 μs (450 nm)^e^	0.18 μs (CS) & 1.14 μs (aMLCT)^f^
					0.15 μs (580 nm)^e^	
G^d^	D–P–A	TARA-Ph/Ph-Ph-NDI	0.07^g^	2.89 μs (700 nm)	0.32 μs (450 nm)^e^	0.28 μs (CS) & 2.49 μs (aMLCT)^f^
				2.51 μs (530–800 nm)^c^	0.32 μs (580 nm)^e^	
H	D’_n_–P–A	Tol-Carb_n_-Ph/Ph-Ph-NDI	0.42	2.28 μs (690 nm)	2.34 and 13.2 μs (450 nm)^e^^,^^f^	1.84 μs (aMLCT) & 8.44 μs (CS)^f^
I	D–D’_n_–P–A	TARA-Carb_n_-Ph/Ph-Ph-NDI	0.37	1.30 μs (670 nm)	1.94 and 13.5 μs (450 nm)^e^^,^^f^	0.59 μs (aMLCT) & 2.38 μs (CS)^f^

a Conditions: CH_2_Cl_2_ (after multiple freeze–pump–thaw cycles if not stated otherwise).

b Relative emission quantum yield referenced vs Br-Ph/Ph-Ph-NDI.

c From global fit, spectral assignment of charge-separated (CS) state or ^3^MLCT state.

d Sample prepared in oxygen-free glovebox atmosphere.

e Assigned to charge-separated state confirmed by isosbestic points of Br-Ph/Ph-Ph-NDI (entry C) or TARA-Ph/Ph-Br and associated spectral signatures.

f Unconstrained biexponential fit assigning one lifetime to emission and one to charge recombination.

g Referenced vs TARA-Ph/Ph-Br (0.14). “n.d.” denotes not determined.

### Transient Absorption and Emission

The assigned extent of emission quenching was assigned to charge-separation for the moment. To safely assign the charge-separated states, transient absorption and emission spectra were recorded and analyzed. At longer wavelengths (>580 nm), the TA signature of the ^3^MLCT is generally positive but overlapped with stimulated emission centered at 700 nm (see Figure S50). In line with the lower steady state emission quantum yield of the TARA-Ph/Ph-Br dyad vs the Br-Ph/Ph-Ph-NDI dyad, the contribution of stimulated emission is lower as evident from the shift of the isosbestic points closer to 700 nm (Figure S50a vs Figure S50b). The TA spectral signatures resemble that of a typical ^3^MLCT ground state recovery with a similar monoexponential time constant as the ^3^MLCT emission decay (Table 2), supporting the previous assignment of substituent effects on the steady state emission quantum yield (vide supra). Notably, the possibility of ultrafast electron transfer and recombination in the case of the TARA-Ph/Ph-Br dyad is unlikely on the basis of the driving force estimation, which would suggest in that case even more pronounced quenching of the Br-Ph/Ph-Ph-NDI dyad (vide infra, Table 3). As a consequence, the difference in the observed emission quantum yields is assigned to the subtle interplay of the inherent radiative and nonradiative ^3^MCLT decay pathways.

**Table 3:** Driving Forces for Primary and Secondary Electron Transfer Steps^a^

entry	ref.	Δ*G*_ox_ (eV)	Δ*G*_red_ (eV)	Δ*G*_CS_^b^ (eV)	*E*_CS_ (V)
**I**_**a**_	PTZ/Xyl-BQ^[^^5^^]^	–0.24	+0.35	–0.31	1.20
**I**_**b**_	PTZ/amide-BQ^[^^5^^]^	–0.27	+0.34	–0.35	1.16
**II**_**a**_	TARA’_*n*_-Bn-O/Ph-trz-Bn-NDI_*m*_^[^^9^^]^	–0.09	+0.22	–0.43	1.23
**II**_**b**_	Carb’_*n*_-Bn-trz-Ph/O-Bn-NDI_*m*_^[^^10^^]^	–0.09	+0.24	–0.41	1.25
**III**_**a**_	TARA-Ph/Ph-Ph-NDI	±0.00	+0.16	–0.50	1.28
**III**_**b**_	Tol-Carb_*n*_-Ph/Ph-Ph-NDI	±0.00	+0.24	–0.42	1.36
**III**_**c**_	TARA-Carb_*n*_-Ph/Ph-Ph-NDI	±0.00	+0.10	–0.56	1.22

a Calculated from redox properties and emission energies (see Table 1).

b Formal driving force of secondary charge transfer after oxidative quenching to form fully charge-separated (CS) state.

**Figure 3:**
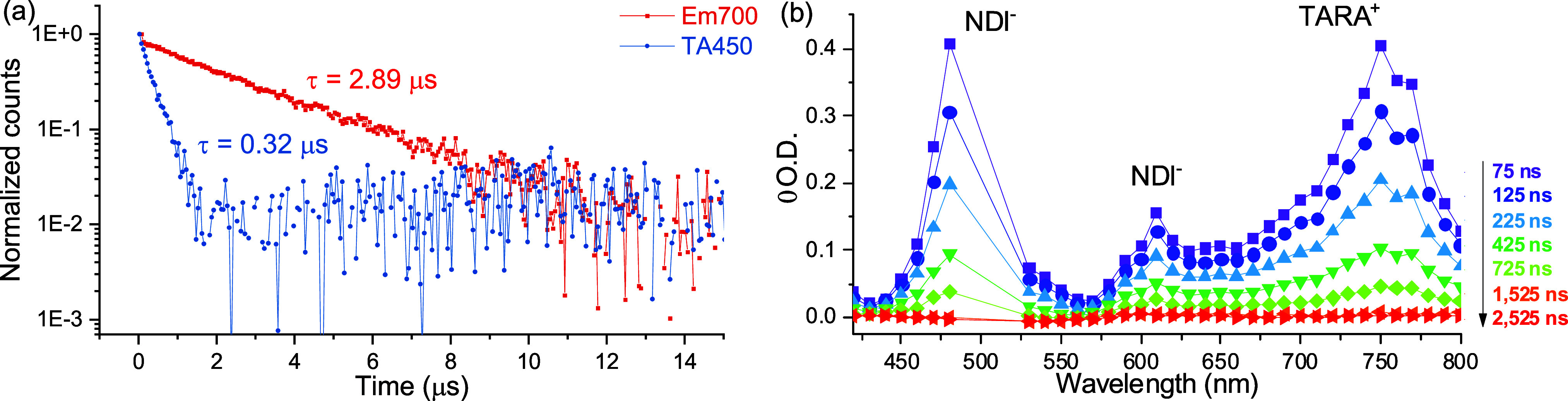
(a) Normalized ^3^MLCT emission decay at 700 nm (red) and TA decay characteristic for CS at 450 nm (blue) to illustrate monoexponential decay (logarithmic scale). See text for explanation. (b) Transient absorption spectra of molecular TARA-Ph/Ph-Ph-NDI triad featuring the charge-separated state based on spectroscopic NDI^–^ and TARA^+^ signatures. Data from glovebox-sample. Assignments to NDI^–^ and TARA^+^ according to spectro-electrochemical measurements [Figure S48 (TARA^+^) and ref (21) (NDI^–^)].

Next, the molecular triad TARA-Ph/Ph-Ph-NDI was examined (Table 2, entries F/G). The emission trace was monoexponential (Figure 3a) with a slightly lower lifetime than the reference compounds (1.20 μs, see Figure S50). In order to check whether different residual oxygen content causes the shorter lifetime, a fresh sample was prepared in a glovebox and measured. Attributed to the negligible oxygen content, the apparent emission lifetime indeed increased to 2.89 μs (Figure 3a) as expected for diffusional quenching at long time scales. Reassessment of the corresponding donor dyad confirmed this effect. The transient absorption spectra of TARA-Ph/Ph-Ph-NDI (Figure 3b) are dominated by the characteristic spectral features of the NDI radical anion (at 475 and 610 nm) and the TARA radical cation (at 750 nm). The decay profiles at the isosbestic points of the ^3^MLCT (450 or 580 nm) were monoexponential with a lifetime of 0.32 μs. This value corresponds well to that of the related molecular triads (PTZ/Xyl-BQ and PTZ/amide-BQ)^5^ as well to the short-lived component for related ExTTF-Xyl/Xyl-cNDI triad.^6^ The biexponential global fit of the TA data confirmed the short-lived component as the CS state and the long-lived component as the ^3^MLCT state identified by the [Ru] ground state bleach (around 500 nm) (see Figure S49a). Based on the reported NDI radical anion absorption (Δε_474nm_ (NDI^–^) = 3.0 × 10^4^ M^–1^ cm^–1^, in DMF)^22^ and excited state absorption of *[Ru(dqp)_2_]^2+^ (Δε_500nm_ (*[Ru(dqp)_2_]^2+^) = −1.1 × 10^4^ M^–1^ cm^–1^, in CH_3_CN),^5^ the ratio of CS and ^3^MLCT can be estimated. According to the components’ amplitudes, the contribution of ^3^MLCT (10%) vs CS (90%) agrees well with the observed residual steady state emission (0.09) with respect to the Br-Ph/Ph-Ph-NDI dyad.

In order to further test the effect of tentatively different oxygen contents on the CS state, the TA data of the original sample was evaluated. Consistently, the long-lived component was identified as the ^3^MLCT state based on the typical spectral features and the typical lifetime (1.14 μs, see Figure S49a). Similarly, the short-lived fully CS state displayed a reduced lifetime (0.16 μs) as derived from the isosbestic points (450 and 580 nm). Based on the reported NDI radical anion absorption and excited state absorption of *[Ru(dqp)_2_]^2+^, the CS state dominates the TA profile but the fraction of the ^3^MLCT was found higher (approximately 30%). These results parallel the meanwhile published report of Wenger and co-workers, which assigned additional processes to electron transfer in their studied triads.^6^

**Figure 4:**
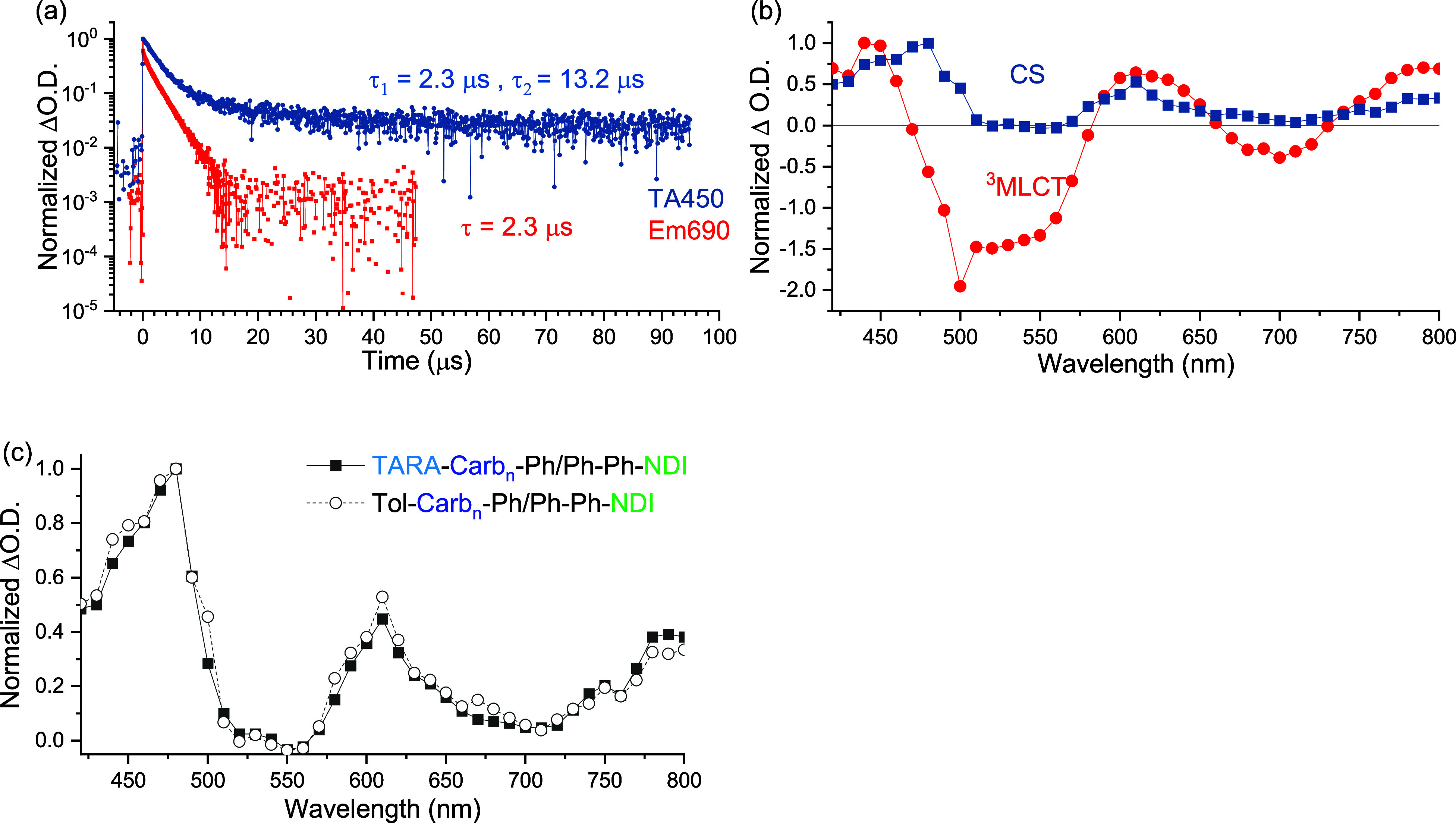
Time-resolved and spectral data for Tol-Carb_*n*_-Ph/Ph-Ph-NDI triad in CH_2_Cl_2_: (a) decay profile to illustrate residual monoexponential emission decay (red) and nonmono-exponential decay at selected wavelength (logarithmic scale). Data from freeze–pump–thawed sample. (b) Normalized decay associated spectra from biexponential fit with characteristic spectral signatures of the ^3^MLCT (red) and charge-separated state (blue). (c) Comparison of the long-lived component obtained for TARA-Carb_*n*_-Ph/Ph-Ph-NDI and Tol-Carb_*n*_-Ph/Ph-Ph-NDI showing no evidence of TARA^+^ formation around 750 nm (see TARA-Ph/Ph-Ph-NDI, Figure 3).

Finally, the polymer-based triads were investigated (Table 2, entries H/I). In line with the results of the previous assignments, monoexponential ^3^MLCT emission decay in the microsecond time scale (2.28 μs, glovebox prepared samples) was identified for Tol-Carb_*n*_-Ph/Ph-Ph-NDI (Figure 4a, red trace). In analogy to the related all-polymer triads,^9,10^ a biexponential fit was applied. Importantly, the transient absorption decay at the ^3^MLCT isosbestic point (450 nm) revealed charge separation lifetimes of 2.34 μs (91%) and 13.2 μs (9%) (Figure 4a, blue trace). The spectrum of the CS state (Figure 4b) revealed the characteristic signatures of the NDI radical anion (475 and 610 nm) and the onset of the broad carbazole radical cation (above 650 nm).

The TARA-decorated polymer-based tetrad (TARA-Carb_*n*_-Ph/Ph-Ph-NDI), which contains a TARA unit at the polymer chain end, was examined to elucidate any role of the terminal donor. The analysis as described above revealed a surprisingly similar behavior, i.e., comparable biexponential recombination kinetics in the 450 nm transient absorption trace of 1.94 and 13.5 μs (Figure S54) and identical spectral signatures of the long-lived CS state (Figure 4c, filled squares vs open circles) were found. Although the CS state was more prominent in these TA spectra compared to the polymer-based triad, no sign of the oxidized TARA unit (around 750 nm) was observed in the tetrad assembly, as expected from the milder oxidation potential of TARA vs Carb_*n*_ and spectro-electrochemical data (vide supra). In view of the same recombination kinetics in the ns time domain, no electron transfer from the polymer Carb_*n*_ to the terminal donor TARA can be assigned beyond the long lifetime of the CS state.

### Comparison of Molecular and Polymer-Based Assemblies

In the following, the photoinduced charge separation as a parameter of the P–A interaction and the recombination processes as a parameter of the donor will be discussed and compared with related molecular triads (Table 3, **I**_**a**_ and **I**_**b**_),^5^ as well as polymer-based triads bearing an NDI multiacceptor and a poly(carbazole) multidonor.^10^ In the meantime, Wenger and co-workers reported on two related molecular triads based on an extended tetrathiafulvalene (ExTTF) donor and NDI acceptors, that are connected differently via a xylyl- or a di(*iso*-propyl)phenyl linker and at different attachment positions on the acceptor (core vs periphery).^6^ The authors report remarkable long charge separation lifetimes but relatively low quantum yields of CS formation, which definite origin remains open to be fully investigated. In view of the absence of (time-resolved) emission data, the possibility of secondary electrochemical reactions of the ExTTF donors and, thus, the challenges to obtain redox potentials that resemble the mere Faradaic processes led us to deliberately omit these interesting assemblies in the following discussion. Instead, we will compare their findings and our independent interpretation afterward (vide infra).

First, the driving forces of the oxidative (Δ*G*_ox_) and the reductive (Δ*G*_red_) excited state quenching pathways can be estimated from the excited state energy (*E*_0–0_) of the sensitizer and the formal redox potentials (see Table 1 for redox potentials).^23^ Neglecting the Coulombic work term and assuming *E*_0–0_ ≈ *E*^em^, the driving forces for the initial charge separation can be calculated by eqs 1 and 2, respectively (A is acceptor, D is donor). Consequently, the driving force for secondary electron transfer steps can be calculated as well as the energy of the fully CS state (*E*_CS_) given by the potential difference between the acceptor and donor units.
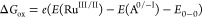
1
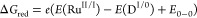
2

Table 3 summarizes the estimated driving forces including data of the related [Ru(dqp)_2_]^2+^-based triads. The molecular triads **I**_**a**_ and **I**_**b**_ reported by Kumar et al. contain a benzoquinone acceptor, which enables the oxidative quenching pathway by a driving force of −0.24 and −0.27 eV, respectively (Table 3, entries **I**_**a**_ and **I**_**b**_).^5^ The polymer-based triads that feature multiple NDI units (Table 3, entries **II**_**a**_ and **II**_**b**_) display considerably lower driving forces for the reductive pathway, because the reduction potential of an NDI unit is ca. 0.25 V lower compared to benzoquinone while the Ru^III/II^ redox-couple is stabilized by only 0.1 V due the different substitution pattern (Table 1, entries F, G vs entries D, E).^9,10^ Nevertheless, fast and efficient ^3^MLCT quenching has been identified at only mild driving forces (Δ*G*_ox_ = −0.09 eV) for the related dyad subunits,^21^ as well as for the polymer-based triads.^9,10^ The complementary reductive pathways for all reported triads are energetically uphill (Δ*G*_red_ > 0.22 eV) and, thus, are omitted as sizable quenching pathways as discussed elsewhere.^5,9,10^ Notably, the triads and tetrad studied in this work bearing a single NDI acceptor (Table 1, entries A to C, Table 3, entries **III**_**a**_, **III**_**b**_, and **III**_**c**_) revealed negligible driving forces (±0.00 eV) for oxidative quenching while reductive quenching remains endergonic. Beside the above driving force estimation, it is important to consider the effect of entropic contributions to electron transfer in the case of multiple (*n* > 1) redox centers. It has been long realized that the splitting between the first and the last redox process amounts to 2*RT*/*F* ln(*n*), where n denotes the number of independent redox centers.^24^ Omitting additional effects, a polymer with 20 units would thus lead to a formal splitting of 154 mV at room temperature. Hence, the strict comparison of triads containing single redox sites (“molecular”) or multiple redox sites (“polymer”) should be taken with care, i.e., in case of the polymer the formal half wave potential may be regarded as a robust estimate while the onset of the redox process may represent a more realistic value for a single electron transfer.

**Figure 5:**
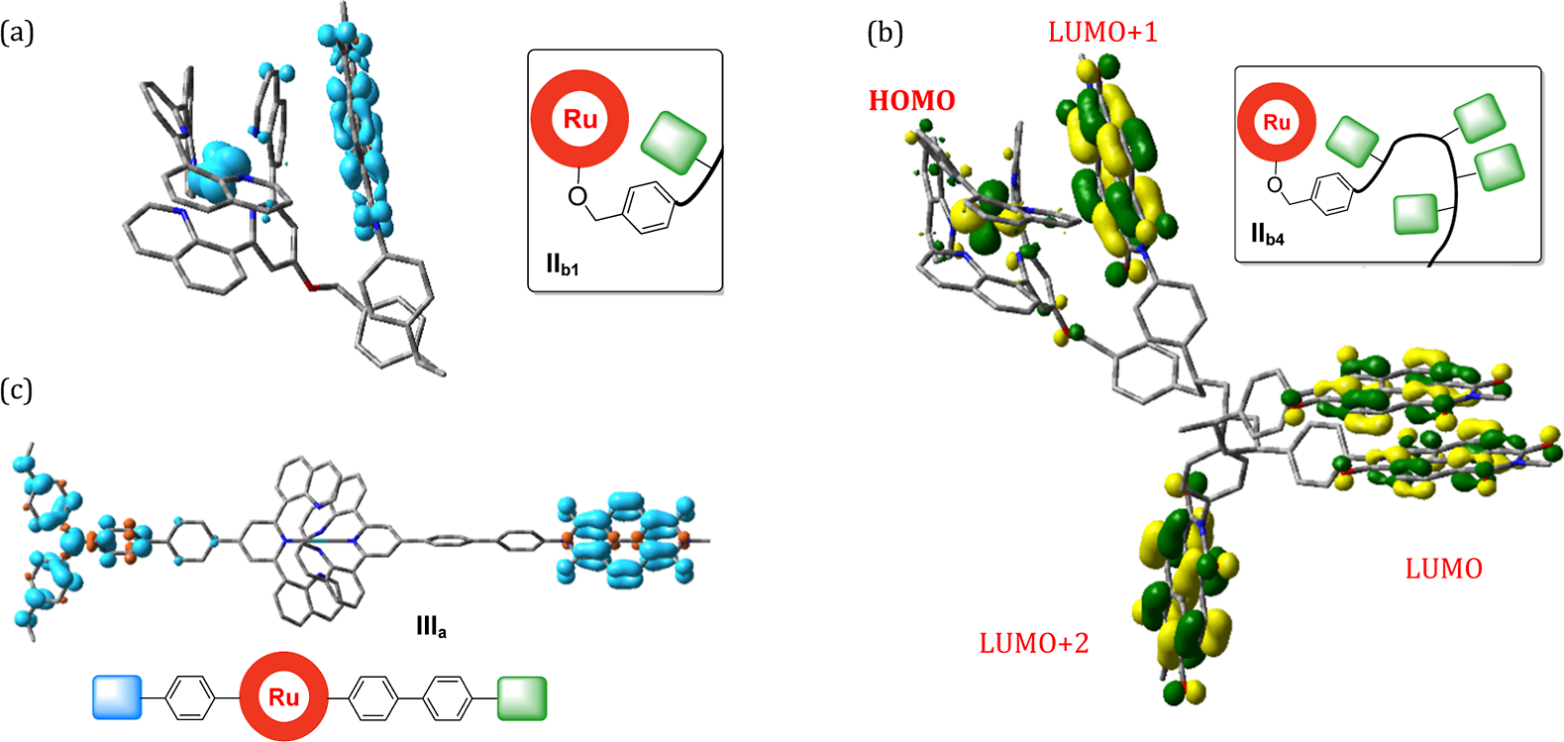
DFT-optimized structures of subsections of polymer triad **II**_**b**_ with one (**II**_**b1**_) or four (**II**_**b4**_) NDI units and the molecular triad **III**_**a**_: (a) spin-density plot (isovalue drawn at 0.004) of **II**_**b1**_ after oxidative quenching to form P^+^–A^–^ with a separation Ru···NDI distance of 5.5 Å and quinoline···NDI π-stacking distance of approximately 3.3 Å. (b) Frontier orbitals of a random conformation of **II**_**b4**_ in the order HOMO (Ru), LUMO (π-stacked NDI dimer), LUMO + 1 (π-stacked NDI···quinoline), and LUMO + 2 (peripheral NDI) (c) spin density plot for **III**_**a**_ (isovalue drawn at 0.004) of the lowest triplet state representing the CS state D^+^–P–A^–^. See Supporting Information, Chapter 11, for details.

Second, the utilization of polymer-based donors or acceptors is accompanied by conformational flexibility, which results in an ensemble of (unknown) orientations and mutual distances of the donor, photosensitizer and acceptor units. Scheme 4a illustrates the structurally well-defined linear molecular D–P–A triads (**I**_**a**_ and **III**_**a**_, top). The photophysical processes and electron transfer steps can be explained by Jablonski energy diagrams (Scheme 4b) on the basis of electrochemical and spectroscopic data (vide supra). In general, photon absorption leads to population of the ^1^MLCT state (gray), which undergoes intersystem crossing and relaxation to the ^3^MLCT state (red) featuring an inherently long excited state (emission) lifetime. In the presence of a suitable donor, reductive quenching leads to the D^+^–P^–^–A state (blue), alternatively and common to most triads, oxidative quenching leads to D–P^+^–A^–^ (green). The secondary electron transfer leads to the fully charge-separated state (D^+^–P–A^–^). In the case of **I**_**a**_, the energetics enable the oxidative quenching pathway with a charge recombination of 0.14 μs (Scheme 4b, left).^5^ Similarly, the polymer-based triad **II**_**b**_ featuring a poly(NDI) multiacceptor undergoes oxidative quenching. Figure 5a displays the DFT-optimized lowest triplet state of a P–A subunit (**II**_**b1**_) in which the NDI unit undergoes π-stacking with one of the quinoline units due to the conformational freedom of the linkage pattern. The spin localization reveals the charge separation onto the acceptor, in line with the proposed oxidative quenching pathway in the corresponding triads. Next, the effect of multiple NDI units was exemplarily investigated for structure **II**_**b4**_ with four NDI units in representative mutual orientations (Figure 5b). The inspection of the frontier molecular orbitals reveals that the extent of π-stacking determines the energetic ordering, i.e., the LUMO is delocalized between two stacked NDI units, followed by the LUMO + 1 on the NDI unit stacked with the quinoline (as in **II**_**b1**_) and finally the LUMO + 2 on the peripheral NDI unit. These results qualitatively suggest that multiple sites with different Ru-NDI distances, orientations and energy levels exist, which facilitates charge separation beyond the formal driving force and may also explain the slower biexponential charge recombination of 0.7 and 7.2 μs (Scheme 4b, middle). Next, the molecular triad **III**_**a**_ revealed the charge-separated state as the lowest triplet state (Figure 5c) in line with the transient absorption data. In addition, the larger charge separation distance of **III**_**a**_ vs **I**_**a**_ (25.6 Å vs 17.3 Å, Scheme 4a) parallels the CS lifetime enhancement by a factor of 2, leading to a numerical recombination damping factor of 0.25 Å^–1^—the typical range of electron transfer.^25^ Likewise, the CS lifetime (320 ns) is in the typical range reported for similar triad systems.^26^ Remarkably, neither the D–P nor the P–A dyads indicate efficient ^3^MLCT quenching based on the typical excited state lifetimes, in line with the estimated negligible driving force for oxidative quenching or even uphill for reductive quenching. Since all D–P–A assemblies in this study lead to the charge-separated state, we tentatively assign the charge separation as a branching pathway after excitation (Scheme 4b, right, black dashed arrow) based on the following argumentation: The molecular D–P–A triad (**III**_**a**_) reveals 91% emission quenching with respect to the P–A dyad, the CS formation was calculated as 90% vs ^3^MLCT formation, which in turn decays with the intrinsic ^3^MLCT lifetime (2.3 μs). The related polymer-based triad and tetrad showed higher residual steady state emission (ca. 40%), a much larger contribution of ^3^MLCT in the TA spectra but complex kinetics that precluded a reliable quantification at this stage.

**Scheme 4:**
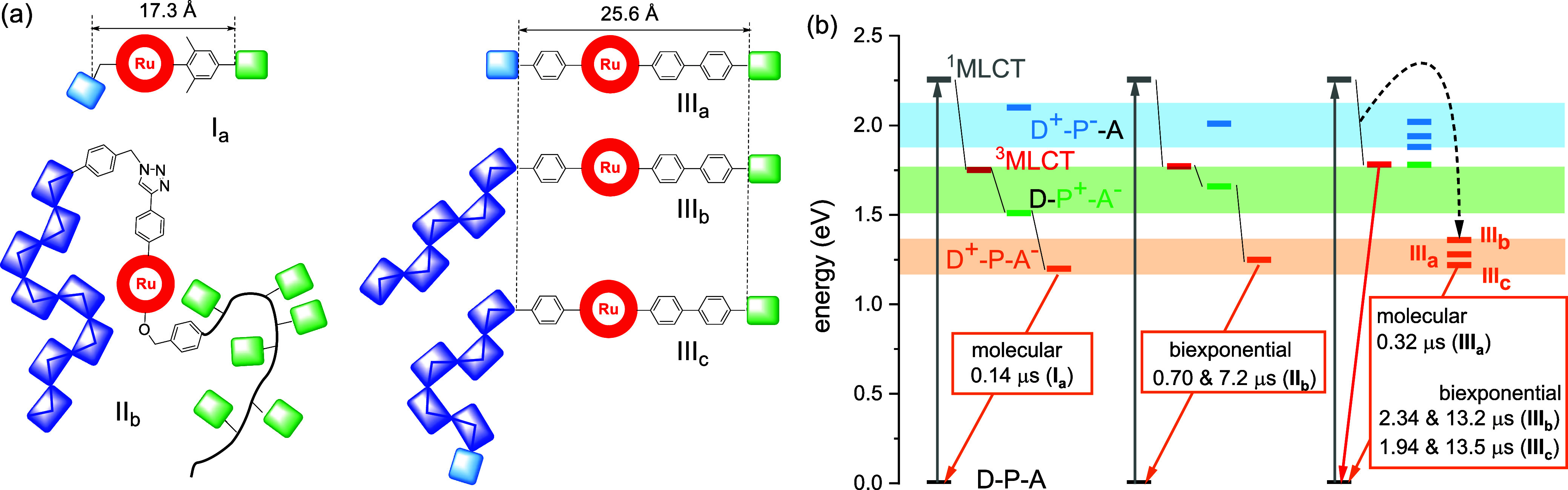
Energy Diagram and Schematic Representation of [Ru(dqp)_2_]^2+^-Based Triads and Tetrad (a) Molecular triads based on PTZ-donor and benzoquinone-acceptor (**I**_**a**_), polymer triads based on poly(naphthalene diimide) multiacceptor and poly(carbazole) multidonor (**II**_**b**_),^10^ and triads based on a single naphthalene diimide acceptor with a single TARA donor (**III**_**a**_), poly(carbazole) multidonor (**III**_**b**_) and a poly(carbazole) multidonor bearing one terminal TARA donor (**III**_**c**_). (b) Energy diagram showing population of ^1^MLCT state and intersystem crossing to the ^3^MLCT state (red). Quenching pathways via electron transfer to acceptor (green states, oxidative pathway), from donor (blue states, reductive pathway), emission including nonradiative decay (red arrow, to GS), or charge recombination (orange arrows, to GS).

Following the analysis of the molecular triad, the extent of CS formation should be up to that of emission quenching (ca. 60%). More importantly, the CS lifetimes are prolonged by one or 2 orders of magnitude, illustrating the success of charge separation despite the minimal formal driving forces. Notably, also the stored redox potential of the CS state was increased from 1.20 V (**I**_**a**_) to 1.28 V (**III**_**a**_) or formally 1.36 V (**III**_**b**_ and **III**_**c**_; Table 3). However, a more detailed analysis is beyond the stage of this work and will be reported in due course.

## Conclusion

This study reports the simple divergent preparation of a systematic series of dyads, triads and a tetrad for long-lived charge separation. The late-stage diversification by chemistry-on-the-complex enabled the modular attachment of different donor motifs at the identical position of the P–A subunit, in order to elucidate the precise impact on long-lived charge separation. Notably, the novel chemistry-on-the-complex approach circumvents typical challenges of conventional routes, i.e., the number of synthetic steps is reduced, side reactions and potentially instabilities of the donors and/or acceptors under the complexation conditions are prevented, and simplified purification procedures in all subsequent steps can be applied. In addition to the practical benefits to enable a modular design and efficient synthesis as well as analytical characterization, the prepared triads feature long-lived charge separation with a relatively high estimated energy content of up to 1.3 eV, considering the ^1^MLCT absorption shoulder up to 550 nm and the ^3^MLCT excited state energy of ca. 1.8 eV (vide supra). While the molecular reference triad falls into the typical range of submicrosecond charge separation lifetime, the polymer-based triad and tetrad lead to prolonged CS lifetimes by up to 2 orders of magnitude (up to 13.5 μs). However, it should be noted that the tetrad system—composed of the Ru unit, the NDI acceptor, the Carb_*n*_ primary donor and the TARA as a potential terminal donor–should be regarded a “functional triad” because no evidence of TARA oxidation was observed. The simultaneous qualitative decrease of CS formation efficiency, as recently also reported by Wenger and co-workers for related molecular triads,^6^ is ascribed to the marginal driving force.

The chemistry-on-the-complex approach combines a modular design, the facile preparation as well as the analysis of photoactive assemblies with preserved linkage patterns. Although the a priori selected redox units are not optimal, the iterative exploration of (multiple) electron donor and acceptor sites will become possible using the Ru sensitizer as a building block. In combination with the meanwhile reported fractionation of oligomers,^11^ the systematic investigation of the elementary electron transfer steps in the overall charge separation cascade can be envisioned. For example, each oligomer is expected to possess an intrinsic CS lifetime in analogy to the molecular triad, but to date only the “average” can be extracted by the biexponential fit. Hence, more work is necessary to assist the quest of a rational design and facile preparation of advanced photocatalytic or artificial photosynthetic (macro)molecular assemblies.

## Experimental Details

SEC was performed on a Shimadzu series 10 SCL-10A vp, equipped with degasser (DGU-14A), pump (LC-10AD vp), autosampler (SIL-10AD vp), overn (CTO-10AC vp), and RI- (RID-10A) and PDA-detector (SPD-10MA vp). The column used is a Phenomenex phenogel (100,000 Å/1000 Å, 10 μm particle size) and DMAc + 0.08 wt % NH_4_PF_6_ was used as eluent.

NMR spectra were recorded on a 400 MHz Bruker ACANCE III NMR spectrometer equipped with a BBFO probe in deuterated solvents at 300 K. Chemical shifts are reported in parts per million (ppm, δ scale) relative to the residual solvent signal.

ESI-ToF mass spectrometry was performed on a Bruker micrOTOF QII, or a timsTOF mass spectrometer by syringe injection.

MALDI-ToF mass spectrometry was performed on a Bruker ultraflex III or on a Bruker rapiflex using the dried droplet technique. DCTB and NaI were used as matrix compounds throughout.

Absorption spectroscopy was performed in 10 × 10 mm Hellma Analytics quartz glass cuvettes on a PerkinElmer Lambda 750 in dry solvents against blank samples.

Cyclic voltammetry and differential pulse voltammetry were performed using a Bio-Logic SAS VMP3 potentiostat, platin wire as counter electrode, Ag/AgNO_3_ as reference electrode, and glassy carbon as working electrode in CH_2_Cl_2_ containing 0.1 M tetrabutylammonium hexafluorophosphate.

For spectroelectro chemistry, the cyclic voltammetry setup was used in combination with a 1 × 10 mm Hellma Analytics quartz glass cuvette on a PerkinElmer Lambda 750 in dry solvents against blank samples. A platin mesh was used instead of a wire.

Emission spectroscopy was performed in 10 × 10 mm Hellma Analytics quartz glass cuvettes on a Jasco FP-8300 fluorescence spectrometer equipped with an ETC-815 Peltier thermostated single cell holder after calibration with a Jasco ESC-142 calibrated WI Light Source and Jasco VDK-140 Validaiton Kit 1 in dry solvents.

The nanosecond (ns) transient absorption and emission spectra were collected on a setup consisting of a Nd/YAG laser system (355 nm, pulse duration ca. 5 ns, repetition rate 10 Hz) pumping a Continuum Surelite OPO Plus, which is used to modulate the excitation pulse wavelength (500 nm). The probe light is provided by a 75 W xenon arc lamp. Spherical concave mirrors are used to focus the probe beam into the samples and then send the beam to the monochromator (Acton, Princeton Instruments). The probe light is detected by a Hamamatsu R928 photomultiplier. The signal is amplified and processed by a commercially available detection system (Pascher Instruments AB). Freshly prepared samples with an optical density (ca. 0.1 or 0.4) at the excitation wavelength (500 nm) were used. Oxygen-free solutions were prepared by freeze–pump–thaw cycles or prepared in a Ar-filled glove box. All measurements were performed in 1 cm path length fluorescence cuvettes. The spectrum at the excitation wavelength is omitted from the data analysis due to pump-scatter in this spectral range.

The theoretical calculations are based on density functional theory (DFT). All calculations were performed with the Gaussian16W program package (Version A.03).^27^ The hybrid functional B3LYP^28^ including the dispersion correction (keyword “EmpiricalDispersion = GD3”) have been selected in combination with the 6-31G* basis set for all atoms except Ru, which were described by an effective core potential and the associated orbitals (mwb). For all calculation, the solvent environment was modeled for acetonitrile using the implemented polarization continuum model.^29^ The geometries of the singlet ground state were first optimized (standard setting for “opt”, ultrafine integration grid “int = ultrafine”), which serve as the starting point for the optimization of the corresponding triplet states (T_1_). The true nature of the molecular triads TARA-Ph/Ph-Ph-NDI (**III**_**a**_) and PTZ-CH_2_/Xyl-BQ were confirmed by vibrational analysis revealing no imaginary frequencies. The graphical visualizations were generated by GaussView 6.0.16,^30^ i.e. isovalues were drawn at 0.04 (Kohn–Sham molecular orbitals) or 0.004 (spin-density calculations).

### Materials

All chemicals and solvents were purchased from abcr, Acros Organics, Alfa Aesar, Apollo Scientific, Fluka, fluorochem, Sigma-Aldrich, TCI, or VWR and used without further purification if not stated otherwise.

4-(4′-bromophenyl)-2,6-diquinoline-8′-diylpyridine,^31^*N*-(4′-iodophenyl)-*N*′-(2′-ethylhexyl)-naphthalene-1,4,5,8-tetracarboxylic acid diimide (NDI-Ph-I),^32^ 4-(4′-phenylboronic acid pinacol ester)-2,6-diquinoline-8′-diylpyridine (dqp-Ph-Bpin),^33^ 4-(4′-(4’’-(*N*-(2‴-ethylhexyl)NDI-*N*′-yl)phenyl)phenyl)-2,6-diquinoline-8′-diylpyridine (dqp-Ph-Ph-NDI),^33^ [(Br-Ph-dqp)Ru(MeCN)_3_]^2+^ (starting from [(iPrSPh)_2_(MeOH)RuCl_3_]),^34^ [(Br-Ph-Ph-dqp)Ru(dqp-Ph-Ph-NDI)]^2+^,^33^ [Ru(MeCN)_6_](BF_4_)_2_,^35^ 3,6-dibromo-*N*-(4-(2′-ethylhexyloxy)phenyl)carbazole (Br-Carb-Br, adopted procedure),^36^ SPhos Pd G3,^37^ and RuPhos Pd G3^37^ were prepared according to literature procedures.

Note that dicationic species carry two PF_6_^–^ anions which are not shown throughout for clarity.

#### Synthetic Procedures

##### 4-(4′-(*N*,*N*-Bis(4″-methoxyphenyl)anilin-4-yl)phenyl)-2,6-diquinoline-8′-diylpyridine (dqp-Ph-TARA) dqp-Ph-Bpin

(34.0 mg, 0.063 mmol), 4-iodo-*N*,*N*-bis(4′-methoxyphenyl)anilin (30.2 mg, 0.070 mmol), SPhos Pd G3 (8.4 mg, 0.011 mmol), SPhos (7.6 mg, 0.019 mmol), and potassium phosphate (108.8 mg, 0.512 mmol) were combined in a microwave vial and the atmosphere inside replaced with nitrogen. Dry toluene (2 mL) and H_2_O (0.1 mL) were added and the vial was placed in a preheated oil bath (50 °C) under stirring overnight. After cooling to room temperature, the reaction mixture was added to H_2_O (25 mL) and extracted with CH_2_Cl_2_ (2 × 25 mL). The combined organic phases were washed with brine (50 mL), dried over Na_2_SO_4_, filtered and the solvent was evaporated. The crude product was subjected to flash column chromatography (10 g SiOH-cartridge, CH_2_Cl_2_/MeOH 95:5) yielding the pure product (19 mg, 42%).

^1^H NMR (400 MHz, CDCl_3_, δ): = 9.01 (br s, 2H, quH), 8.43 (br s, 4H, pyH + quH), 8.33 (d, 2H, quH, *J* = 7.82 Hz), 7.96 (t, 4H, quH, *J* = 8.74 Hz), 7.75 (t, 2H, quH, *J* = 7.36 Hz), 7.69 (d, 2H, quH, *J* = 7.85 Hz), 7.57 (br s, 2H, quH), 7.47 (d, 2H, PhH, 8.63 Hz), 7.10 (d, 4H, PhH, *J* = 8.89 Hz), 6.99 (d, 2H, PhH, *J* = 8.62 Hz), 6.85 (d, 4H, PhH, *J* = 8.90 Hz), 3.81 (s, 6H, −OCH_3_) ppm.

^13^C{^1^H}-NMR (100 MHz, CDCl_3_): δ = 156.17 (s, 2C, PhC), 150.41 (s, 2C, quCH), 148.70 (s, 2C, PhC), 140.85 (s, 2C, PhC), 137.37 (s, 2C, quCH), 132.12 (s, 2C, quCH), 128.95 (s, 2C, quCH), 128.22 (s, 2C, quCH), 127.62 (s, 2C, PhCH), 127.05 (s, 2C, quCH), 126.91 (s, 4C, PhCH), 123.16 (s, 2C, pyCH), 121.64 (s, 2C, quCH), 120.57 (s, 2C, PhCH), 114.90 (s, 4C, PhCH), 55.65 (s, 2C, −OCH_3_) ppm.

HR-ESI-MS ([C_49_H_36_N_4_O_2_+H]^+^): calcd: 713.2911 *m*/*z*; found: 713.2909 *m*/*z*; error: 0.3 ppm.

##### 3-Boronic Acid Pinacol Ester-6-bromo-*N*-(4-(2′-ethylhexyloxy)phenyl)carbazole (Bpin-Carb-Br)

3,6-Dibromo-*N*-(4-(2′-ethylhexyloxy)phenyl)carbazole (7.209 g, 13.62 mmol) was dissolved in dry THF (135 mL) under a nitrogen atmosphere and cooled to −78 °C in a *i*PrOH/CO_2_ (s) cooling bath under stirring. *n*-Butyllithium (2 M in THF, 6.8 mL, 13.60 mmol) was added dropwise under vigorous stirring. After stirring at −78 °C for 40 min, 2-isopropoxy-4,4,5,5-tetramethyl-1,3,2-dioxaborolane (6.95 mL, 6.34 g, 34.07 mmol) was added in one portion. After further stirring for 30 min, the mixture was allowed to warm to room temperature and further stirred for 2.5 h. A saturated aqueous sodium hydrogen carbonate solution was added and the mixture was further stirred for 30 min. The mixture was diluted with H_2_O (300 mL), and extracted with CH_2_Cl_2_ (3 × 200 mL). The combined organic phases were dried over sodium sulfate, filtered, and the solvent was evaporated. The crude product was subjected to flash column chromatography (about 340 g SiOH, *n*-hexane/CH_2_Cl_2_ 60:40 to 50:50) to yield the pure product (4.24 g, 54%).

^1^H NMR (400 MHz, CDCl_3_, δ): = 8.59 (s, 1H, CarbH), 8.28 (d, 1H, CarbH, *J* = 1.77 Hz), 7.86 (d, 1H, CarbH, *J* = 8.33 Hz), 7.46 (dd, 1H, CarbH, *J* = 8.67 Hz, *J* = 1.89 Hz), 7.38 (d, 2H, PhH, *J* = 8.82 Hz), 7.29 (d, 1H, CarbH, *J* = 8.26 Hz), 7.18 (d, 1H, CarbH, *J* = 8.66 Hz), 7.10 (d, 2H, PhH, *J* = 8.82 Hz), 3.94 (d, 2H, −NCH_2_–, *J* = 5.67 Hz), 1.80 (m, 1H, −CH−), 1.62–1.33 (m, 8H, −CH_2_−), 1.40 (s, 12H, −CH_3_), 0.98 (t, 3H, −CH_3_, *J* = 7.48 Hz), 0.94 (t, 3H, −CH_3_, *J* = 7.10 Hz) ppm.

^13^C{^1^H}-APT-NMR (100 MHz, CDCl_3_): δ = 159.16 (s, 1C, PhC), 143.90 (s, 1C, CarbC), 140.37 (s, 1C, CarbC), 133.05 (s, 1C, CarbCH), 129.39 (s, 1C, PhC), 128.66 (s, 1C, CarbCH), 128.54 (s, 2C, PhCH), 128.10 (s, 1C, CarbCH), 125.16 (s, 1C, CarbC), 123.34 (s, 1C, CarbCH), 121.92 (s, 1C, CarbC), 115.84 (s, 2C, PhCH), 112.96 (s, 1C, CarbC), 111.40 (s, 1C, CarbCH), 109.47 (s, 1C, CarbCH), 83.85 (s, 2C, −OC(CH_3_)_2_−), 71.00 (s, 1C, −NCH_2_−), 39.57 (s, 1C, −CH−), 30.71 (s, 1C, −CH_2_−), 29.27 (s, 1C, −CH_2_−), 25.07 (s, 4C, −CH_3_), 24.05 (s, 1C, −CH_2_−), 23.22 (s, 1C, −CH_2_−), 14.26 (s, 1C, −CH_3_), 11.31 (s, 1C, −CH_3_) ppm.

HR-ESI-MS ([C_32_H_39_BBrNO_3_]^+^): calcd: 575.2201 *m*/*z*; found: 575.2206 *m*/*z*; error: 0.2 ppm.

##### α-Tolyl-ω-bromo-poly(*N*-(4-(2′-ethylhexyloxy)phenyl)carbazole-3,6-diyl) (Tol-Carb_*n*_-Br)

RuPhos Pd G3 (6.68 mg, 8.00 μmol), RuPhos (5.68 mg, 12.17 μmol), 4-iodotoluene (35.27 mg, 0.162 mmol), and potassium phosphate (255.36 mg, 1.203 mmol) were combined in a vial with a rubber septum under a nitrogen atmosphere. Toluene (2 mL, after three freeze–pump–thaw cycles) and H_2_O (0.3 mL, after three freeze–pump–thaw cycles) were added and the mixture placed in a preheated oil bath at 50 °C under vigorous stirring for 1 h. The solution was cooled to room temperature and a Bpin-Carb-Br-solution (140.80 mg, 0.244 mmol in 4 mL toluene (as above)) was added in one portion. After 229 h at room temperature, the mixture was added to H_2_O (20 mL), and the aqueous layer was extracted with CH_2_Cl_2_ (3 × 20 mL). The combined organic phases were dried over sodium sulfate, filtered, and the solvent was evaporated. The crude polymer was subjected to preparative SEC (Toyopearl HW40F, CH_2_Cl_2_/MeOH 95:5) to yield the purified polymer batch (96 mg).

Analytical SEC (CHCl_3_/*i*PrOH/NEt_3_ 94:2:4, against PS): *M̅*_n_ = 1700 g/mol, *M̅*_w_ = 2400 g/mol, *D̵* = 1.39.

End groups were assigned via MALDI-ToF MS (Figure S31).

##### α-(*N*,*N*-Di(4-methoxyphenyl)aniline-4-yl)T-ω-bromo-poly(*N*-(4-(2′-ethylhexyloxy)phenyl)carbazole-3,6-diyl) (TARA-Carb_*n*_-Br)

4-Iodo-*N*,*N*-di(4-methoxyphenyl)aniline (68.80 mg, 0.160 mmol), and potassium phosphate (258.19 mg, 1.216 mmol) were combined in a vial with a rubber septum under a nitrogen atmosphere. H_2_O (0.3 mL, after 3 freeze–pump–thaw cycles) and RuPhos Pd G3 (6.63 mg, 7.94 μmol) and RuPhos (5.55 mg, 11.89 μmol) in THF (2 mL, after 3 freeze–pump–thaw cycles) were added and the mixture placed in a preheated oil bath at 50 °C under vigorous stirring for 1 h. The solution was cooled to room temperature and a Bpin-Carb-Br-solution (139.08 mg, 0.241 mmol in 4 mL THF (as above)) was added in one portion. After 45 h at room temperature, the mixture was put on H_2_O (10 mL), and the aqueous layer was extracted with CH_2_Cl_2_ (3 × 10 mL). The organic phases were combined and the solvent was evaporated. The crude polymer was filtered through a silica bed (SiOH, CH_2_Cl_2_/MeOH 95:5) and subjected to preparative SEC (Toyopearl HW50F, CH_2_Cl_2_/MeOH 95:5) to yield the purified polymer batch.

Analytical SEC (CHCl_3_/*i*PrOH/NEt_3_ 94:2:4, against PS): *M̅*_n_ = 1800 g/mol, *M̅*_w_ = 2200 g/mol, *D̵* = 1.22.

End groups were assigned via MALDI-ToF MS (Figure S32).

##### [(TARA-Ph-dqp)Ru(dqp-Ph-Br)]^2+^

[(Br-Ph-dqp)Ru(CH_3_CN)_3_]^2+^ (182 mg, 0.182 mmol) and dqp-Ph-TARA (127 mg, 0.178 mmol) were combined in a microwave vial. After sealing with a septum, dry DMF (5 mL) was added, and the vial purged with a nitrogen flow for 10 min. The vial was placed in a preheated oil bath at 120 °C for 2 days. After cooling to room temperature, the mixture was drop wised in 100 mL stirred diethyl ether (previously cooled to −20 °C). CH_2_Cl_2_ (5 mL) was added to the vial and also drop wised into the diethyl ether under stirring. The precipitate was filtered off, washed with diethyl ether (5 × 50 mL), and after drying for 30 min under ambient conditions flushed from the frit with CH_2_Cl_2_. The solvent was evaporated and the crude product subjected to column chromatography (SiOH, CH_3_CN/H_2_O/KNO_3(aq.,sat.)_ 40:4:1). Product-containing fractions were combined, solvents evaporated and dissolved in CH_2_Cl_2_ (50 mL). The phase was washed with NaPF_6_ (aq., 5 g/L, 2 × 50 mL), then with H_2_O (50 mL). The solvent was evaporated and the crude product dissolved in CH_3_CN (3 mL). The solution was heated in an oil bath (80 °C) and toluene (30 mL) was added dropwise under stirring. The stirring was stopped and the mixture was slowly cooled to room temperature in the oil bath overnight. The precipitate was filtered, washed with toluene (2 × 25 mL, then 3 × 15 mL), then diethyl ether (5 × 15 mL). The precipitate was flushed from the frit with CH_2_Cl_2_ and the solvent evaporated yielding the product as a red solid (80 mg, 28%).

^1^H NMR (500 MHz, CD_2_Cl_2_, δ): = 8.10–8.04 (m, 10H, quH + pyH), 7.99 (s, 2H, pyH), 7.89 (d, 2H, PhH, *J* = 8.46 Hz), 7.83 (m, 4H, quH), 7.78 (d, 2H, PhH, *J* = 8.47 Hz), 7.74 (d, 2H, PhH, *J* = 8.63 Hz), 7.71–7.66 (m, 6H, quH + PhH), 7.54 (t, 4H, quH, *J* = 7.80 Hz), 7.50 (d, 2H, TARA-H, *J* = 8.80 Hz), 7.19–7–12 (m, 4H, quH), 7.09 (d, 4H, TARA-H, *J* = 8.95 Hz), 6.96 (d, 2H, TARA-H, *J* = 8.75 Hz), 6.87 (d, 4H, TARA-H, *J* = 8.95 Hz), 3.79 (s, 6H, −OCH_3_) ppm.

^13^C{^1^H}-APT-NMR (125 MHz, CD_2_Cl_2_): δ = 158.37 (s, 2C, quCH), 158, 22 (s, 2C, quCH), 157.33 (s, 2C, quC), 157,11 (s, 2C, TARA-C), 156.76 (s, 2C. TARA-C), 150.59 (s, 1C, PhC), 149.87 (s, 1C, PhC), 149.47 (s, 1C, PhC), 146.99 (s, 4C, quC), 143.37 (s, 1C, PhC), 140.74 (s, 2C, TARA-C), 138.36 (s, 2C, quCH), 138.34 (s, 2C, quCH), 135.27 (s, 1C, ArC), 133.70 (s, 2C, quCH), 133.63 (s, 2C, quCH), 133.41 (s, 1C, ArC), 133.05 (s, 2C, PhCH), 132.22 (s, 2C, ArC), 132.09 (s, 2C, ArC), 131.40 (s, 2C, quCH), 131.38 (s, 2C, quCH), 130.94 (s, 1C, ArC), 129.44 (s, 2C, PhCH), 128.06 (s, 2C, ArCH), 127.80 (s, 2C, ArCH), 127.69 (s, 2C, ArCH), 127.61 (s, 2C, ArCH), 127.48 (s, 2C, ArCH), 127.36 (s, 4C, TARA-CH), 127.16 (s, 2C, ArC), 127.13 (s, 2C, ArC), 125.92 (s, 2C, pyCH), 125.69 (s, 2C, pyCH), 125.43 (s, 1C, ArC), 122.82 (s, 2C, quCH), 122.67 (s, 2C, quCH), 120.26 (s, 2C, TARA-CH), 115.12 (s, 4C, TARA-CH), 55.83 (s, 2C, −OCH_3_), ppm.

HR-ESI-MS ([C_78_H_54_BrN_7_O_2_Ru]^2+^): calcd: 650.6277 *m*/*z*; found: 650.6279 *m*/*z*; error: 1.0 ppm.

##### [Ru(dqp-Ph-Br)_2_][PF_6_]_2_

dqp-Ph-Br (306 mg, 0.63 mmol) and [Ru(CH_3_CN)_6_](BF_4_)_2_ (253 mg, 85%, 0.35 mmol) were combined in a vial with ethylene glycol (16 mL). The vial was sealed and heated to 140 °C under stirring for 22 h. The cooled red solution was put on H_2_O containing NH_4_PF_6_ (150 mL) and extracted with CH_2_Cl_2_ (100 mL, then 3 × 40 mL). The solvent was removed under reduced pressure and the crude product subjected to column chromatography (SiOH, CH_3_CN/H_2_O/KNO_3(aq.,sat.)_ 40:4:1). Product-containing fractions were combined, solvents evaporated under reduced pressure, and put on H_2_O containing NH_4_PF_6_ (120 mL). The mixture was extracted with CH_2_Cl_2_ (120 mL, then 2 × 35 mL) and the combined organic layers washed with H_2_O. The solvent was evaporated, and the mixture subjected to flash column chromatography (Diol-SiOH, CH_2_Cl_2_/MeOH 95:5) to yield the pure product (65 mg, 17%). ^1^H NMR (600 MHz, CD_3_CN, δ): = 8.13 (dd, 4H, quH, *J* = 5.10 Hz, *J* = 1.26 Hz), 8.10 (s, 4H, pyH), 8.09 (dd, 4H, quH, *J* = 8.10 Hz, *J* = 1.20 Hz), 7.89 (dd, 4H, quH, *J* = 7.35 Hz, *J* = 0.87 Hz), 7.87 (d, 4H, PhH, *J* = 8.58 Hz), 7.77 (d, 4H, PhH, *J* = 8.58 Hz), 7.71 (dd, 4H, quH, *J* = 8.22 Hz, *J* = 0.78 Hz), 7.49 (“t”, 4H, quH, *J* = 7.80 Hz), 7.08 (dd, 4H, quH, *J* = 8.07 Hz, *J* = 5.13 Hz) ppm. ^13^C{^1^H}-NMR (150 MHz, CD_3_CN): δ = 159.48 (s, 4C, quCH), 158.08 (s, 4C, quC), 149.36 (s, 2C, PhC), 147.53 (s, 4C, quC), 138.53 (s, 4C, quCH), 136.15 (s, 2C, pyC), 134.46 (s, 4C, quCH), 133.39 (s, 4C, PhCH), 132.74 (s, 4C, pyC), 131.60 (s, 4C, quCH), 130.33 (s, 4C, PhCH), 127.80 (s, 4C, quCH), 127.53 (s, 4C, quC), 126.17 (s, 4C, pyCH), 125.32 (s, 2C, PhC), 123.04 (s, 4C, quCH) ppm.

##### [(TARA-Ph-dqp)Ru(dqp-Ph-Bpin)][PF_6_]_2_

[(TARA-Ph-dqp)Ru(dqp-Ph-Br)][PF_6_]_2_ (39.60 mg, 0.025 mmol), bis(pinakolato)diboron (14.01 mg, 0.055 mmol), Pd(dppf)Cl_2_ (2.96 mg, 0.004 mmol), and potassium acetate (7.16 mg, 0.073 mmol) were combined in a microwave vial. The vial was sealed and the atmosphere inside was replaced with nitrogen and dry DMF (1.5 mL) was added. The vial was placed in a preheated oil bath at 80 °C overnight. After cooling to room temperature, the mixture was put on NaPF_6_ (aq., 5 g/L, 100 mL) and extracted with CH_2_Cl_2_ (100 mL). The organic phase was washed with H_2_O (100 mL) and the solvent was evaporated under reduced pressure. The crude product was dissolved in CH_2_Cl_2_ (2.5 mL) and drop wised in cooled diethyl ether (25 mL). The precipitate was filtered, washed with diethyl ether (4 × 10 mL), and flushed from the frit with CH_2_Cl_2_. The solvent was evaporated to yield the product (39 mg, 96%).

^1^H NMR (400 MHz, CD_2_Cl_2_, δ): = 8.12–8.03 (m, 12H, quH + pyH), 7.98 (d, 2H, PhH, *J* = 8.04 Hz), 7.90 (d, 2H, PhH, *J* = 8.20 Hz), 7.88–7.80 (m, 6H, quH + PhH), 7.78 (d, 2H, PhH, *J* = 8.28 Hz), 7.70 (d, 4H, quH, *J* = 7.96 Hz), 7.55 (t, 4H, quH, *J* = 7.20 Hz), 7.51 (d, 2H, TARA-H, *J* = 8.76 Hz), 7.16 (m, 4H, quH), 7.09 (d, 4H, TARA-H, *J* = 8.92 Hz), 6.96 (d, 2H, TARA-H, *J* = 8.72 Hz), 6.87 (d, 4H, TARA-H, *J* = 8.93 Hz), 3.80 (s, 6H, −OCH_3_), 1.37 (s, 12H, −CCH_3_) ppm.

^13^C{^1^H}-APT-NMR (100 MHz, CD_2_Cl_2_): δ = 158.33 (s, 2C, quCH), 158.26 (s, 2C, quCH), 157.29 (s, 2C, quC), 157.10 (s, 2C, qu-C), 156.77 (s, 2C, TARA-C), 150.81 (s, 1C, PhC), 150.65 (s, 1C, PhC), 149.48 (s, 1C, TARA-C), 147.02 (s, 4C, quC), 143.38 (s, 1C, PhC), 140.77 (s, 2C, TARA-C), 138.39 (s, 2C, pyCH), 136.11 (s, 2C, PhCH), 133.73 (s, 1C, pyC), 133.50 (s, 4C, quCH), 132.24 (s, 2C, quC), 132.16 (s, 2C, quC), 131.46 (s, 2C, quCH), 131.41 (s, 2C, quCH), 131.01 (s, 1C, TARA-C), 128.09 (s, ArCH), 127.82 (s, ArCH), 127.65 (br s, 4C, quCH), 127.50 (s, ArCH), 127.36 (s, 4C, TARA-CH), 127.18 (s, ArC), 126.87 (s, 4C, quCH), 126.13 (s, ArCH), 125.75 (s, 2C, pyCH), 122.73 (s, 4C, quCH), 120.30 (s, 2C, TARA-CH), 115.14 (s, 4C, TARA-CH), 84.62 (s, 2C, −BOC−), 55.84 (s, 2C, −OCH_3_), 25.08 (s, 4C, –C(CH_3_)_2_) ppm.

HR-ESI-MS ([C_84_H_66_BrN_7_O_4_Ru]^2+^): calcd: 674.7151 *m*/*z*; found: 674.7157 *m*/*z*; error: 1.5 ppm.

##### [(Bpin-Ph-dqp)Ru(dqp-Ph-Ph-NDI)][PF_6_]_2_

[(Br-Ph-dqp)Ru(dqp-Ph-Ph-NDI)][PF_6_]_2_ (29.99 mg, 0.017 mmol), bis(pinakolato)diboron (13.75 mg, 0.054 mmol), Pd(dppf)Cl_2_ (1.62 mg, 0.002 mmol), and potassium acetate (5.57 mg, 0.057 mmol) were combined in a microwave vial. The vial was sealed and put under reduced pressure for 1 h before the atmosphere was replaced by nitrogen. Dry DMF (1 mL) was added and the vial placed in a preheated oil bath at 80 °C overnight. After cooling to room temperature, the mixture was put on KPF_6_ (aq., 5 g/L, 50 mL) and extracted with CH_2_Cl_2_/MeOH (95:5, 50 mL). The organic phase was washed with H_2_O (50 mL) and the solvent removed under reduced pressure. The crude product was dissolved in CH_3_CN (2 mL) and drop wised in stirred diethyl ether (20 mL). The precipitate was filtered off, washed with diethyl ether (3 × 20 mL), and flushed from the frit with CH_2_Cl_2_. The solvent was evaporated yielding the product (30 mg, 97%).

^1^H NMR (500 MHz, CD_2_Cl_2_, δ): = 8.77 (m, 4H, NDIH), 8.13–8.03 (m, 12H, ArH), 8.01–7.93 (m, 4H, ArH), 7.91–7.79 (m, 10H, ArH), 7.73–7.65 (m, 4H, ArH), 7.59–7.50 (m, 4H, ArH), 7.48–7.42 (m, 2H, ArH), 7.16 (m, 4H, quH), 4.14 (m, 2H, −NCH_2_−), 1.95 (m, 1H, −CH−), 1.48–1.25 (m, 8H, −CH_2_−), 1.36 (s, 12H, −CH_3_), 0.95 (t, 3H, −CH_3_), 0.90 (t, 3H, −CH_3_) ppm. Signals at 7.75 (d, 0.6H) and 7.39 (d, 0.6H) ppm are remaining impurities fitting to a 1,4-substituted benzene.

^13^C{^1^H}-APT-NMR (125 MHz, CD_2_Cl_2_) δ = 163.50 (s, 4C, −C(O)N−), 158.41 (s, 2C, ArCH), 158.26 (s, 2C, ArCH), 157.29 (s, ArC), 157.24 (s, ArC), 157.18 (s, ArC), 150.78 (s, ArC), 150.49 (s, ArC), 150.34 (s, ArC), 147.03 (s, ArC), 146.99 (s, ArC), 142.57 (s, ArC), 141.31 (s, ArC), 140.86 (s, ArC), 140.79 (s, ArC), 138.42 (s, ArC), 138.37 (s, 4C, ArCH), 136.10 (s, 4C, ArCH), 135.49 (s, ArC), 135.36 (s, ArC), 134.94 (s, ArC), 134.88 (s, ArC), 133.57 (s, 2C, ArCH), 133.49 (s, 2C, ArCH), 132.20 (s, ArC), 132.15 (s, ArC), 131.55 (s, 2C, ArCH), 131.51 (s, ArCH), 131.47 (s, 2C, ArCH), 131.43 (s, 2C, ArCH), 131.27 (s, 2C, ArCH), 129.65 (s, 2C, ArCH), 129.51 (s, ArCH), 129.14 (s, ArCH), 128.69 (s, 2C, ArCH), 128.46 (s, 2C, ArCH), 128.39 (s, ArCH), 128.36 (s, 2C, ArCH), 128.26 (s, ArCH), 128.18 (s, ArCH), 127.69 (s, 2C, ArCH), 127.62 (s, 2C, ArCH), 127.51 (s, ArC), 127.47 (s, ArC), 127.44 (s, ArC), 127.29 (s, ArC), 127.18 (s, ArC), 127.16 (s, ArC), 126.95 (s, ArC), 126.84 (s, 4C, ArCH), 126.12 (s, 2C, ArCH), 126.03 (s, 2C, ArCH), 125.83 (s, ArCH), 122.82 (s, 2C, quCH), 122.72 (s, 2C, quCH), 84.62 (s, 2C, −OC(CH_3_)), 44.85 (s, 1C, −NCH_2_−), 38.35 (s, 1C, −CH−), 31.04 (s, 1C, −CH_2_−), 29.01 (s, 1C, −CH_2_−), 25.08 (d, 2C, −OCCH_3_), 25.06 (d, 2C, −OCCH_3_), 24.37 (s, 1C, −CH_2_−), 23.43 (s, 1C, −CH_2_−), 14.24 (s, 1C, −CH_3_), 10.74 (s, 1C, −CH_3_) ppm.

HR-ESI-MS ([C_92_H_73_BN_8_O_6_Ru]^2+^): calcd: 749.2389 *m*/*z*; found: 749.2382 *m*/*z*; error: 3.3 ppm.

##### [(TARA-Ph-dqp)Ru(dqp-Ph-Ph-NDI)][PF_6_]_2_

NDI-Ph-I (10.41 mg, 0.018 mmol), RuPhos Pd G3 (14.63 mg, 0.018 mmol), RuPhos (7.81 mg, 0.017 mmol), and potassium phosphate (18.09 mg, 0.085 mmol) were combined in a microwave vial, the atmosphere was replaced by nitrogen, and H_2_O (0.15 mL) and THF (1 mL) were added. The vial was placed in a preheated oil bath at 50 °C for 1 h. Meanwhile, [(TARA-Ph-dqp)Ru(dqp-Ph-Bpin)][PF_6_]_2_ (14.94 mg, 0.009 mmol) was placed in a microwave vial, the atmosphere was replaced with nitrogen and THF (2 mL) was added. The solution was added to the heated mixture and the resulting mixture stirred at 50 °C overnight. The conversion was monitored by SEC. After cooling to room temperature, the mixture was put on NaPF_6_ (aq., 5 g/L, 100 mL) and the aqueous layer was extracted with CH_2_Cl_2_, then CH_2_Cl_2_/MeOH (90:10). The combined organic phases were washed with H_2_O (100 mL), and the aqueous layer extracted with CH_2_Cl_2_/MeOH (90:10). The organic phases were combined, and the solvent was evaporated. The crude product was dissolved in CH_3_CN (2 mL) and precipitated in diethyl ether (20 mL). The precipitate was filtered off and washed with diethyl ether (3 × 20 mL). The precipitate was flushed from the frit with CH_2_Cl_2_ and the solvent evaporated. After an ineffective crystallization attempt (drop wising toluene (20 mL) in a stirred solution of crude product in CH_3_CN (2 mL)), the crude product was subjected to FCC (10 g SiOH-cartridge, CH_2_Cl_2_/MeOH 96:4) yielding the pure product (9 mg, 50%).

^1^H NMR (400 MHz, CD_2_Cl_2_, δ): = 8.79 (m, 4H, NDI-H), 8.06–8.15 (m, 12H, quH + pyH), 7.98 (d, 2H, PhH, *J* = 8.44 Hz), 7.94 (d, 2H, PhH, *J* = 8.42 Hz), 7.92–7.81 (m, 8H, quH + PhH), 7.77 (d, 2H, PhH, *J* = 8.45 Hz), 7.72 (d, 4H, quH, *J* = 8.20 Hz), 7.56 (m, 4H, quH), 7.51 (d, 2H, TARA-H, *J* = 8.76 Hz), 7.46 (d, 2H, PhH, *J* = 8.42 Hz), 7.21–7.14 (m, 4H, quH), 7.10 (d, 4H, TARA-H, *J* = 8.95 Hz), 6.97 (d, 2H, TARA-H, *J* = 8.72 Hz), 6.87 (d, 4H, TARA-H, *J* = 8.96 Hz), 4.14 (m, 2H, −NCH_2_), 3.80 (s, 6H, −OCH_3_), 1.95 (m, 1H, −CH−), 1.47–1.28 (m, 8H, −CH_2_−), 0.95 (t, 3H, −CH_3_, *J* = 7.43 Hz), 0.90 (t, 3H, −CH_3_, *J* = 7.11 Hz) ppm.

^13^C{^1^H}-APT-NMR (100 MHz, CD_2_Cl_2_) δ = 163.54 (s, 2C, NDIC), 163.52 (s, 2C, NDIC), 158.37 (s, 2C, quCH), 158.32 (s, 2C, quCH), 157.24 (s, 1C, PhC), 157.10 (s, 2C, pyC), 156.78 (s, 2C, TARA-C), 150.63 (s, 1C, PhC), 150.52 (s, 1C, PhC) 149.53 (s, 1C, TARA-C), 147.03 (s, 4C, quC), 143.43 (s, 1C, PhC), 142.71 (s, 1C, PhC), 140.82 (s, 1C, PhC), 140.71 (s, 2C, TARA-C), 138.36 (s, 4C, quCH), 135.44 (s, 2C, pyC), 135.40 (s, 2C, pyC), 133.61 (s, 1C, TARA-C), 133.49 (s, 2C, quCH), 133.42 (s, 2C, quCH), 132.27 (s, 2C, quC), 132.22 (s, 2C, quC), 131.58 (s, 2C, NDICH), 131.44 (s, 2C, quCH), 131.42 (s, 2C, quCH), 131.29 (s, 2C, NDICH), 130.78 (s, 1C, PhC), 129.67 (s, 2C, PhCH), 128.78 (s, 2C, PhCH), 128.30 (s, 2C, PhCH), 127.99 (s, 2C, PhCH), 127.78 (s, 2C, PhCH), 127.65 (s, 4C, quCH), 127.54 (s, 4C, NDIC), 127.49 (s,2C, TARA-CH), 127.41 (s, 4C, TARA-CH), 127.31 (s, 1C, NDIC), 127.19 (s, 4C, quC), 126.94 (s, 1C, NDIC), 126.05 (s, 2C, pyCH), 125.74 (s, 2C, pyCH), 122.77 (s, 2C, quCH), 122.74 (s, 2C, quCH), 120.20 (s, 2C, TARA-CH), 115.11 (s, 4C, TARA-CH), 55.83 (s, 2C, −OCH_3_), 44.85 (s, 1C, −CH_2_−), 38.35 (s, 1C, −CH−), 31.04 (s, 1C, −CH_2_−), 29.01 (s, 1C, −CH_2_−), 24.36 (s, 1C, −CH_2_−), 23.45 (s, 1C, −CH_2_−), 14.25 (s, 1C, −CH_3_), 10.74 (s, 1C, −CH_3_) ppm.

HR-ESI-MS ([C_106_H_79_N_9_O_6_Ru]^2+^): calcd: 837.7593 *m*/*z*; found: 837.7580 *m*/*z*; error: 3.3 ppm.

##### [(Tol-Carb_*n*_-Ph-dqp)Ru(dqp-Ph-Ph-NDI)][PF_6_]_2_

Tol-Carb_*n*_-Br (12.59 mg, 0.007 mmol), RuPhos Pd G3 (6.72 mg, 0.008 mmol), RuPhos (6.04 mg, 0.013 mmol), and K_3_PO_4_ (8.69 mg, 0.041 mmol) were combined in a microwave vial, the atmosphere was replaced by nitrogen, and H_2_O (0.15 mL) and THF (1 mL) were added. The vial was placed in a preheated oil bath at 50 °C under stirring for 1.25 h. Meanwhile, [(Bpin-Ph-dqp)Ru(dqp-Ph-Ph-NDI)][PF_6_]_2_ (7.60 mg, 0.004 mmol) was dissolved in THF (2 mL) under a nitrogen atmosphere. The resulting solution was added subsequent to the stirred mixture at 50 °C. The resulting mixture was stirred at 50 °C for 20.5 h, cooled to room temperature, and put on KPF_6_ (aq., 5 g/L, 50 mL). The aqueous phase was extracted with CH_2_Cl_2_ (50 mL), then with CH_2_Cl_2_/MeOH 95:5 (2 × 20 mL). The combined organic phases were washed with H_2_O (50 mL), and the aqueous layer was extracted with CH_2_Cl_2_/MeOH 95:5 (2 × 20 mL). The organic phases were combined and the solvent was evaporated under reduced pressure. The crude product was subjected to FCC (40 g SiOH-cartridge, CH_2_Cl_2_/MeOH 98:2 to 90:10) yielding the product.

The efficient reaction was assigned via ^1^H- and ^1^H-DOSY-NMR, MALDI-ToF-MS, as well as SEC (DMAc + 0.08 wt % NH_4_PF_6_) (see Figures S19, S20, S33, and S45).

##### [(TARA-Carb_*n*_-Ph-dqp)Ru(dqp-Ph-Ph-NDI)][PF_6_]_2_

TARA-Carb_*n*_-Br (15.20 mg, 0.008 mmol), RuPhos Pd G3 (6.96 mg, 0.008 mmol), RuPhos (6.03 mg, 0.013 mmol), and K_3_PO_4_ (9.49 mg, 0.045 mmol) were combined in a microwave vial, the atmosphere was replaced by nitrogen, and H_2_O (0.15 mL) and THF (1 mL) were added. The vial was placed in a preheated oil bath at 50 °C under stirring for 1.25 h. Meanwhile, [(Bpin-Ph-dqp)Ru(dqp-Ph-Ph-NDI)][PF_6_]_2_ (7.60 mg, 0.004 mmol) was dissolved in THF (2 mL) under a nitrogen atmosphere. The resulting solution was added subsequent to the stirred mixture at 50 °C. The resulting mixture was stirred at 50 °C for 20.5 h, cooled to room temperature, and put on KPF_6_ (aq., 5 g/L, 50 mL). The aqueous phase was extracted with CH_2_Cl_2_ (50 mL), then with CH_2_Cl_2_/MeOH 95:5 (2 × 20 mL). The combined organic phases were washed with H_2_O (50 mL), and the aqueous layer was extracted with CH_2_Cl_2_/MeOH 95:5 (2 × 20 mL). The organic phases were combined and the solvent was evaporated under reduced pressure. The crude product was subjected to FCC (40 g SiOH-cartridge, CH_2_Cl_2_/MeOH 98:2 to 90:10) yielding the product.

Note: The efficient reaction was assigned via ^1^H- and ^1^H-DOSY-NMR, MALDI-ToF-MS, as well as SEC (DMAc + 0.08 wt % NH_4_PF_6_) (see Figures S21, S22, S34and S45).

## Supporting Information Available

The Supporting Information is available free of charge. (PDF). 3D-coordinates according to DFT-optimizations (ground state) of I_a_ (CIF), II_b1_ (CIF), II_b4_ (CIF), III_a_ (CIF). The Supporting Information is available free of charge at https://pubs.acs.org/doi/10.1021/acs.inorgchem.4c03693.

Full ^1^H-, and ^13^C NMR spectra, HR-ESI-ToF- and MALDI-ToF-mass spectra, size exclusion chromatograms, (spectro)electrochemical data, steady-state absorption and emission spectra, transient absorption and emission spectra, DFT calculations (PDF)

## Abbreviations

^1^MLCT: singlet metal to ligand charge transfer state
^3^MLCT: triplet metal to ligand charge transfer state
ΔO.D.: change in optical density
Φ_em_: relative emission quantum yield
τ_TA_: half-life time according to transient absorption data
A: acceptor
a.u.: arbitrary unit
Bpin: boronic acid pinacol ester
BQ: benzoquinone-2-yl
Carb_*n*_: poly(*N*-(4′-(2″-ethylhexyloxy)phenyl)carbazole-3,6-diyl)
Carb’_*n*_: poly(*N*-(2′-ethylhexyl)carbazole-3,6-diyl)
CH_2_Cl_2_: dichloromethane
CH_3_CN: acetonitrile
cNDI: core-substituted NDI^6^
CS: charge-separated
CV: cyclic voltammetry
D: donor
DCTB: *trans*-2-[3-(4-*tert*-butylphenyl)-2-methyl-2-propenylidene]malononitrile
DPV: differential pulse voltammetry
NDI: *N*-(2′-ethylhexyl)naphthalene-1,4,5,8-tetracarboxylic acid-1:8,4:5-diimide-*N*′-yl
P: photosensitizer
PTZ: phenothiazine-*N*-methylen-yl
[Ru(dqp)_2_]^2+^: ruthenium(II) bis(2,6-di(quinolin-8-yl)pyridine)
TA: transient absorption spectroscopy
TARA: *N*,*N*-di(4-methoxyphenyl)aniline-4-yl
TARA’_*n*_: poly(4-(*N*,*N*-di(4-butylphenyl)amino)styrene)
TBAPF_6_: tetra-*n*-butylammonium hexafluorophosphate
trz: 1,2,3-triazole-1,4-diyl
xyl: 1,3-xylene-2,5-diyl
