# Supplementary material for: From Molecular to Polymeric Donors: Prolonged Charge Separation in Modular Photoredox-Active Ru(II) Polypyridyl-Type Triads
Source: Inorg Chem. 2024 Nov 25;63(49):23233–47. doi: 10.1021/acs.inorgchem.4c03693 (PMC11632771; doi:10.1021/acs.inorgchem.4c03693)
Supplement: Supplementary file 1 — ic4c03693_si_001.pdf [file ic4c03693_si_001.pdf]

Supporting Information

From molecular to polymeric donors: Prolonged charge separation in modular photoredox-active Ru(II) polypyridyl-type triads

Alexander Kleine,[a] Charlotte Mankel,[a] Andrea Hainthaler,[b] Maria Wächtler,[c, d, e] Benjamin Dietzek-Ivanšić,[b, c, d] Ulrich S. Schubert,[,a,c] and Michael Jäger[*,a,c]*

[a] Laboratory of Organic and Macromolecular Chemistry (IOMC), Friedrich Schiller University Jena, 07743 Jena, Germany.

[b] Institute for Physical Chemistry (IPC), Friedrich Schiller University Jena, 07743 Jena, Germany.

[c] Center for Energy and Environmental Chemistry Jena (CEEC Jena), Friedrich Schiller University Jena, 07743 Jena, Germany.

[d] Research Department Functional Interfaces, Leibniz Institute of Photonic Technology Jena, Albert-Einstein-Straße 9, 07745 Jena, Germany.

[e] Chemistry Department and State Research Center OPTIMAS, RPTU Kaiserslautern-Landau, 67663 Kaiserslautern, Germany.

Corresponding authors:

Ulrich S. Schubert: ulrich.schubert@uni-jena.de

Michael Jäger: michael.jager.iomc@uni-jena.de

1 Contents

2 NMR data 3

2.1 Spectra 3

2.2 Comparsion 15

3 Mass spectrometry 16

3.1 ESI-ToF MS data. 16

3.2 MALDI-ToF MS data 20

3.3 Comparison 22

4 Size exclusion chromatography 23

5 Electrochemistry 31

6 Spectroelectrochemistry 32

7 Transient absorption and emission spectroscopy 33

7.1 Reference dyads 33

7.2 Molecular triad (III_a) 34

7.3 Polymer-based triad (III_b) 35

7.4 Polymer-based tetrad (III_c) 37

8 DFT calculations 39

2 NMR data

2.1 Spectra

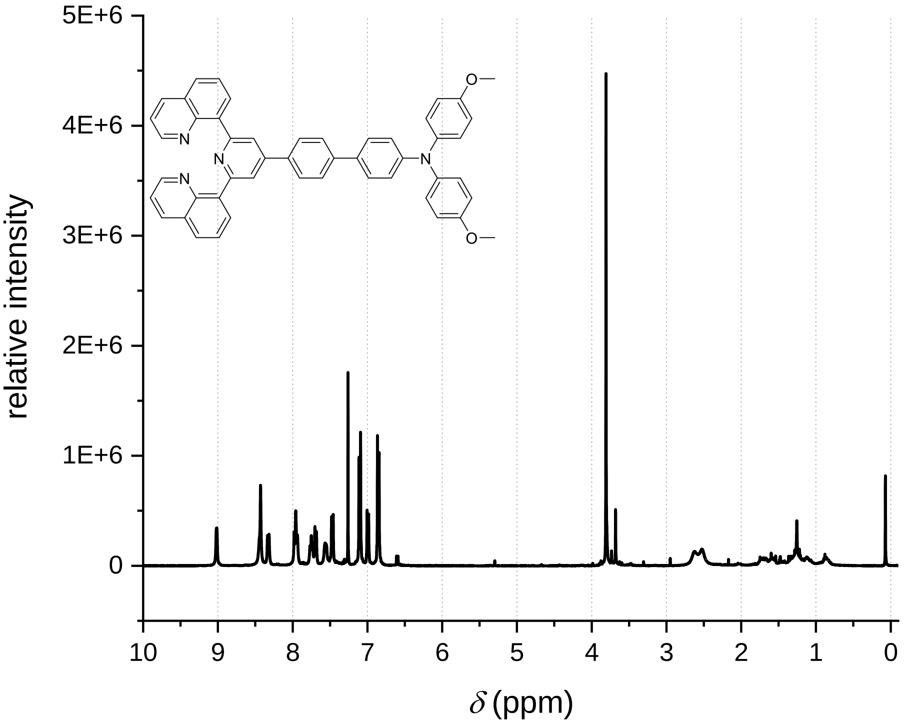

Figure S1. ^{1}H-NMR spectrum of dqp-Ph-TARA (400 MHz, $CDCl_3$).

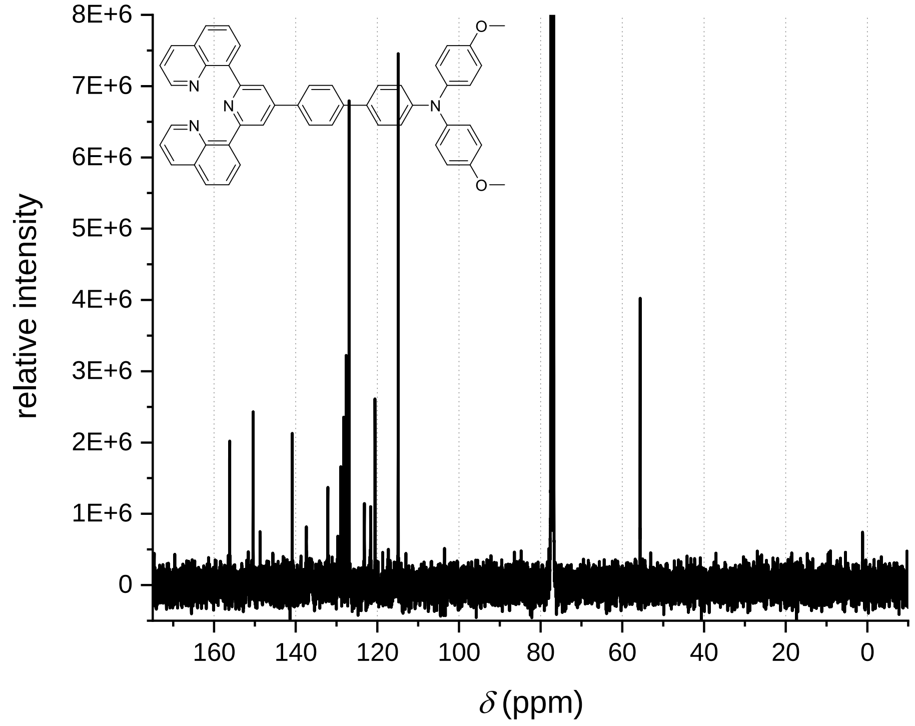

Figure S2. $^{13}C\{^{1}H\}$-NMR spectrum of dqp-Ph-TARA (100 MHz, $CDCl_3$).

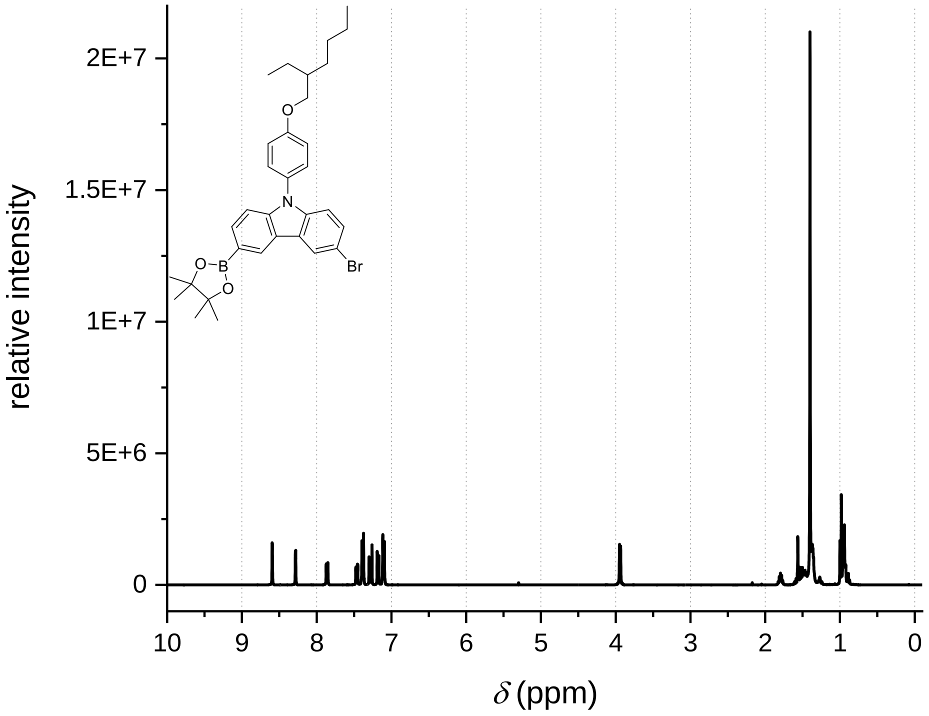

Figure S3. ^{1}H-NMR spectrum of Bpin-Carb-Br (400 MHz, $CDCl_3$).

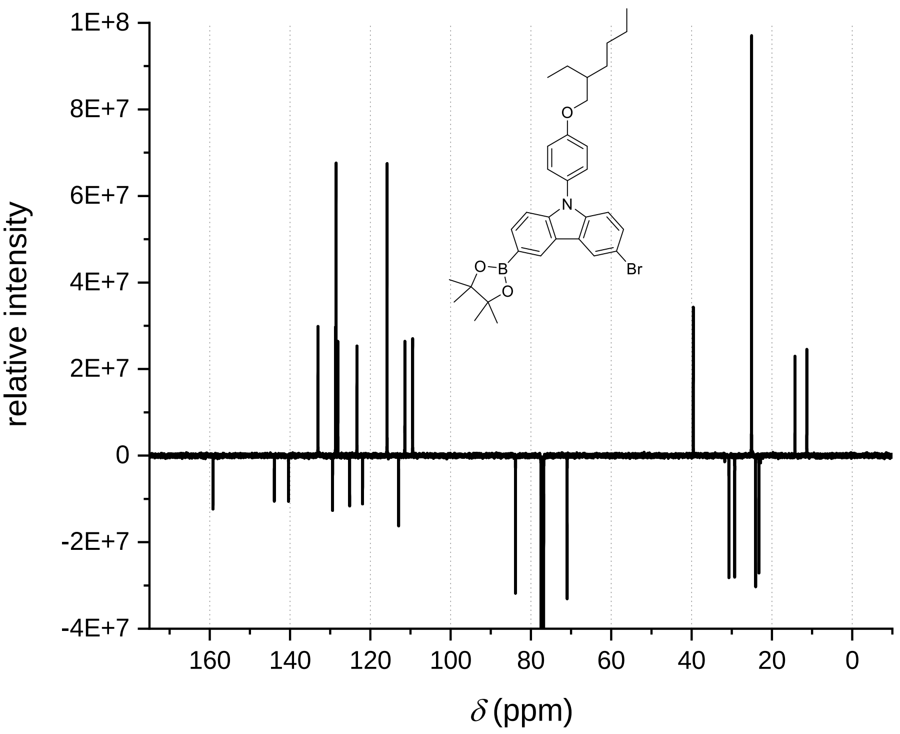

Figure S4. $^{13}C\{^{1}H\}$-APT-NMR spectrum of Bpin-Carb-Br (100 MHz, $CDCl_3$).

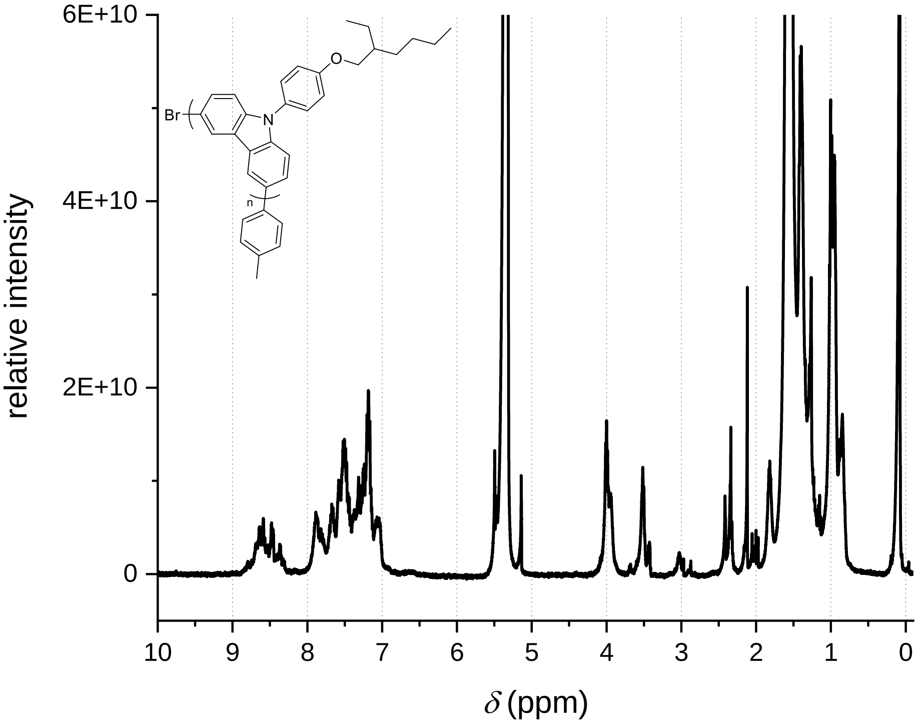

Figure S5. ^{1}H-NMR spectrum of Tol-Carb$_n$-Br (500 MHz, CD_2Cl_2).

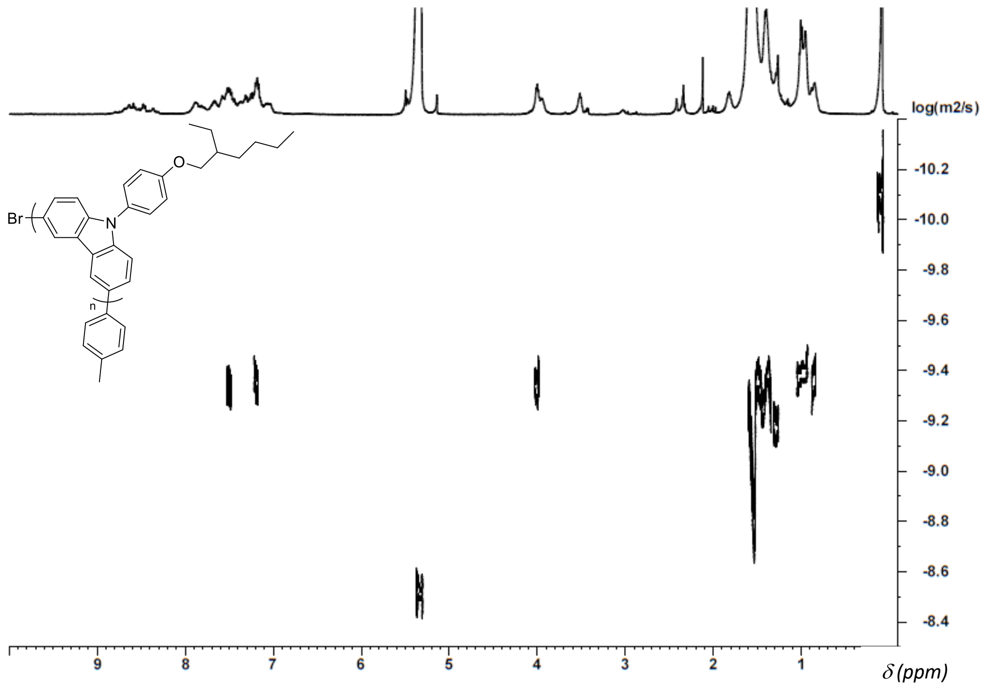

Figure S6. ^{1}H-DOSY-NMR spectrum of Tol-Carb$_n$-Br (500 MHz, CD_2Cl_2).

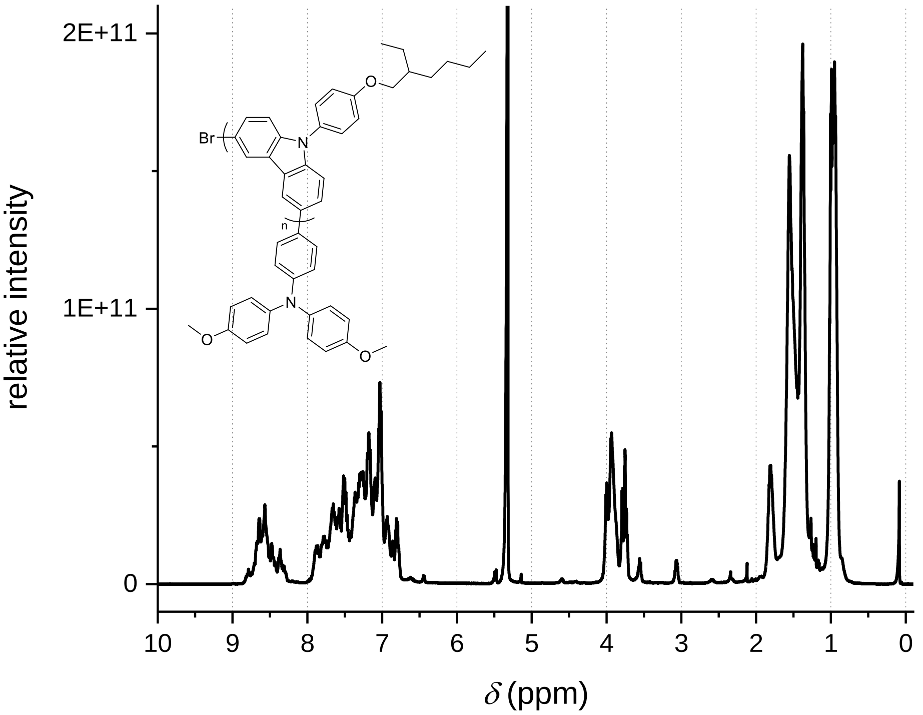

Figure S7. ^{1}H-NMR spectrum of TARA-Carb$_n$-Br (500 MHz, CD_2Cl_2).

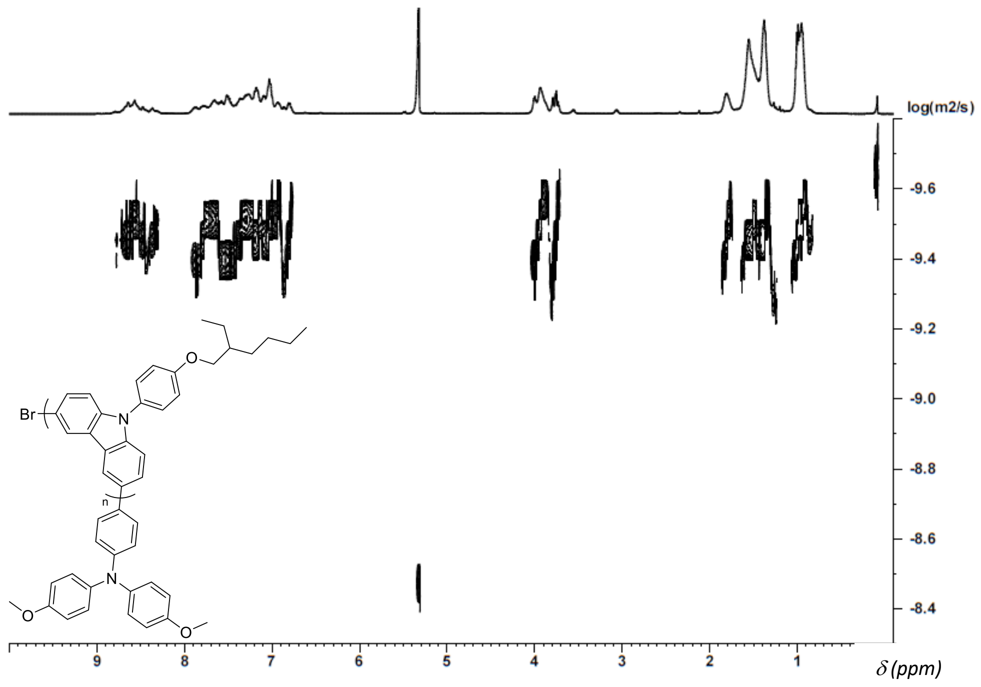

Figure S8. ^{1}H-DOSY-NMR spectrum of TARA-Carb$_n$-Br (500 MHz, CD_2Cl_2).

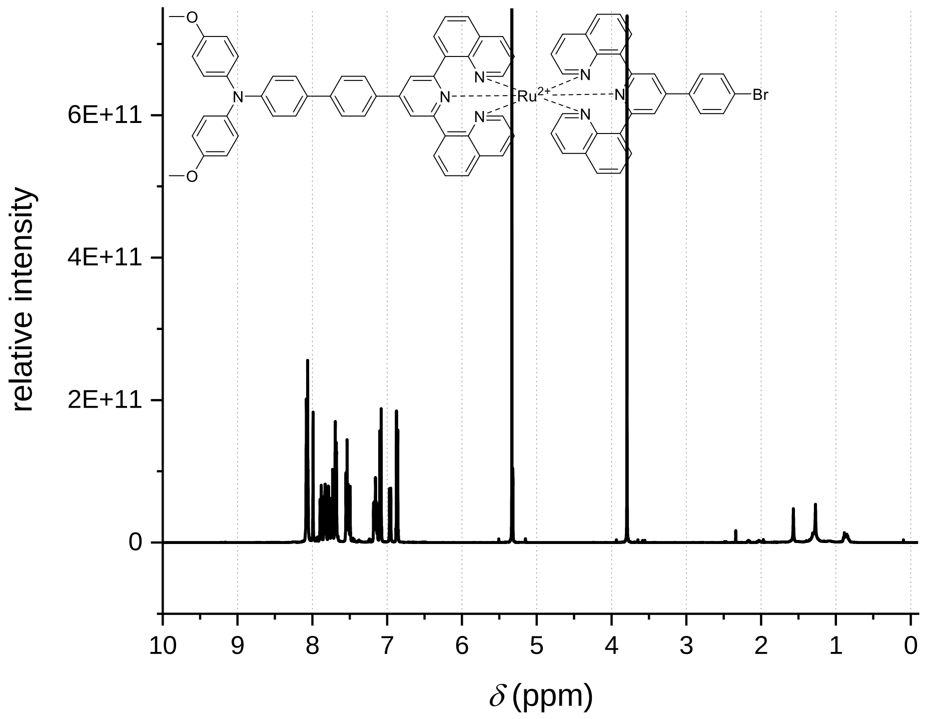

Figure S9. ^{1}H-NMR spectrum of [(TARA-Ph-dqp)Ru(dqp-Ph-Br)]$^{2+}$ (500 MHz, CD_2Cl_2).

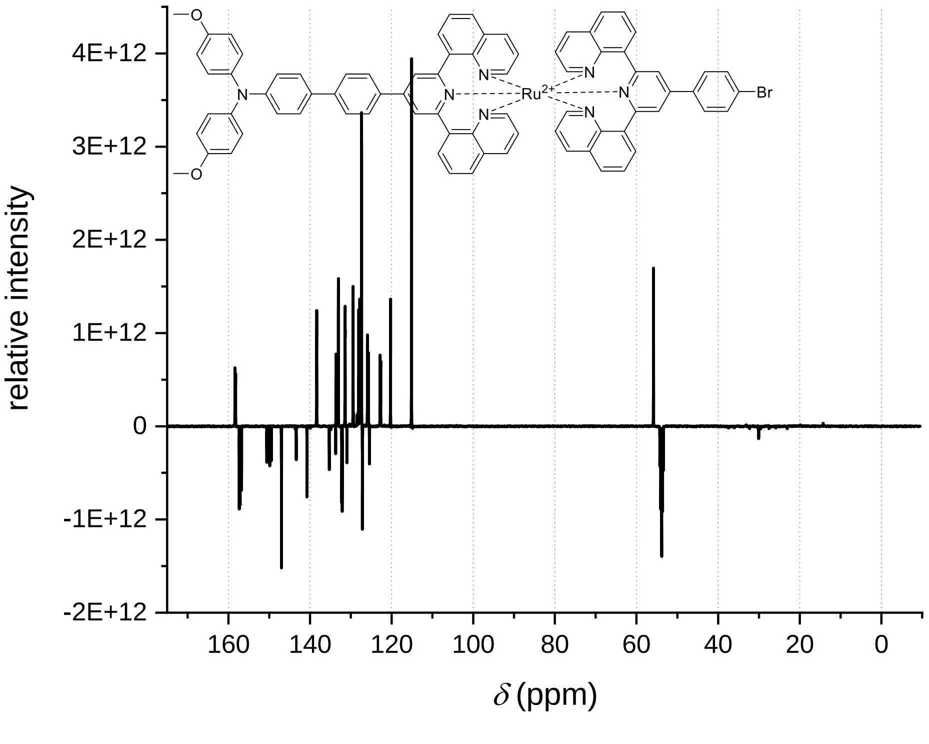

Figure S10. ^{13}C{^{1}H}-APT-NMR spectrum of [(TARA-Ph-dqp)Ru(dqp-Ph-Br)]$^{2+}$ (100 MHz, CD_2Cl_2).

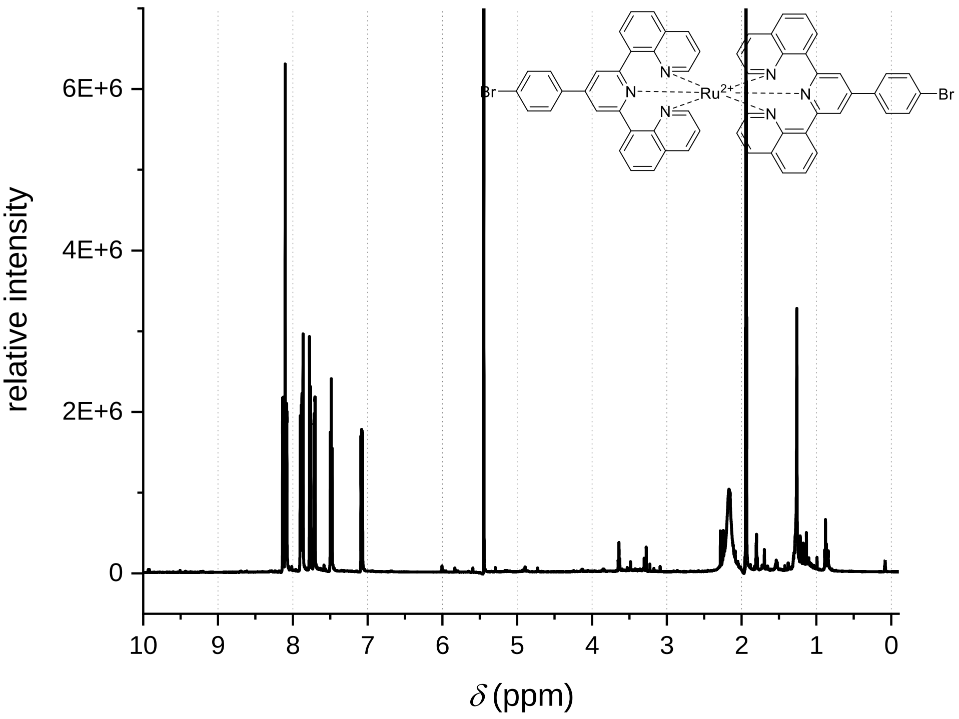

Figure S11. ^{1}H-NMR spectrum of $[Ru(dqp\text{-}Ph\text{-}Br)_2]^{2+}$ (600 MHz, CD_3CN).

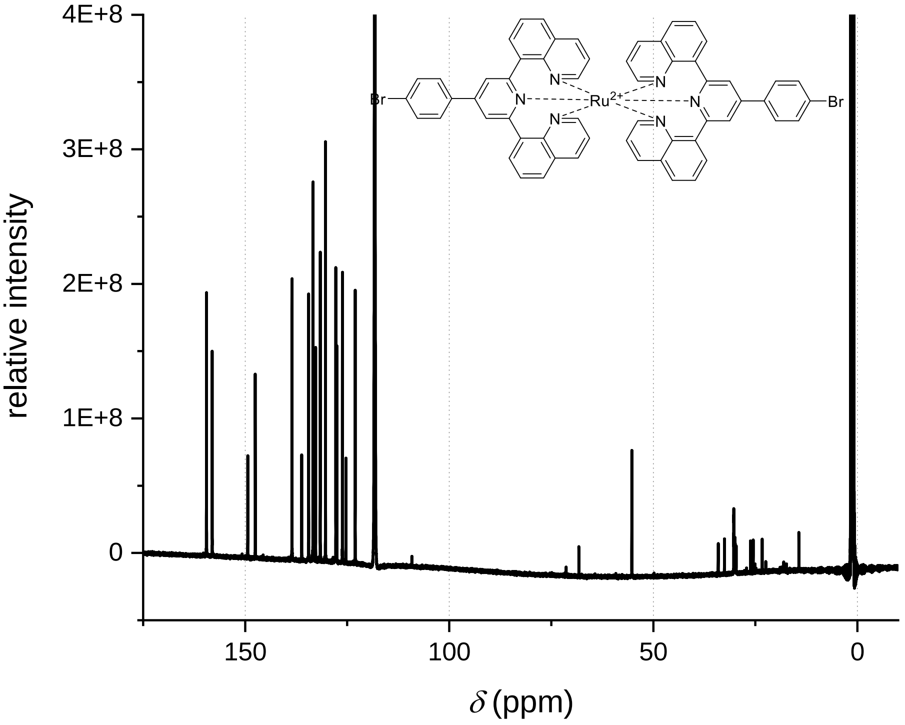

Figure S12. $^{13}C\{^{1}H\}$-NMR spectrum of $[Ru(dqp\text{-}Ph\text{-}Br)_2]^{2+}$ (150 MHz, CD_3CN).

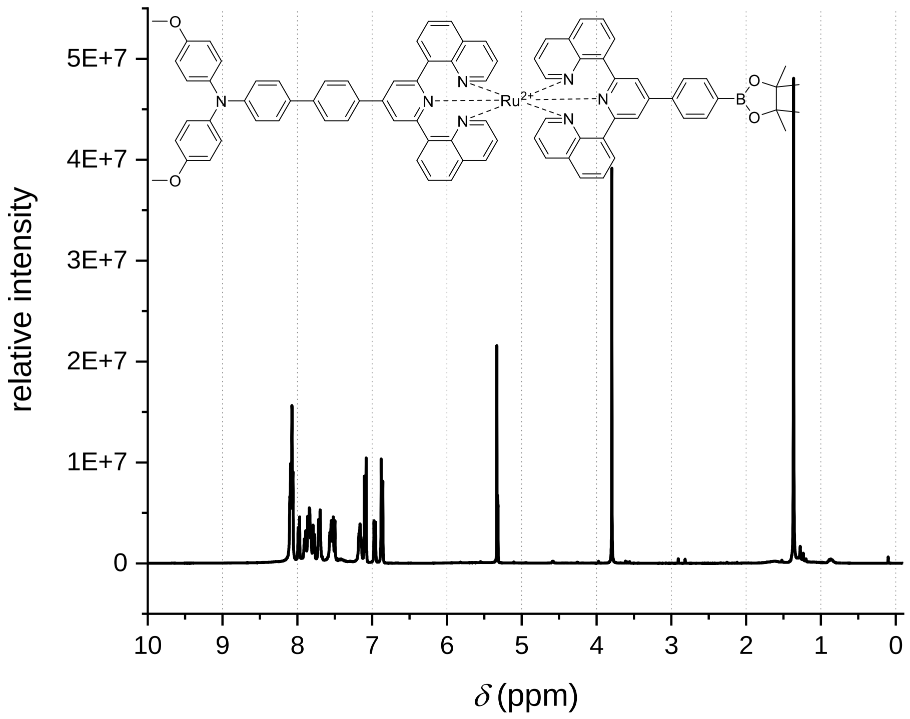

Figure S13. ^{1}H-NMR spectrum of [(TARA-Ph-dqp)Ru(dqp-Ph-Bpin)]$^{2+}$ (400 MHz, CD_2Cl_2).

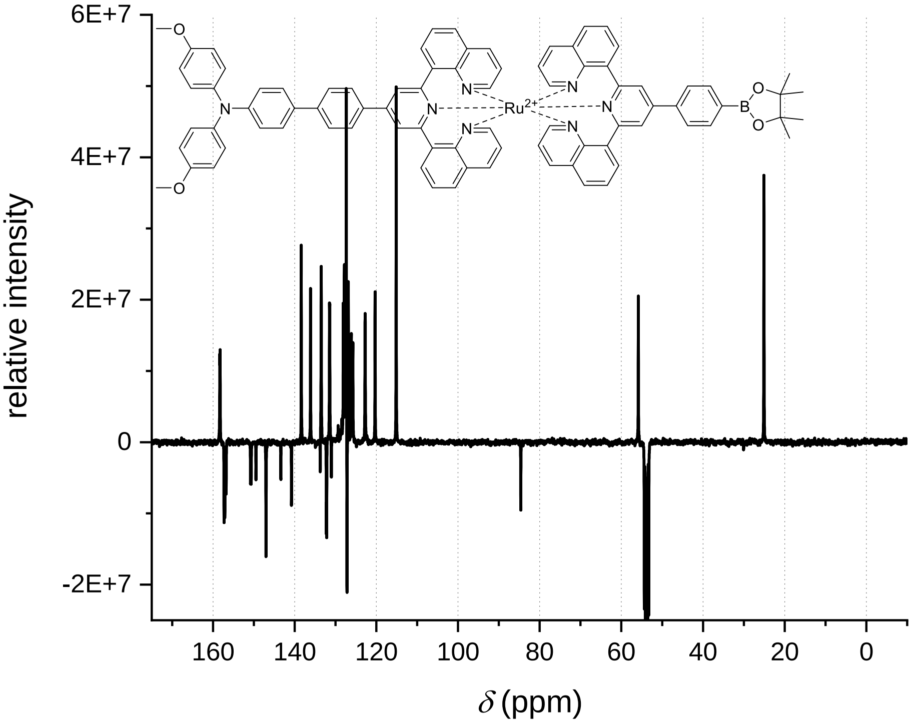

Figure S14. $^{13}C\{^{1}H\}$-APT-NMR spectrum of [(TARA-Ph-dqp)Ru(dqp-Ph-Bpin)]$^{2+}$ (100 MHz, CD_2Cl_2).

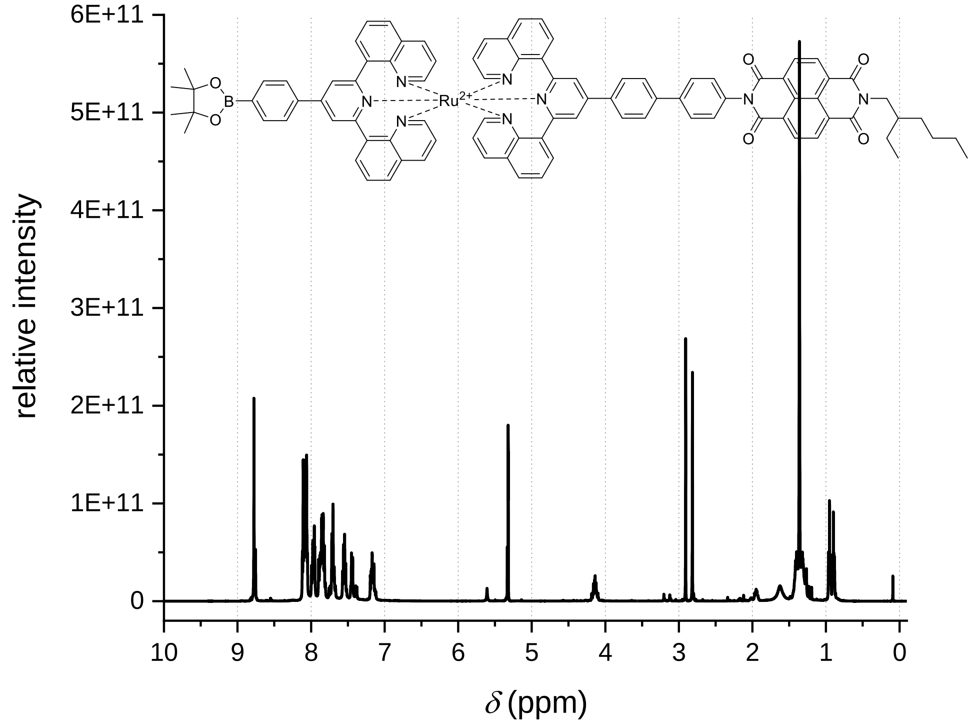

Figure S15. ^{1}H-NMR spectrum of [(Bpin-Ph-dqp)Ru(dqp-Ph-Ph-NDI)]$^{2+}$ (500 MHz, CD_2Cl_2).

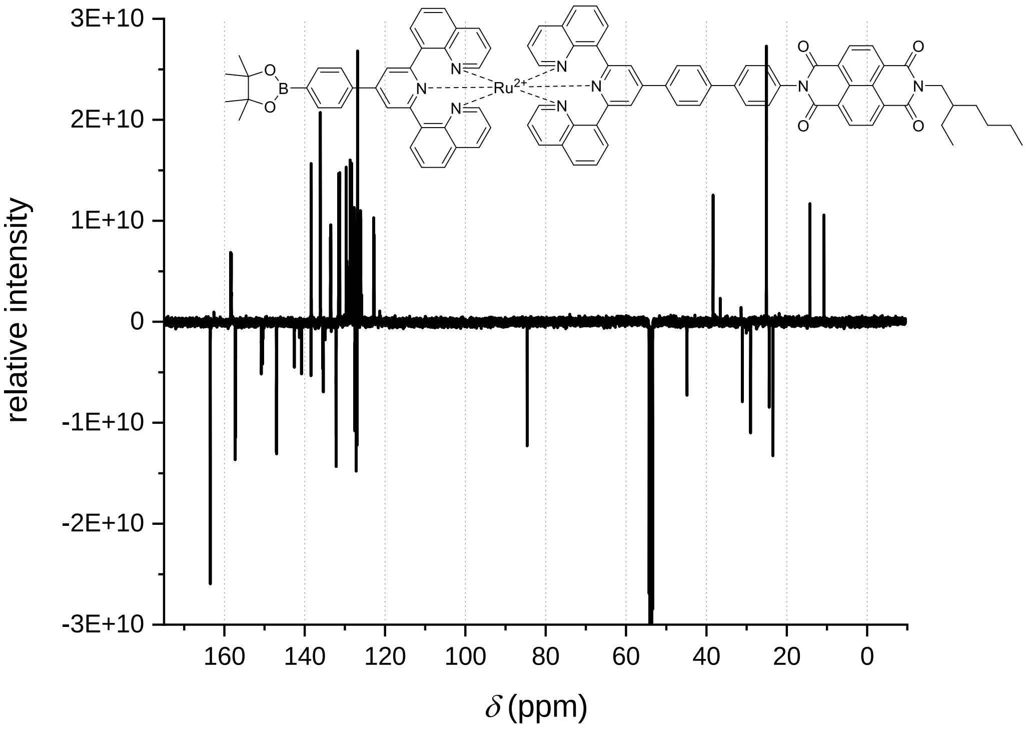

Figure S16. ^{13}C{^{1}H}-APT- NMR spectrum of [(Bpin-Ph-dqp)Ru(dqp-Ph-Ph-NDI)]$^{2+}$ (125 MHz, CD_2Cl_2).

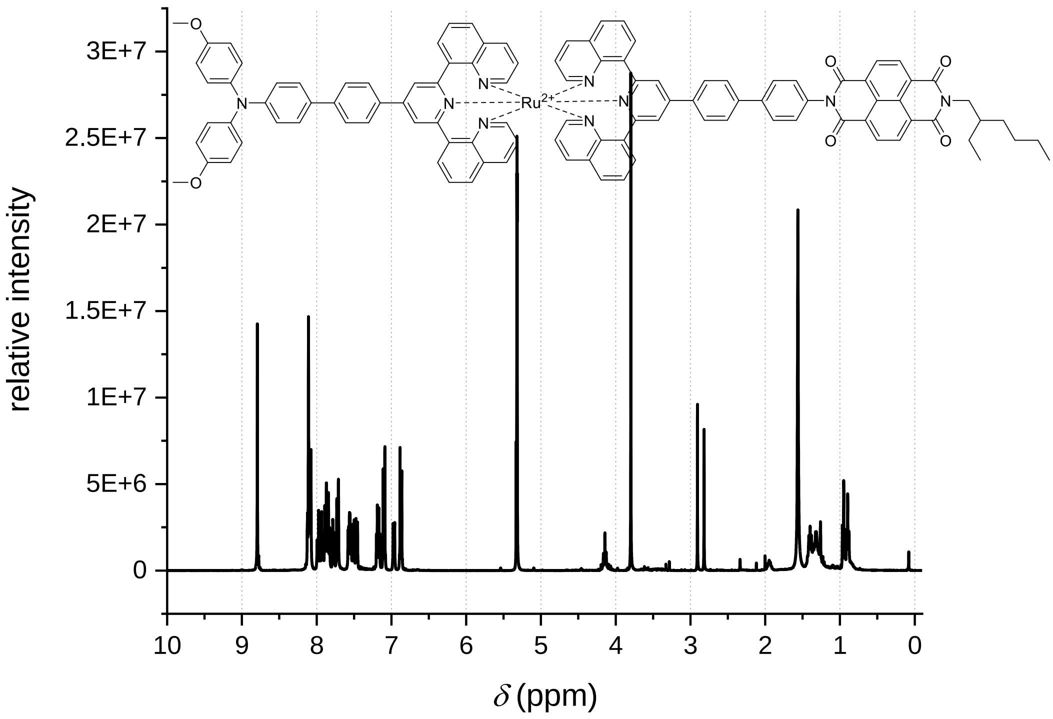

Figure S17. ^{1}H-NMR spectrum of [(TARA-Ph-dqp)Ru(dqp-Ph-Ph-NDI)]$^{2+}$ (400 MHz, CD_2Cl_2).

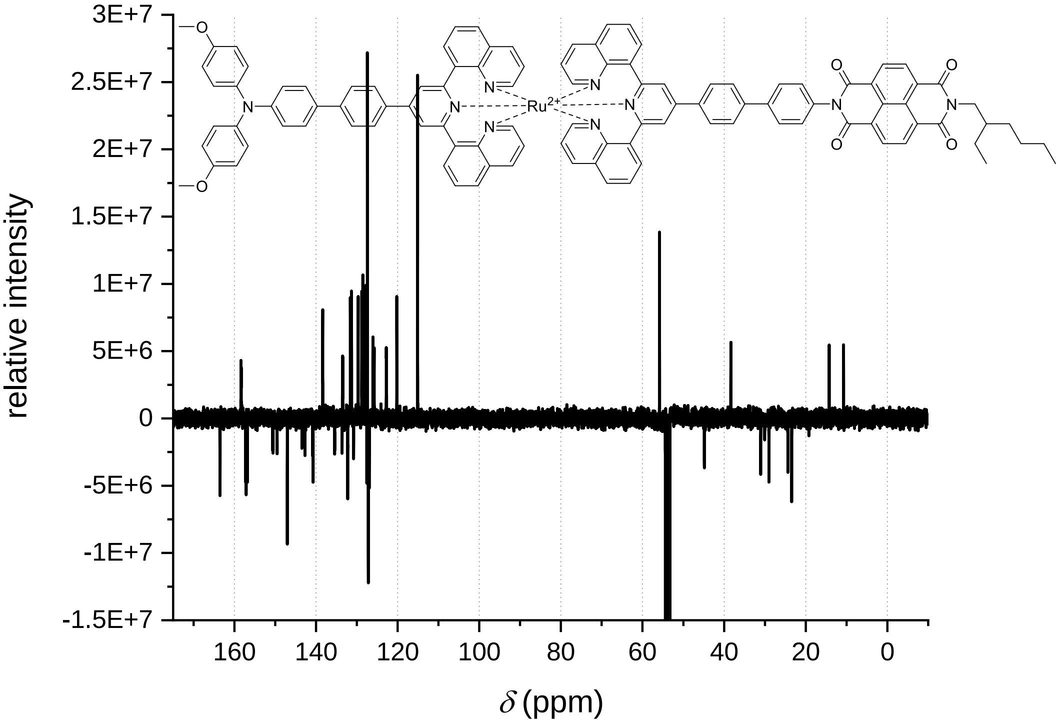

Figure S18. ^{13}C{^{1}H}-APT- NMR spectrum of [(TARA-Ph-dqp)Ru(dqp-Ph-Ph-NDI)]$^{2+}$ (100 MHz, CD_2Cl_2).

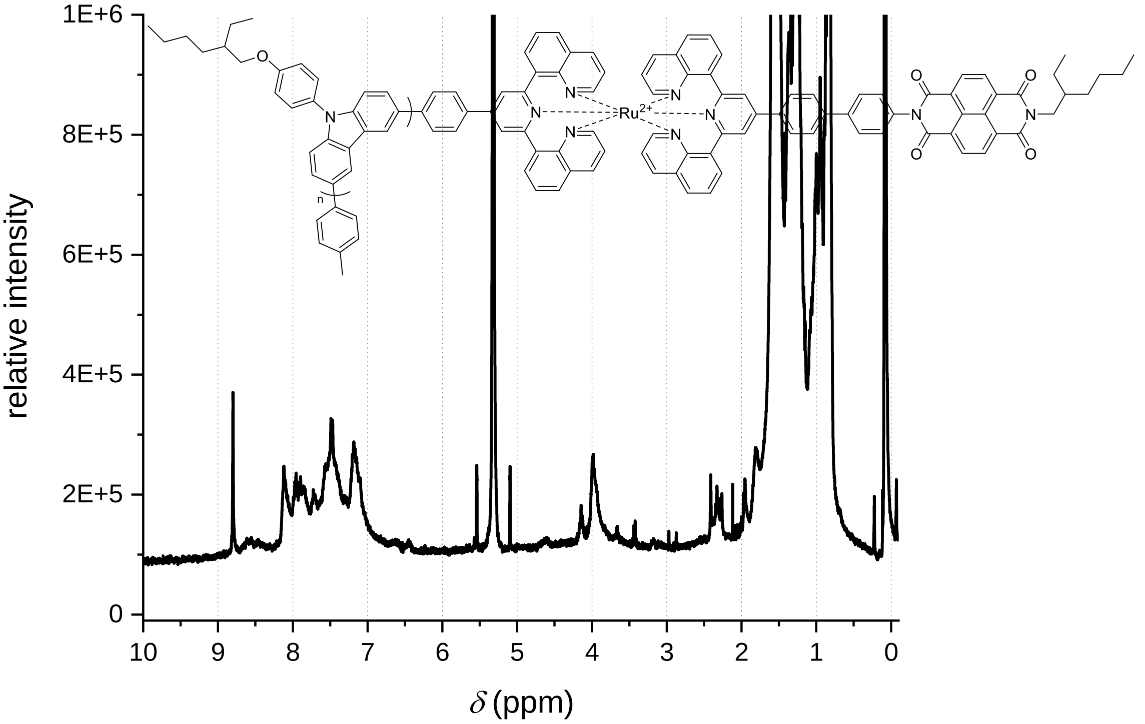

Figure S19. ^{1}H-NMR spectrum of [(Tol-Carb$_n$-Ph-dqp)Ru(dqp-Ph-Ph-NDI)]$^{2+}$ (400 MHz, CD_2Cl_2).

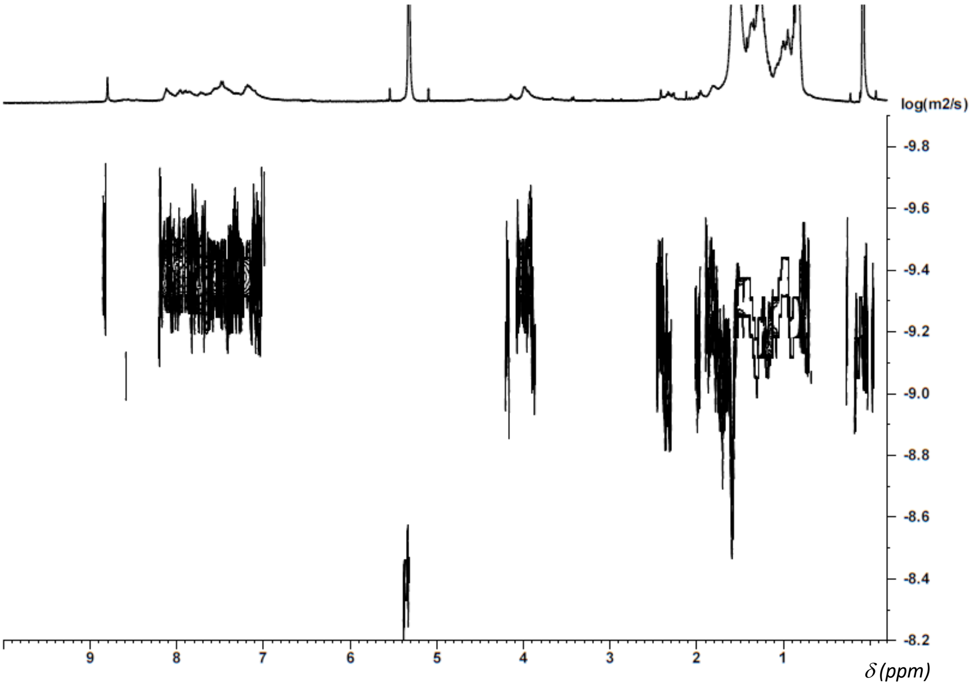

Figure S20. ^{1}H-DOSY-NMR spectrum of [(Tol-Carb$_n$-Ph-dqp)Ru(dqp-Ph-Ph-NDI)]$^{2+}$ (400 MHz, CD_2Cl_2).

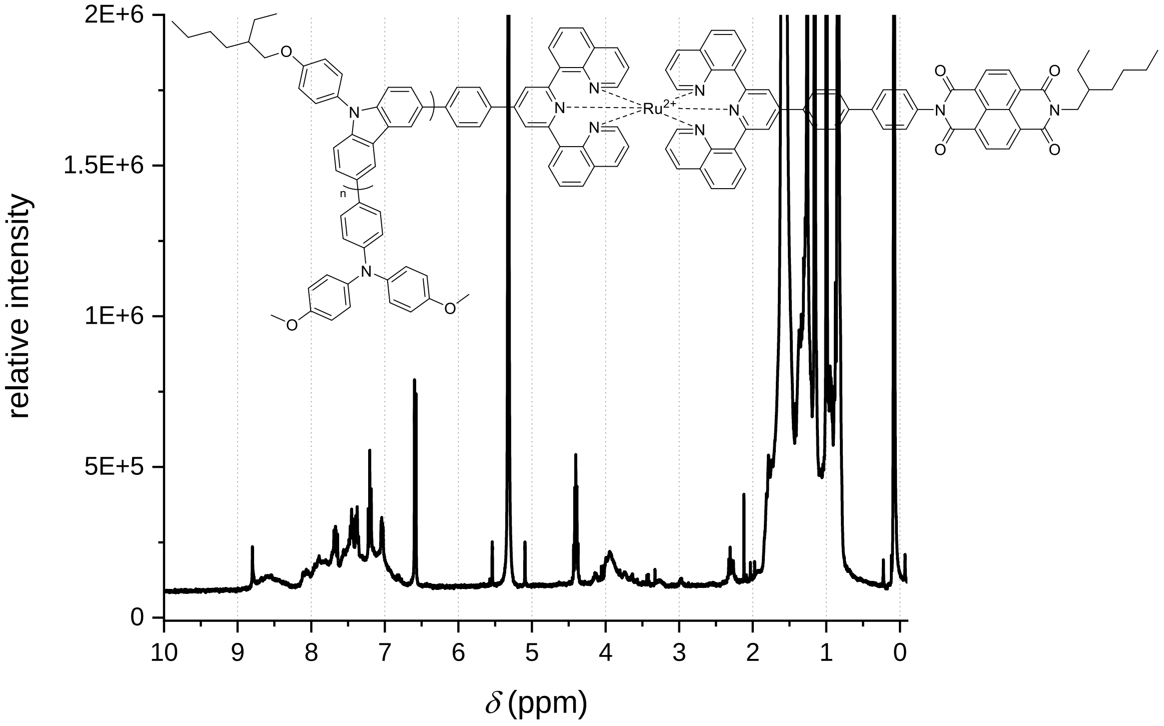

Figure S21. ^{1}H-NMR spectrum of [(TARA-Carb$_n$-Ph-dqp)Ru(dqp-Ph-Ph-NDI)]$^{2+}$ (400 MHz, CD_2Cl_2).

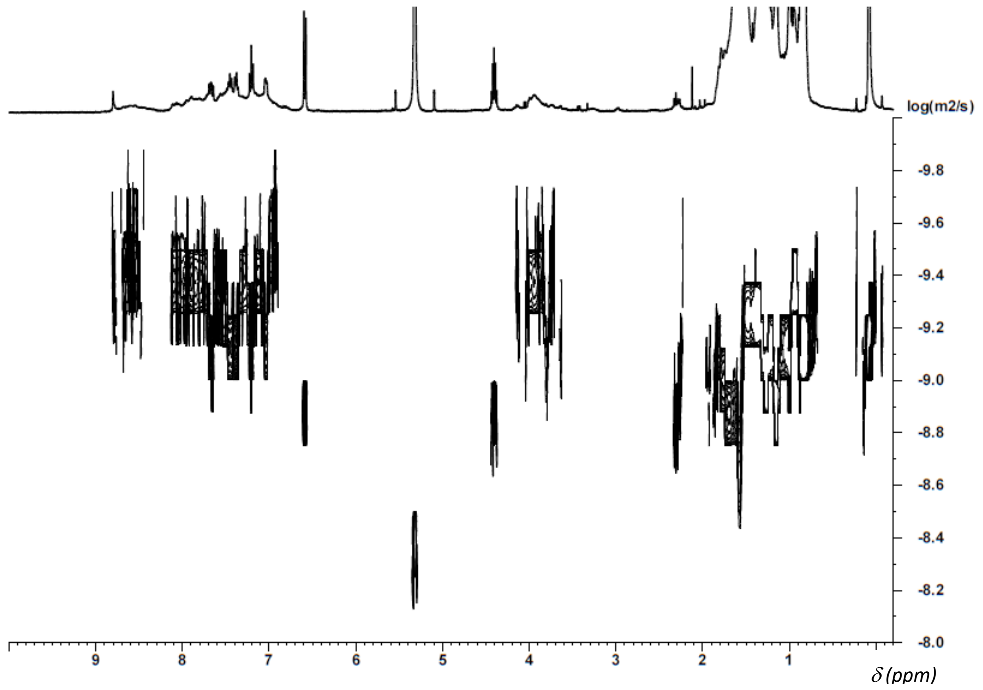

Figure S22. ^{1}H-DOSY-NMR spectrum of [(TARA-Carb$_n$-Ph-dqp)Ru(dqp-Ph-Ph-NDI)]$^{2+}$ (400 MHz, CD_2Cl_2).

2.2 Comparsion

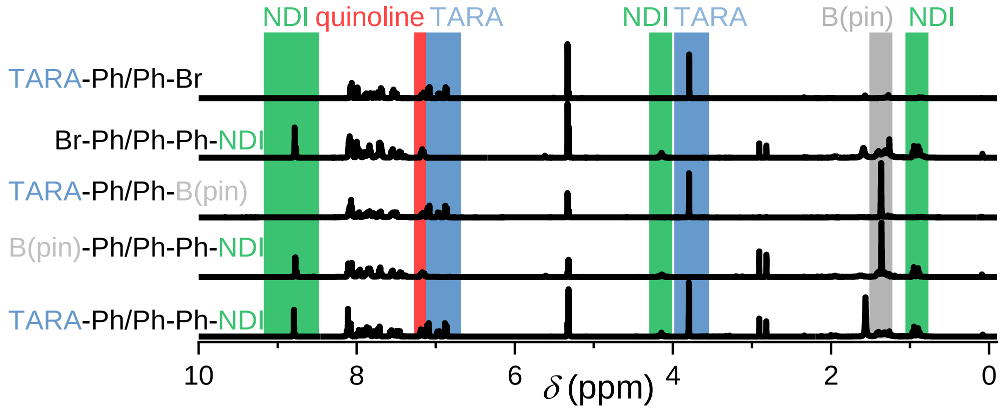

Figure S23: ^{1}H-NMR spectra (CD_2Cl_2, 400 MHz or 500 MHz) of $[(TARA-Ph-dqp)Ru(dqp-Ph-Br]^{2+}$ (TARA-Ph/Ph-Br), $[(Br-Ph-dqp)Ru(dqp-Ph-Ph-NDI]^{2+}$ (Br-Ph/Ph-Ph-NDI), $[(TARA-Ph-dqp)Ru(dqp-Ph-Bpin]^{2+}$ (TARA-Ph/Ph-Bpin), $[(Bpin-Ph-dqp)Ru(dqp-Ph-Ph-NDI]^{2+}$ (Bpin-Ph/Ph-Ph-NDI), and $[(TARA-Ph-dqp)Ru(dqp-Ph-Ph-NDI]^{2+}$ (TARA-Ph/Ph-Ph-NDI). Boxes mark characteristic signals for NDI (green), quinoline (red), TARA (blue), or B(pin)-assigned signals. Br-Ph/Ph-Ph-NDI characterization reproduced for clarity.[8]

3 Mass spectrometry

3.1 ESI-ToF MS data.

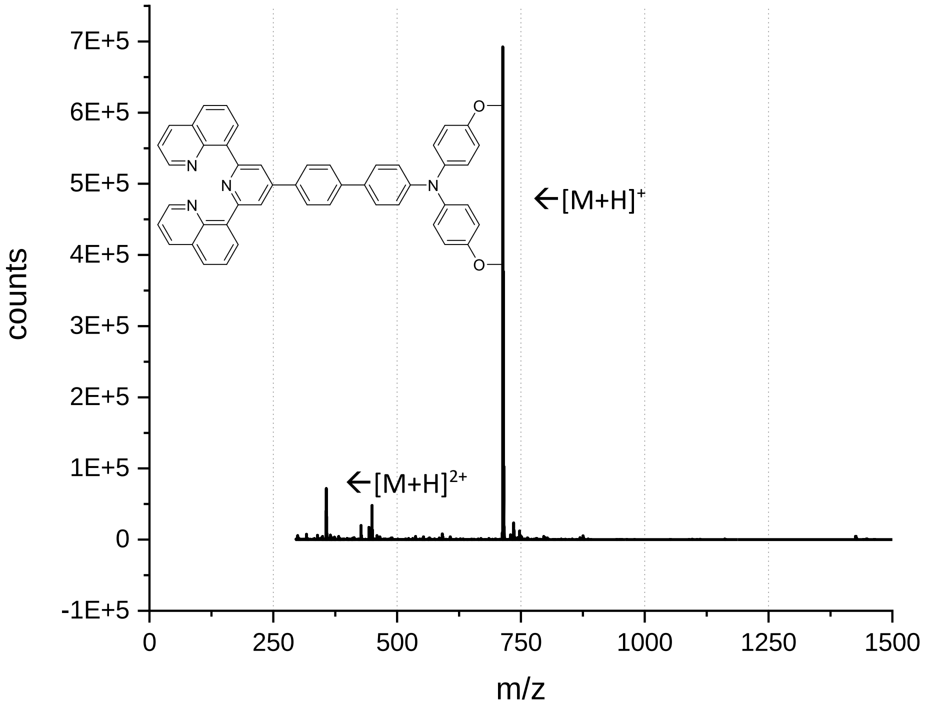

Figure S24. ESI-ToF mass spectrum of dqp-Ph-TARA.

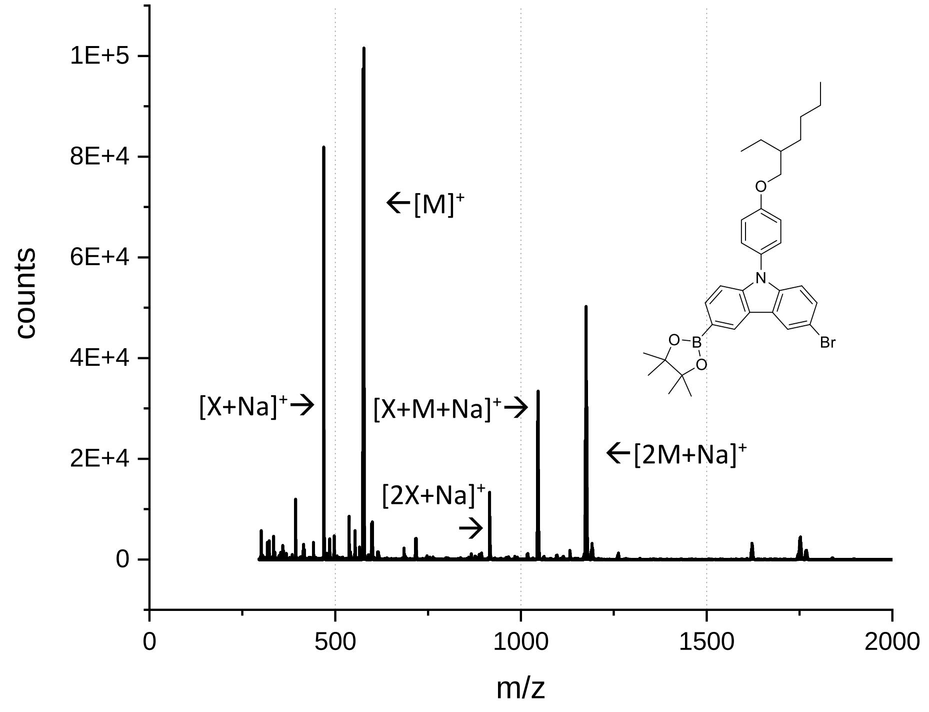

Figure S25. ESI-ToF mass spectrum of Bpin-Carb-Br. Note the unassigned species X. Its isotope pattern indicates neither Br or B as part of the sum formula. The high signal intensity does not fit the excellent NMR-spectra, thus is assigned to contaminations in the instrument, or fragmentation processes. In addition, a contamination without both functional groups would not take part in the polymerization process.

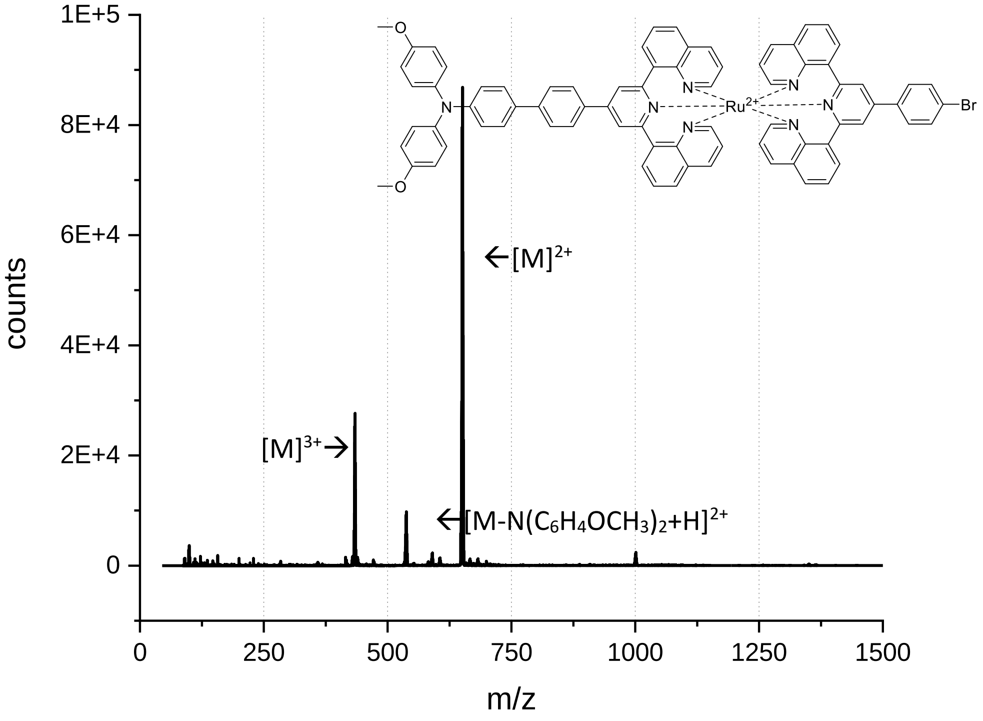

Figure S26. ESI-ToF mass spectrum of [(TARA-Ph-dqp)Ru(dqp-Ph-Br)]$^{2+}$.

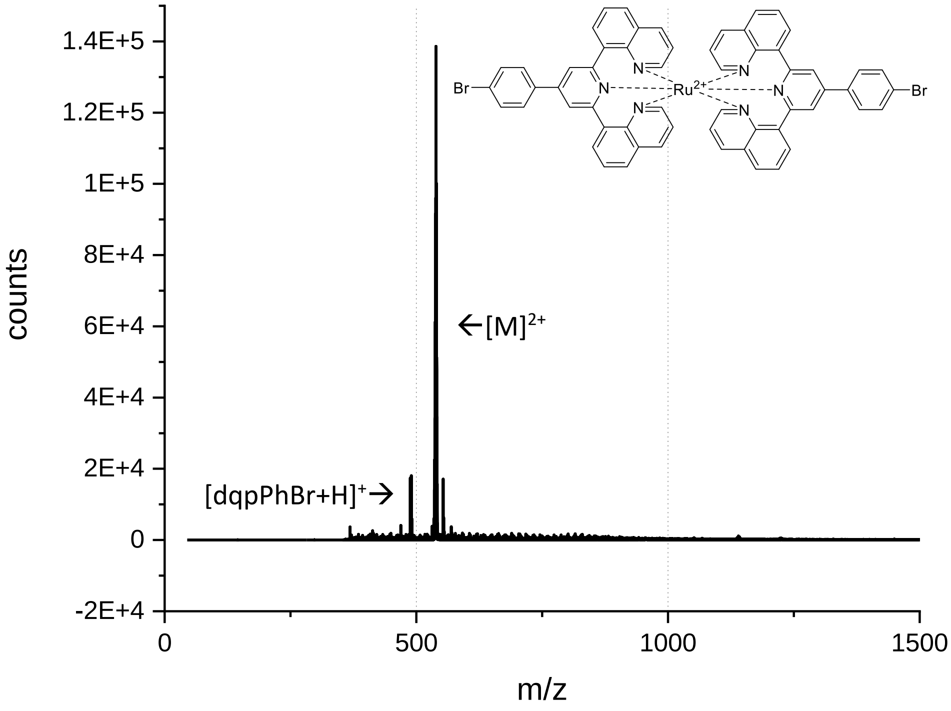

Figure S27. ESI-ToF mass spectrum of [Ru(dqp-Ph-Br)$_2$]$^{2+}$.

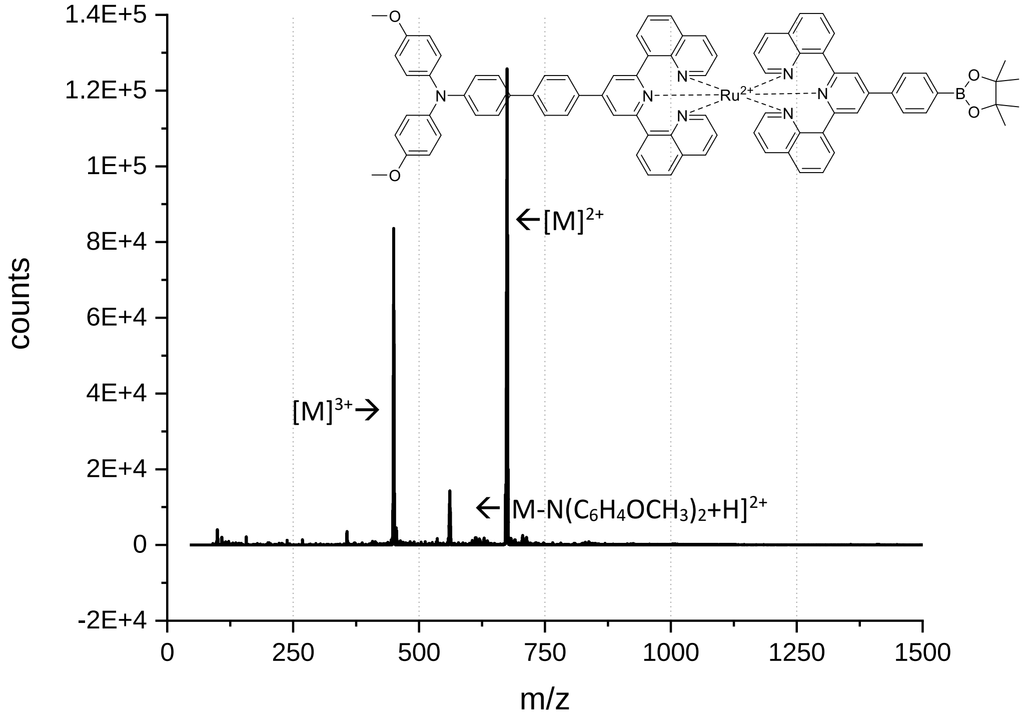

Figure S28. ESI-ToF mass spectrum of [(TARA-Ph-dqp)Ru(dqp-Ph-Bpin)]$^{2+}$.

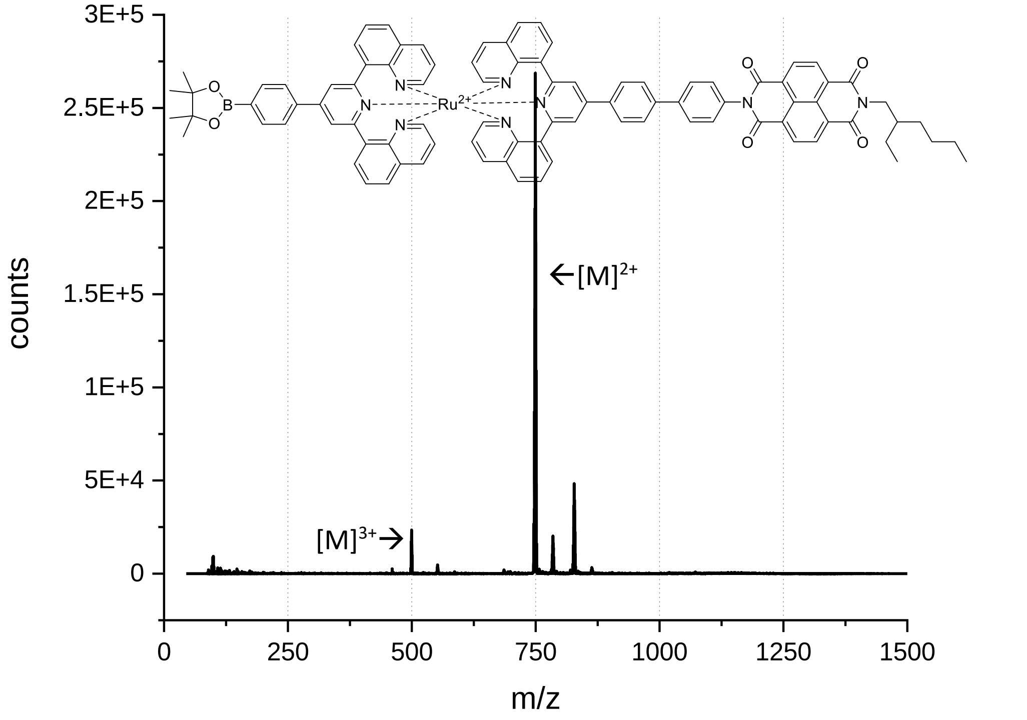

Figure S29. ESI-ToF mass spectrum of [(Bpin-Ph-dqp)Ru(dqp-Ph-Ph-NDI)]$^{2+}$.

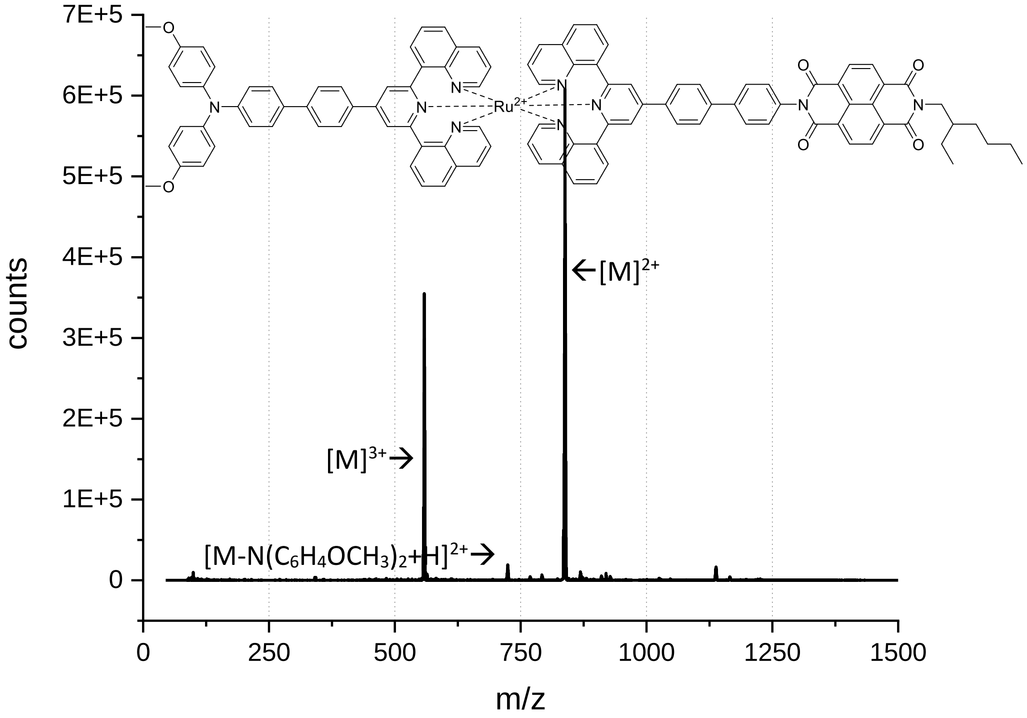

Figure S30. ESI-ToF mass spectrum of $[(TARA\text{-}Ph\text{-}dqp)Ru(dqp\text{-}Ph\text{-}Ph\text{-}NDI)]^{2+}$.

3.2 MALDI-ToF MS data

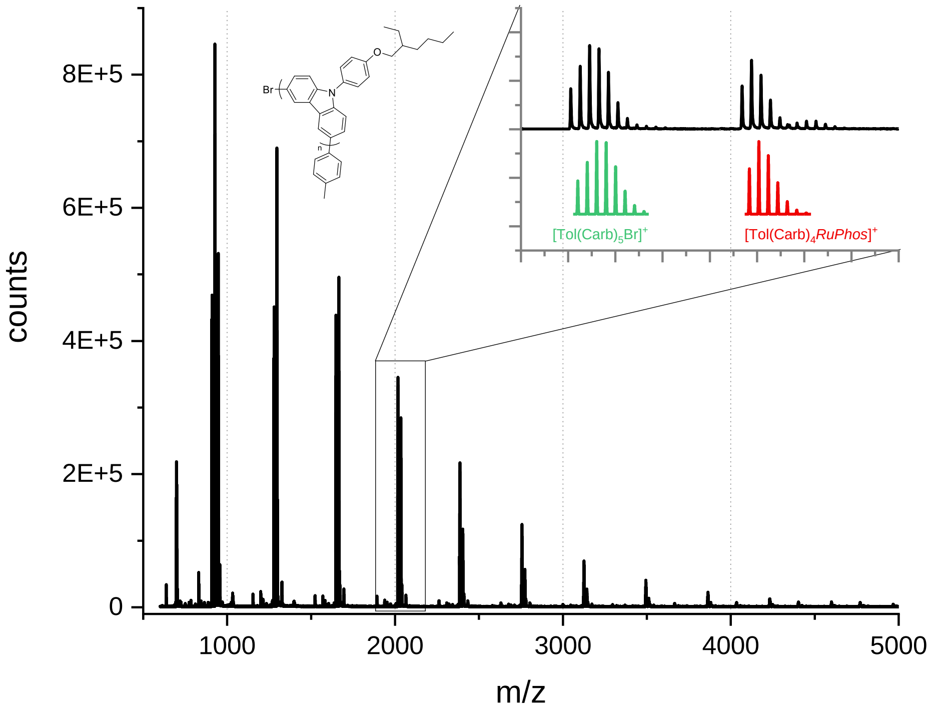

Figure S31. MALDI-ToF mass spectrum of Tol-Carb$_n$-Br.

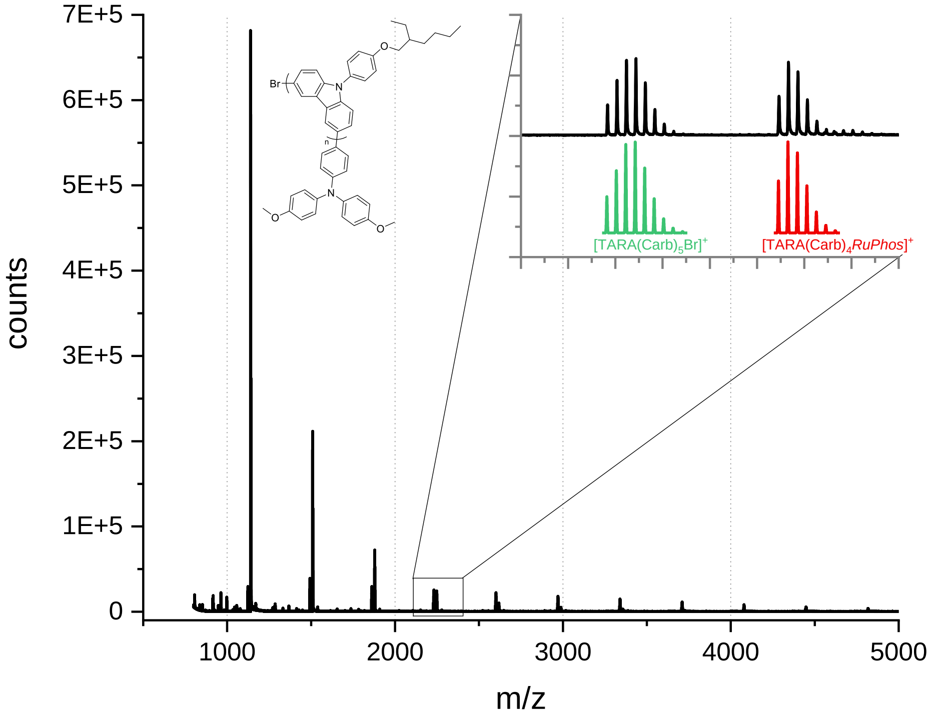

Figure S32. MALDI-ToF mass spectrum of TARA-Carb$_n$-Br.

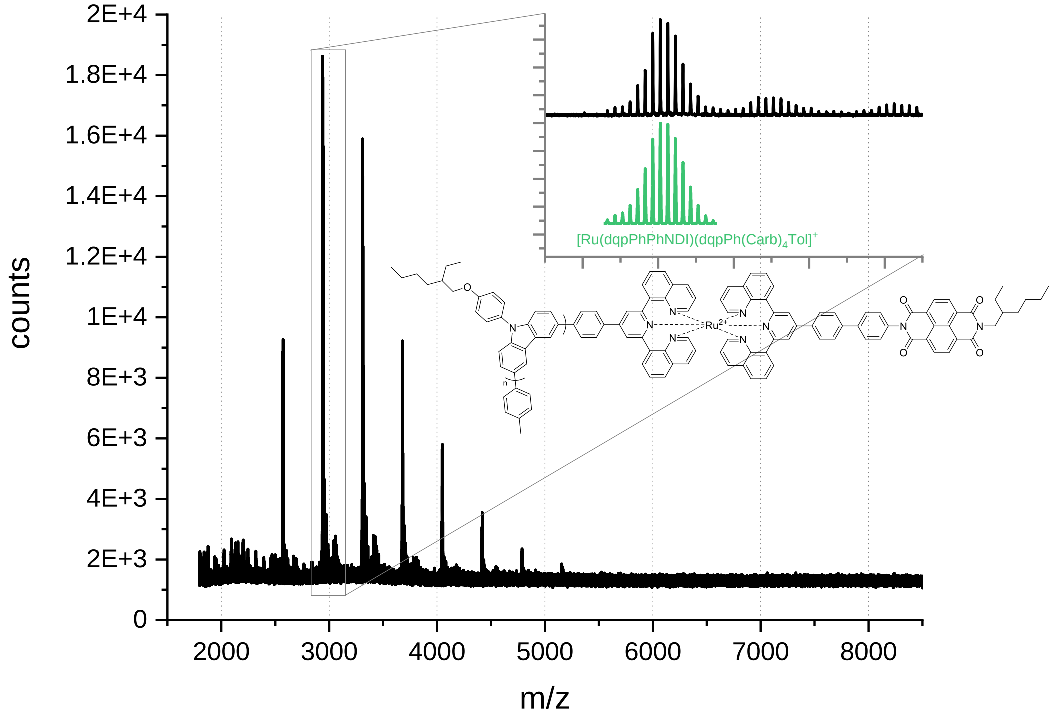

Figure S33. MALDI-ToF mass spectrum of [(Tol-Carb$_n$-Ph-dqp)Ru(dqp-Ph-Ph-NDI)]$^{2+}$.

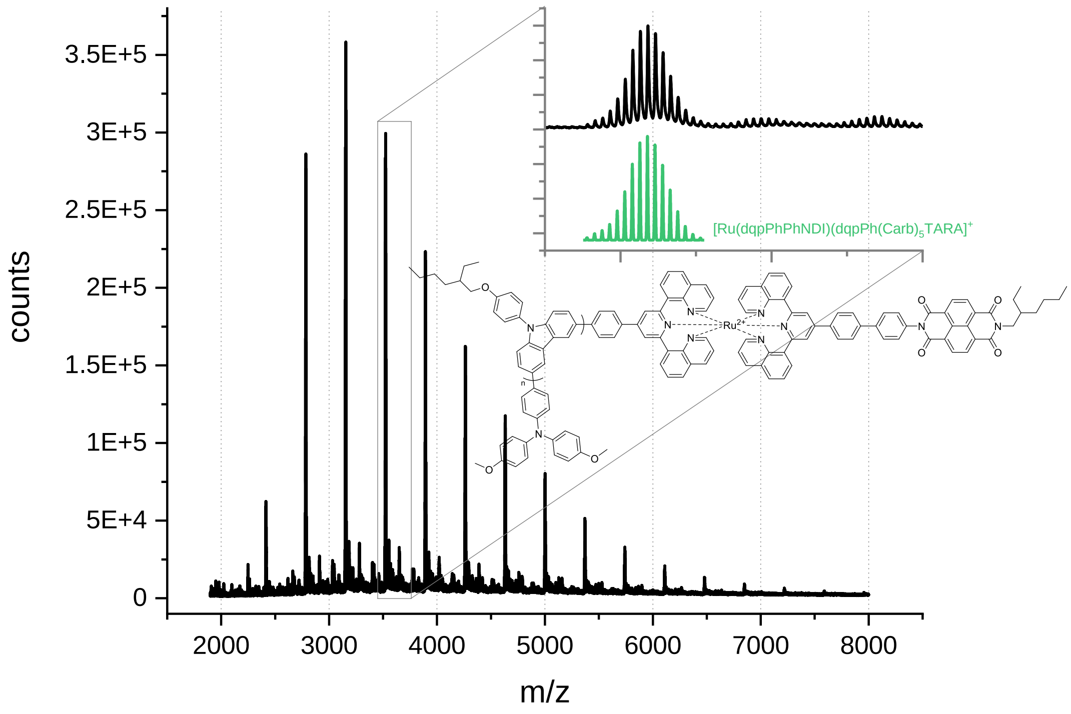

Figure S34. MALDI-ToF mass spectrum of [(TARA-Carb$_n$-Ph-dqp)Ru(dqp-Ph-Ph-NDI)]$^{2+}$.

3.3 Comparison

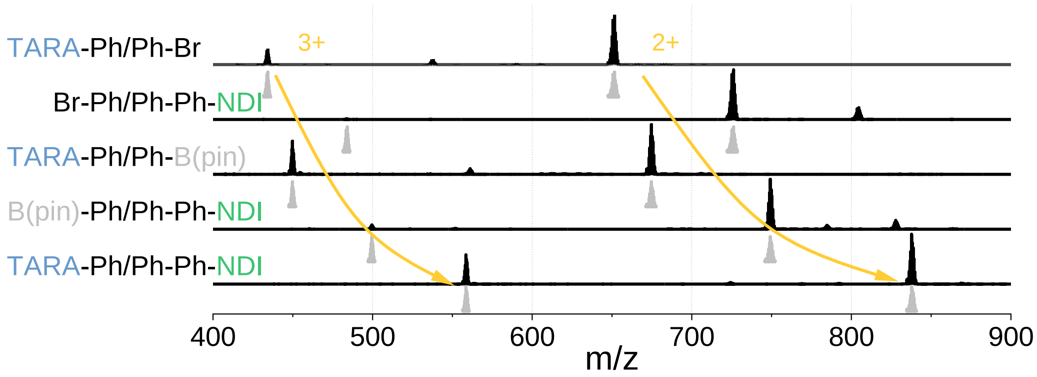

Figure S35. ESI-ToF mass spectra of [(TARA-Ph-dqp)Ru(dqp-Ph-Br]$^{2+}$ (TARA-Ph/Ph-Br), [(Br-Ph-dqp)Ru(dqp-Ph-Ph-NDI]$^{2+}$ (Br-Ph/Ph-Ph-NDI), [(TARA-Ph-dqp)Ru(dqp-Ph-Bpin]$^{2+}$ (TARA-Ph/Ph-Bpin), [(Bpin-Ph-dqp)Ru(dqp-Ph-Ph-NDI]$^{2+}$ (Bpin-Ph/Ph-Ph-NDI), and [(TARA-Ph-dqp)Ru(dqp-Ph-Ph-NDI]$^{2+}$ (TARA-Ph/Ph-Ph-NDI). Gray isotope patterns are calculated for species assignment. Yellow arrows indicate series of twofold (2+) or threefold (3+) charged species. Br-Ph/Ph-Ph-NDI characterization reproduced for clarity.[8]

4 Size exclusion chromatography

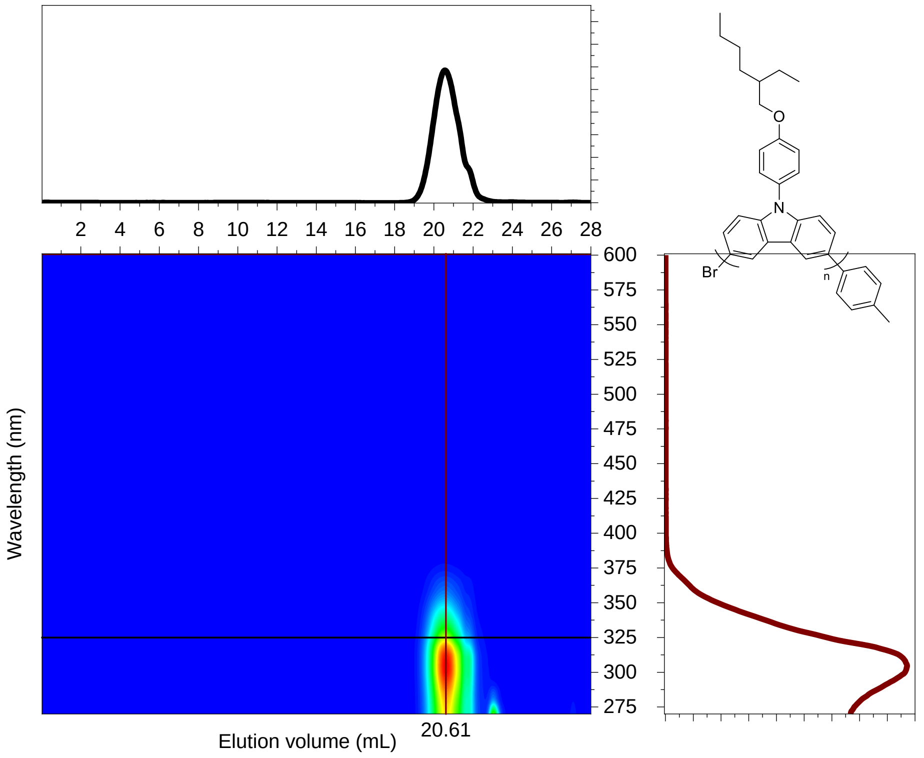

Figure S36. SEC elugram of Tol-Carb$_n$-Br (DMAc + 0.08 wt% NH_4PF_6).

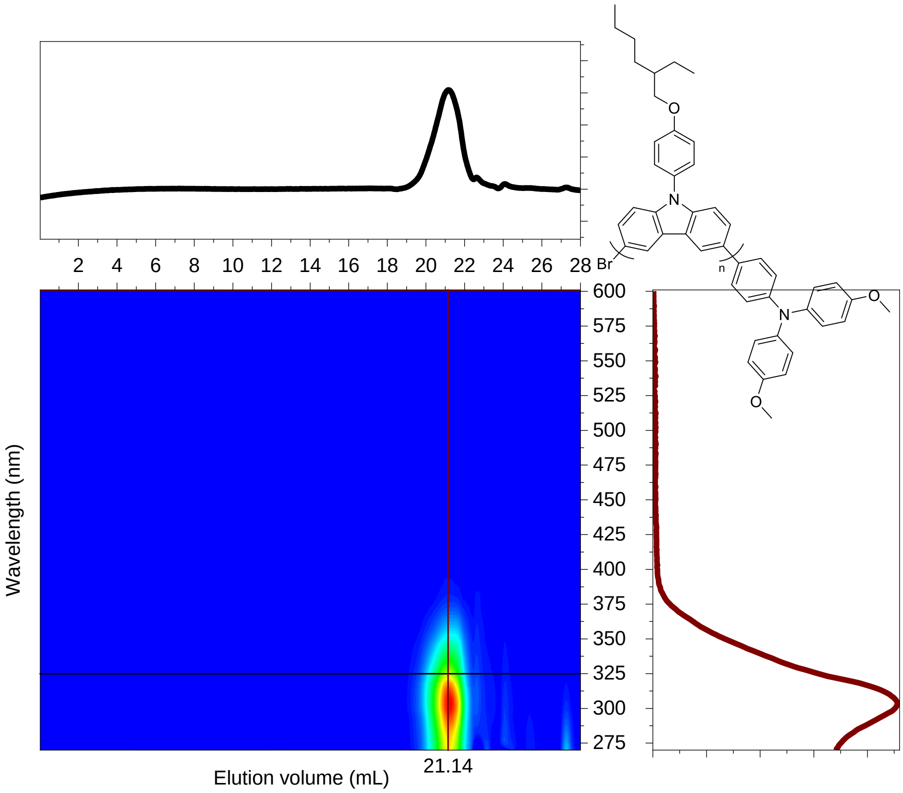

Figure S37. SEC elugram of TARA-Carb$_n$-Br (DMAc + 0.08 wt% NH_4PF_6).

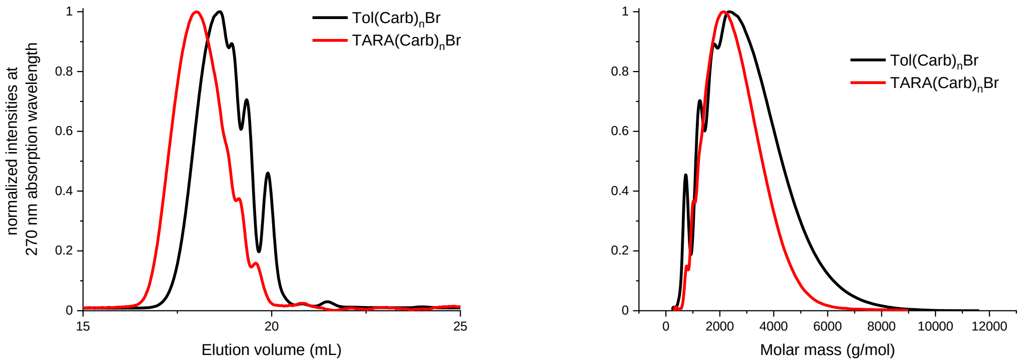

Figure S38. Left: SEC elugrams of Tol-Carb$_n$-Br (black) and TARA-Carb$_n$-Br (red) for determination of molar masses with detection at 270 nm absorption wavelength. Right: SEC elugrams of of Tol-Carb$_n$-Br (black) and TARA-Carb$_n$-Br (red) against molar mass according to a PS calibration ($CHCl_3$/iPrOH/NEt_3 94:2:4).

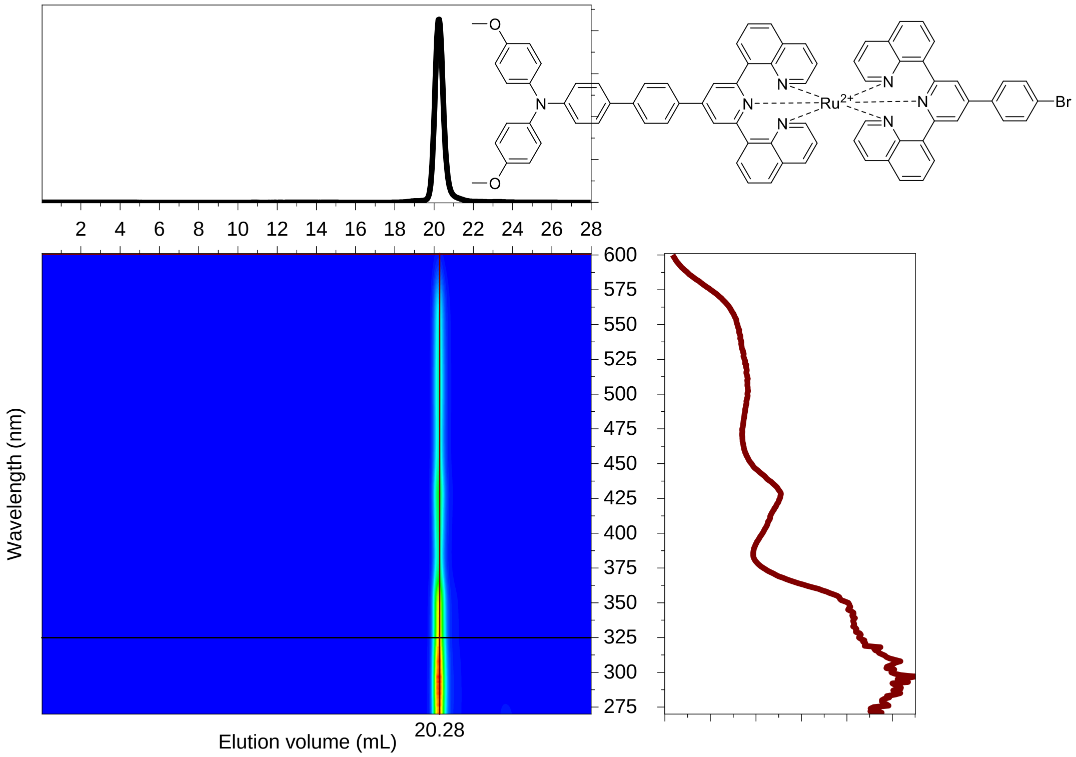

Figure S39. SEC elugram of $[(TARA\text{-}Ph\text{-}dqp)Ru(dqp\text{-}Ph\text{-}Br)]^{2+}$ (DMAc + 0.08 wt% NH_4PF_6).

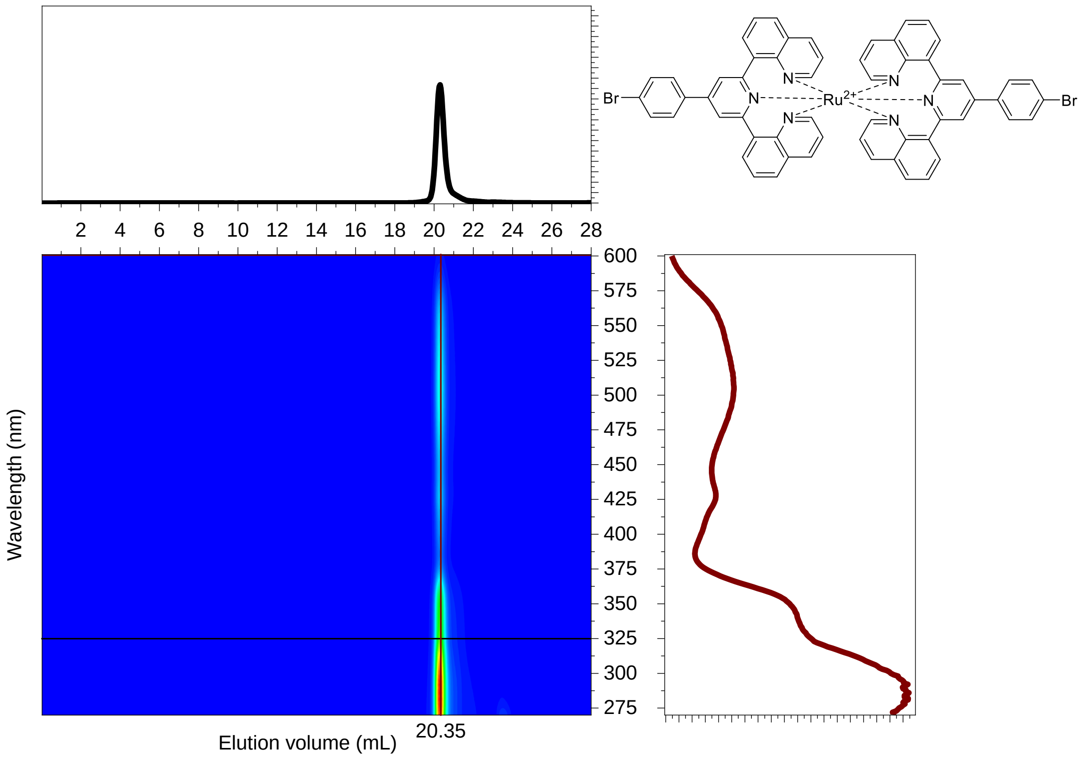

Figure S40. SEC elugram of $[Ru(dqp\text{-}Ph\text{-}Br)_2]^{2+}$ (DMAc + 0.08 wt% NH_4PF_6).

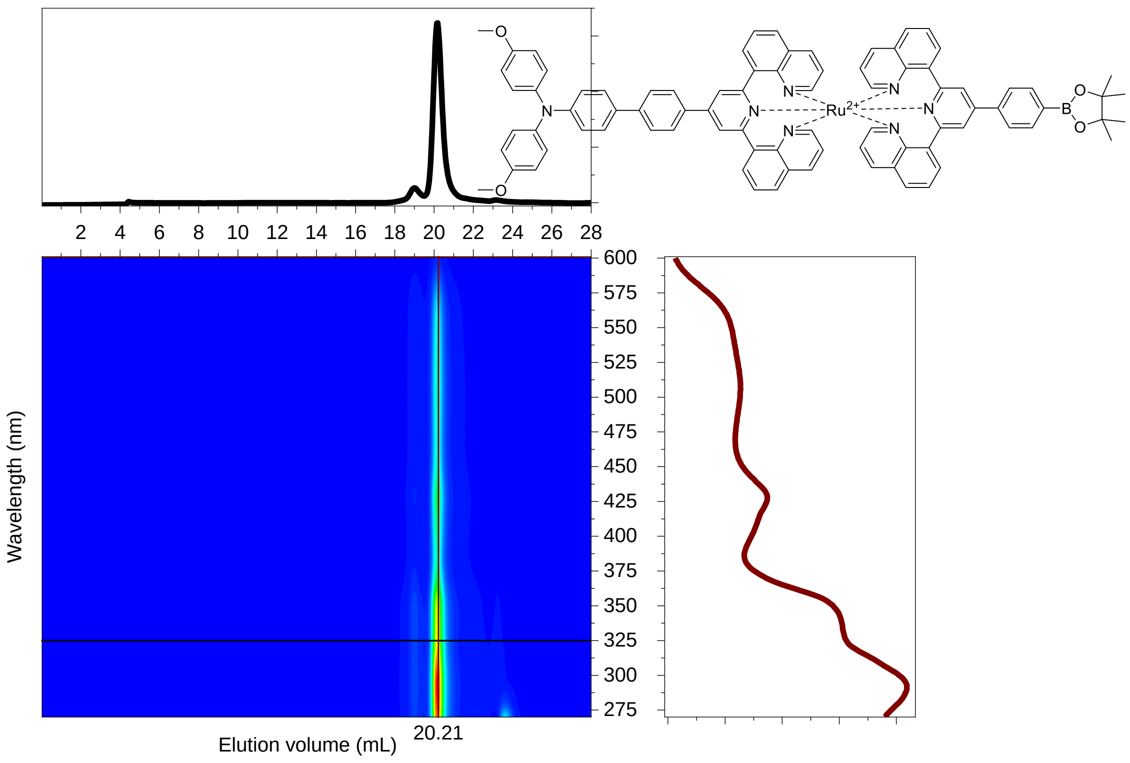

Figure S41. SEC elugram of [(TARA-Ph-dqp)Ru(dqp-Ph-Bpin)]$^{2+}$ (DMAc + 0.08 wt% NH_4PF_6). Note the second species around 18.5 mL, assigned to the dimeric coupling product, deriving from the conversion of bromide into boronic acid ester.

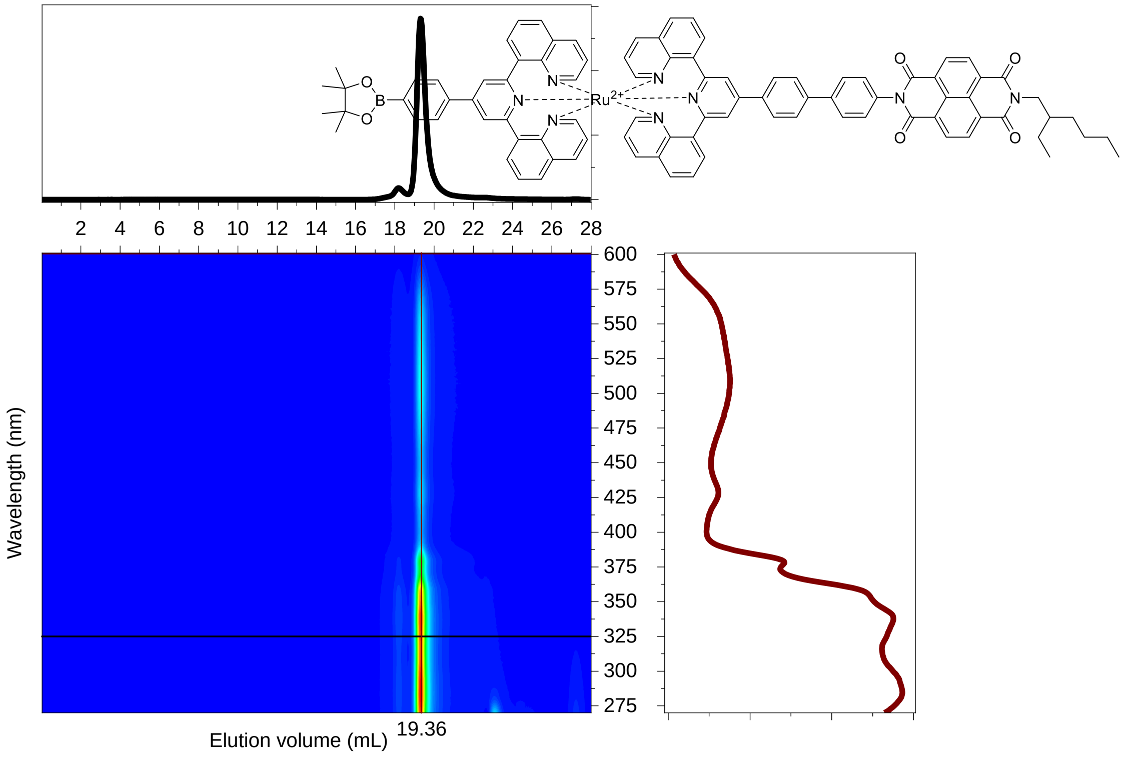

Figure S42. SEC elugram of [(TARA-Ph-dqp)Ru(dqp-Ph-Ph-NDI)]$^{2+}$ (DMAc + 0.08 wt% NH_4PF_6). Note the second species around 18.2 mL, assigned to the dimeric coupling product, deriving from the conversion of bromide into boronic acid ester.

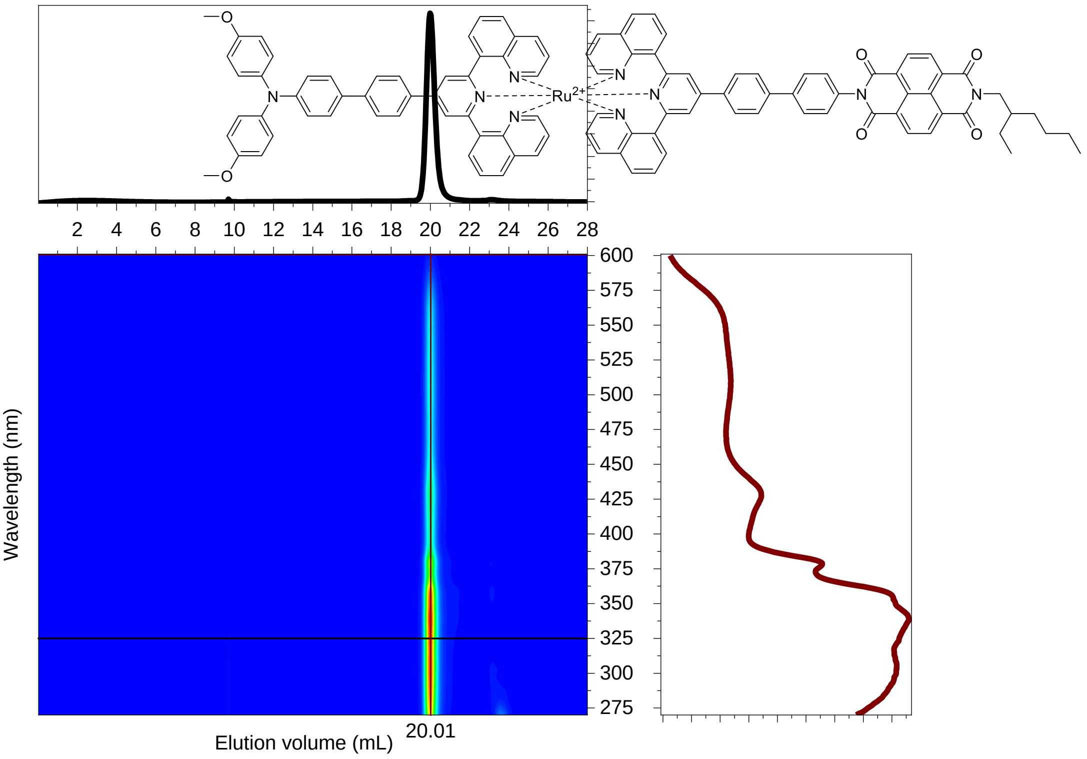

Figure S43. SEC elugram of $[(TARA\text{-}Ph\text{-}dqp)Ru(dqp\text{-}Ph\text{-}Ph\text{-}NDI)]^{2+}$ (DMAc + 0.08 wt% NH_4PF_6).

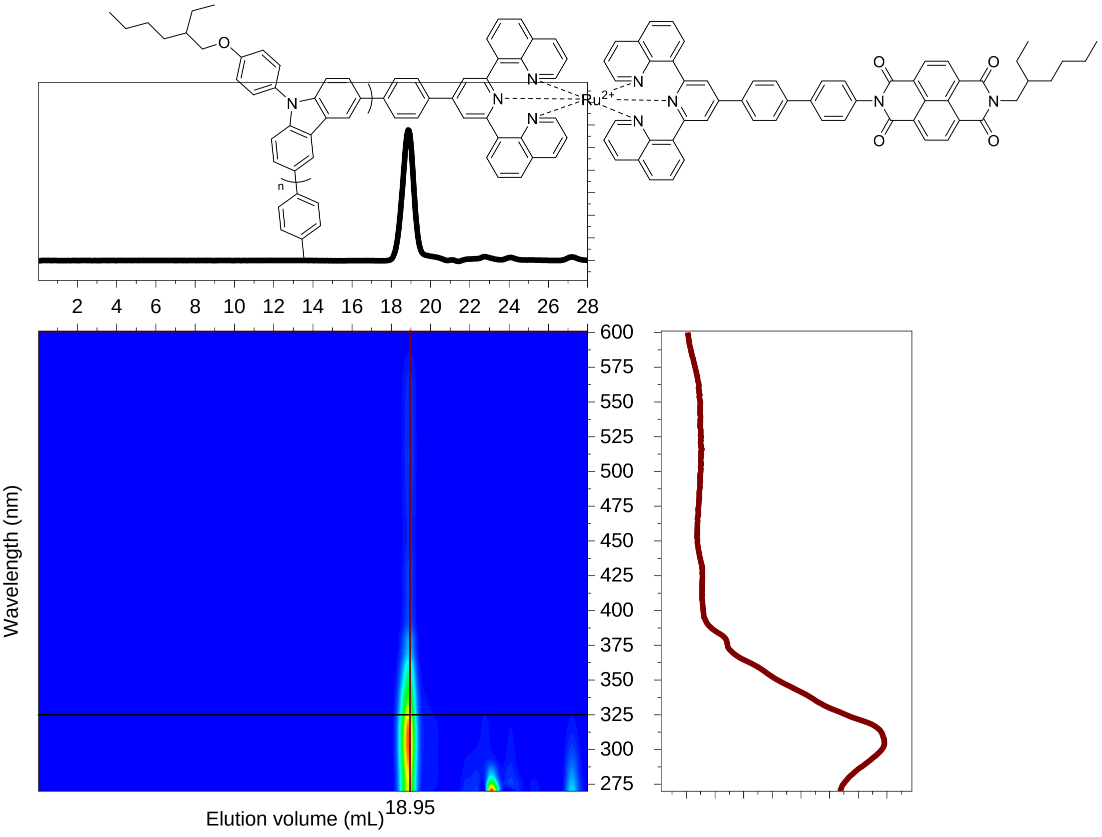

Figure S44. SEC elugram of $[(Tol\text{-}Carb_n\text{-}Ph\text{-}dqp)Ru(dqp\text{-}Ph\text{-}Ph\text{-}NDI)]^{2+}$ (DMAc + 0.08 wt% NH_4PF_6).

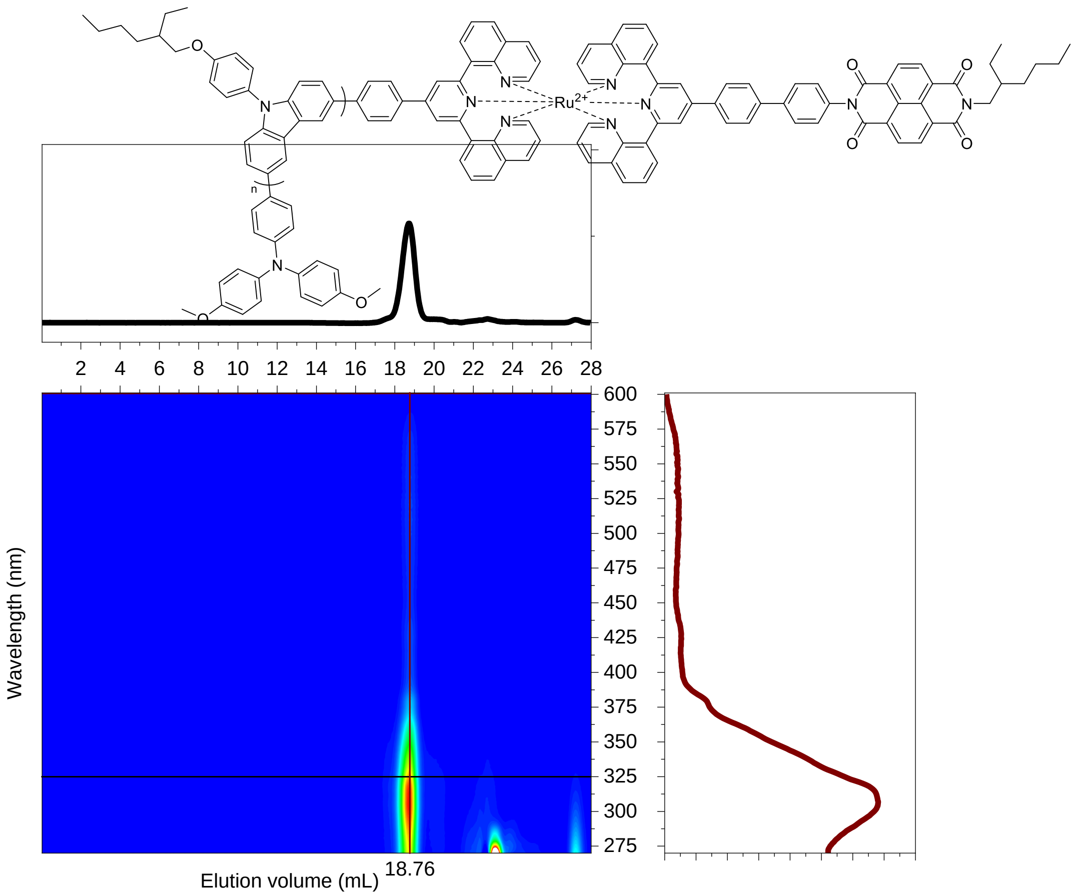

Figure S45. SEC elugram of [(TARA-Carb$_n$-Ph-dqp)Ru(dqp-Ph-Ph-NDI)]$^{2+}$ (DMAc + 0.08 wt% NH_4PF_6).

Table S1. SEC data for dyads and triads (DMAc + 0.08 wt% NH_4PF_6).[a]

Entry	Substance	V_p at 325 nm (mL)	V_p at 500 nm (mL)
**A**	$[Ru(dqp\text{-}Ph\text{-}Br)_2]2+$	20.30	20.30
**B**	$[(TARA\text{-}Ph\text{-}dqp)Ru(dqp\text{-}Ph\text{-}Br)]^{2+}$	20.26	20.26
**C[b]**	$[(Br\text{-}Ph\text{-}dqp)Ru(dqp\text{-}Ph\text{-}Ph\text{-}NDI)]^{2+}$	19.54	19.54
**D**	$[(TARA\text{-}Ph\text{-}dqp)Ru(dqp\text{-}Ph\text{-}Ph\text{-}NDI)]^{2+}$	19.98	19.98
**E**	$[(Tol\text{-}Carb_n\text{-}Ph\text{-}dqp)Ru(dqp\text{-}Ph\text{-}Ph\text{-}NDI)]^{2+}$	18.94[a]	18.86[a]
**F**	$[(TARA\text{-}Carb_n\text{-}Ph\text{-}dqp)Ru(dqp\text{-}Ph\text{-}Ph\text{-}NDI)]^{2+}$	18.74[a]	18.78[a]

[a] Extracted traces from 2D SEC elugrams. The apparent shift is due to longer chain lengths at higher elution volumes, *i.e.*, the normalized intensity at 500 nm correlates to the one [Ru] unit while the normalized intensity at 325 nm correlates to the distribution of the Carb-units (*vide infra*). [b] Br-Ph/Ph-Ph-NDI characterization reproduced for clarity.[8]

Scheme S1. Contributions to absorption intensities for polymer chains of different lengths.[a]

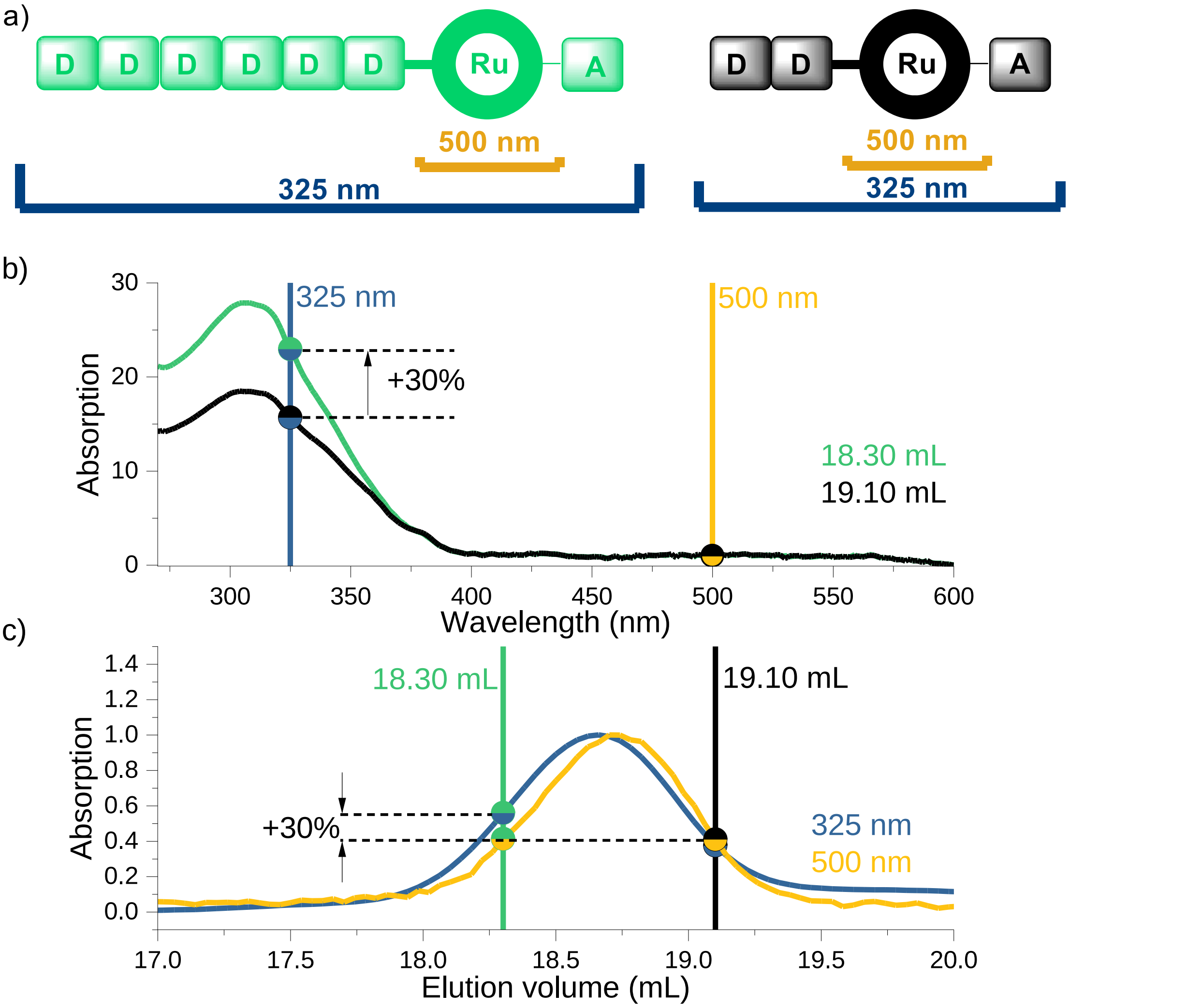

[a](a) Schematic representation of triads equipped with long (green) or short (black) donor polymer chains and marked regions which contribute to their absorption at 325 nm (blue) or 500 nm (yellow). (b) Normalized (on absorption at 500 nm) absorption spectra of triads equipped with long (green) or short (black) donor polymer chains as extracted from smaller (18.3 mL) or larger (19.1 mL) elution volumes in size exclusion chromatography data. Note the highlighted absorption values at 325 nm (blue) and 500 nm (yellow) and the 30% increase indicated by dashed lines at 325 nm while the absorption at 500 nm is equal. (c) Normalized size-exclusion chromatograms at 325 nm (blue) or 500 nm (yellow) detection. Note the highlighted elution volumes at 18.3 mL (green) and 19.1 mL (black) and the 30% increase indicated by dashed lines at 325 nm traces for isoabsorbing points in the 500 nm trace.

While monodisperse (molecular) dyads and triads show perfectly overlaps of both 325 nm and 500 nm traces in size-exclusion chromatography, both polymeric triad and tetrad show a *shift* towards shorter elution volumes. At shorter elution volumes, longer polymer chains are found compared to shorter ones, which are found at higher elution volumes. Comparing to triads with a short (black) and a long polymer chain (green, Scheme S1a), one can, according to the absorption spectra of respective compounds, indicate areas in one molecule which contribute to the absorption at either 325 nm (marked with blue bracket) or 500 nm (marked with yellow bracket). For longer polymer chains, the contribution to the absorption at 325 nm is larger as more repeating units contribute to the absorption in the UV/vis region. The characteristic absorption at 500 nm remains the same for both molecules, as only one ruthenium center contributes at this wavelength in both molecules. These statements are underlined by absorption spectra (Scheme S1b) of triads with longer polymeric chains (green, extracted at smaller elution volumes) and ones with shorter polymeric chains (black, extracted at larger elution volumes). In the normalized spectra, the longer polymer chains show an absorption at 325 nm (blue) increased by 30% (dashed lines) while the absorption at 500 nm is normalized on 1 in both cases. Likewise, the same increasing factor is found when comparing size-exclusion traces with detection at 325 nm and 500 nm (Scheme S1c). At elution volumes with identical absorption values at 500 nm (yellow dots at 18.3 and 19.1 mL), the traces recorded at 325 nm reveal an increased valued from short to long polymer chains of 30% while the absorption value at 500 nm stays identical. Thus, the difference in the two traces (UV region at 325 nm and vis region at 500 nm) can be explained by chain length variations and does not hint toward contaminations of the samples.

5 Electrochemistry

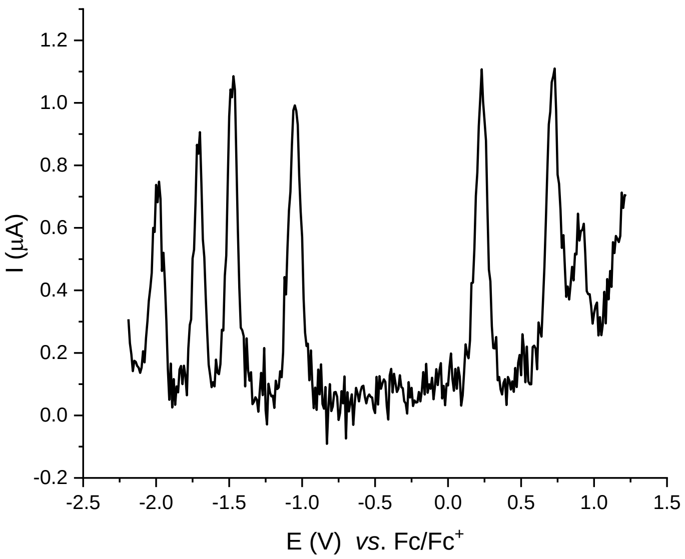

Figure S 46. DPV of [(TARA-Ph-dqp)Ru(dqp-Ph-Ph-NDI)]$^{2+}$ (CH_2Cl_2, 0.1 M $TBAPF_6$).

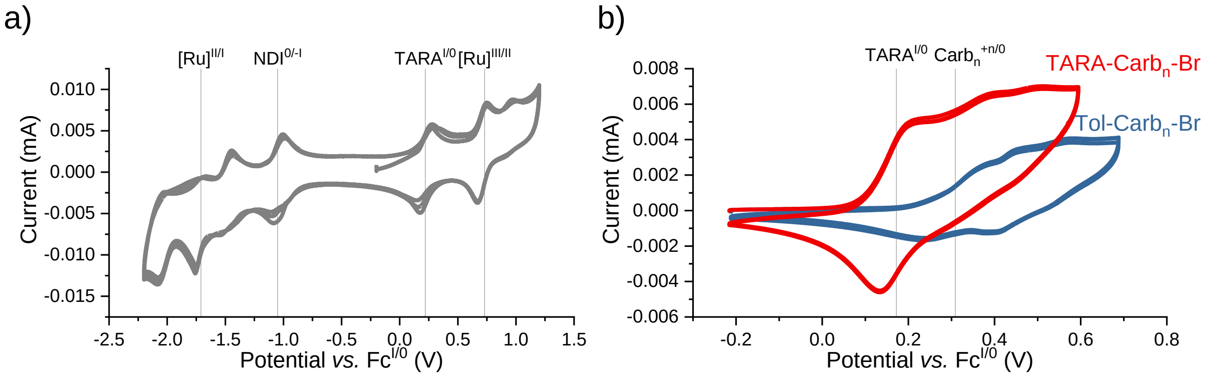

Figure S47. Cyclic voltammograms (CH_2Cl_2, 0.1 M $TBAPF_6$, *vs*. $Fc^{+/0}$) of (a) the molecular triad [TARA-Ph-dqp)Ru(dqp-Ph-Ph-NDI)]$^{2+}$ at 100 mV/s, and (b) the donor polymers Tol-$Carb_n$-Br (blue) and TARA-$Carb_n$-Br (red) at 50 mV/s. Vertical lines indicate half-wave potentials of the redox-active units. Note the subtle difference of the TARA oxidation if attached to the electron-withdrawing $[Ru]^{II}$-phenylene at +0.230 V (a) or the electron-releasing poly(carbazole) at +0.172 V (b).

6 Spectroelectrochemistry

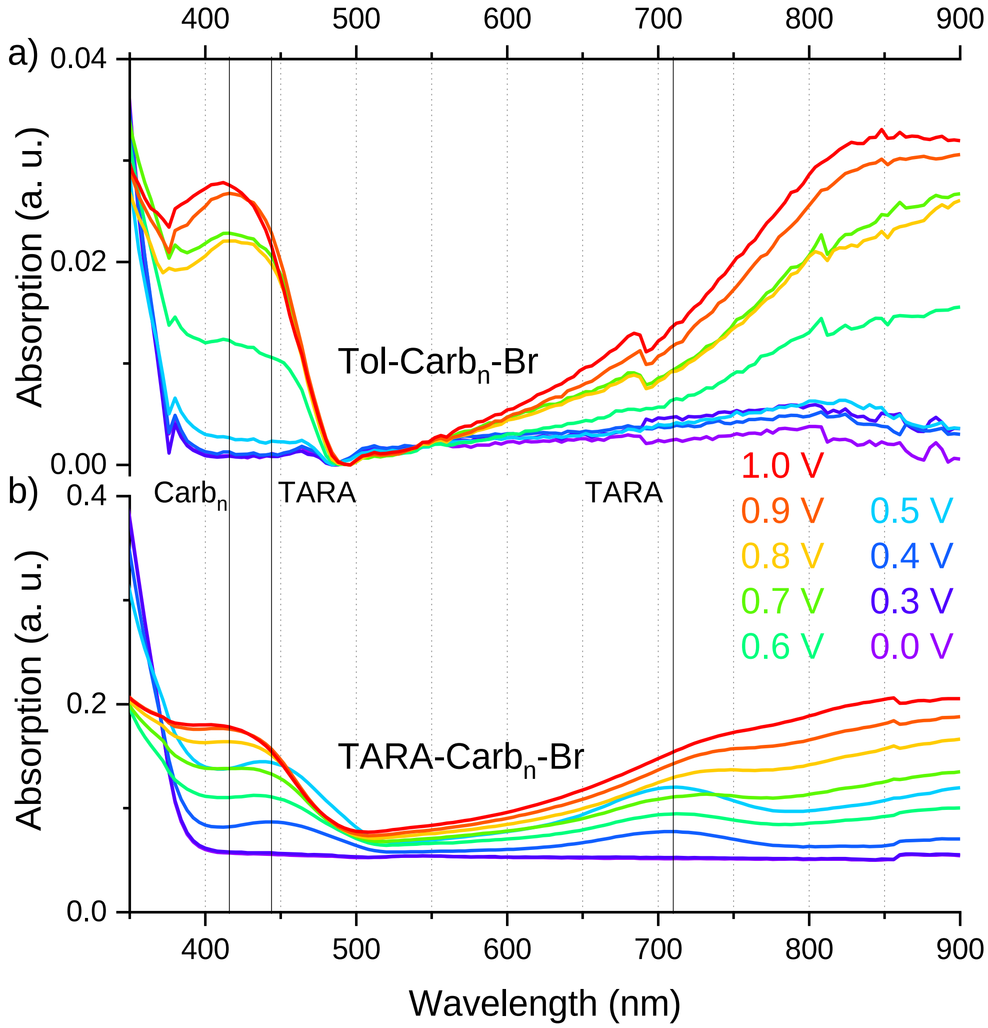

Figure S48. Spectroelectro-chemical analysis (CH_2Cl_2, 0.1 M $TBAPF_6$, *vs*. $Ag^{+/0}$) of the donor polymers (a) Tol-$Carb_n$-Br and (b) TARA-$Carb_n$-Br. Note the onset of characteristic spectral features of $Carb_n$ (> +0.5 V, 414 nm) and TARA (> +0.3 V, 445 and 710 nm).

7 Transient absorption and emission spectroscopy

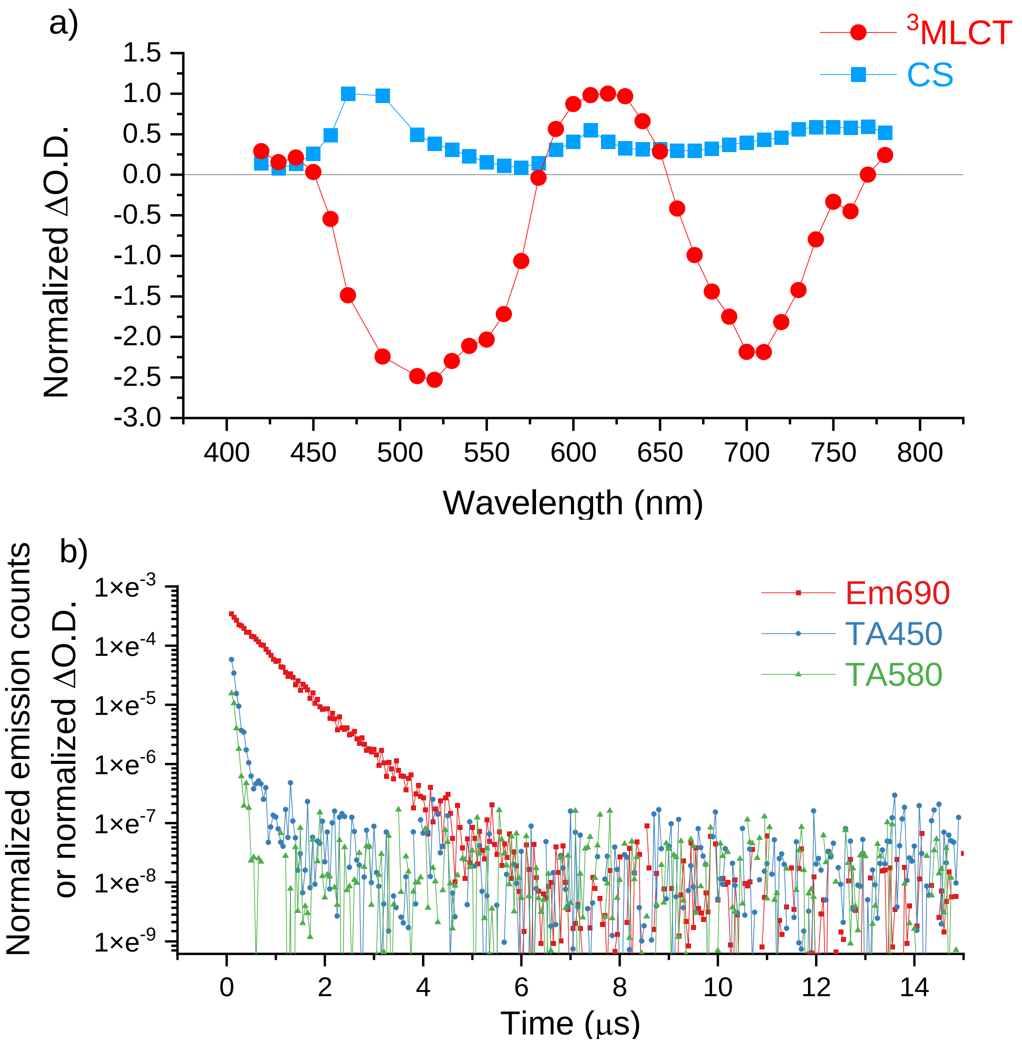

Figure S49. Molecular triad [(TARA-Ph-dqp)Ru(dqp-Ph-Ph-NDI)]$^{2+}$: (a) Normalized DAS from biexponential fit with characteristic ^{3}MLCT (black curve) and charge-separated state (blue). (b) Decay profile at isosbestic points of ^{3}MLCT to illustrate mono-exponential decay (logarithmic scale). Data from freeze-pump-thawed sample.

7.1 Reference dyads

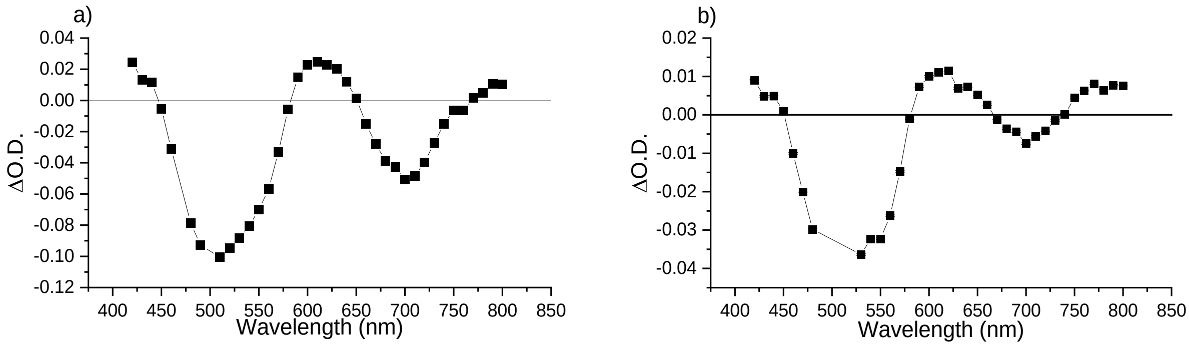

Figure S50. Decay associated spectra of (a) [(Br-Ph-dqp)Ru(dqp-Ph-Ph-NDI)]$^{2+}$ and (b) [(TARA-Ph-dqp)Ru(dqp-Ph-Br)]$^{2+}$ (dry CH_2Cl_2). Note the ground state bleach with isosbestic points at 450 nm and 580 nm, respectively. Stimulated emission causes negative signals centered at 700 nm. The resulting isosbestic points shift according to the quantum yield, for Br-Ph/Ph-Ph-NDI (650 nm and 770 nm) and for TARA-Ph/Ph-Br (665 nm and 740 nm).

7.2 Molecular triad (III$_a$)

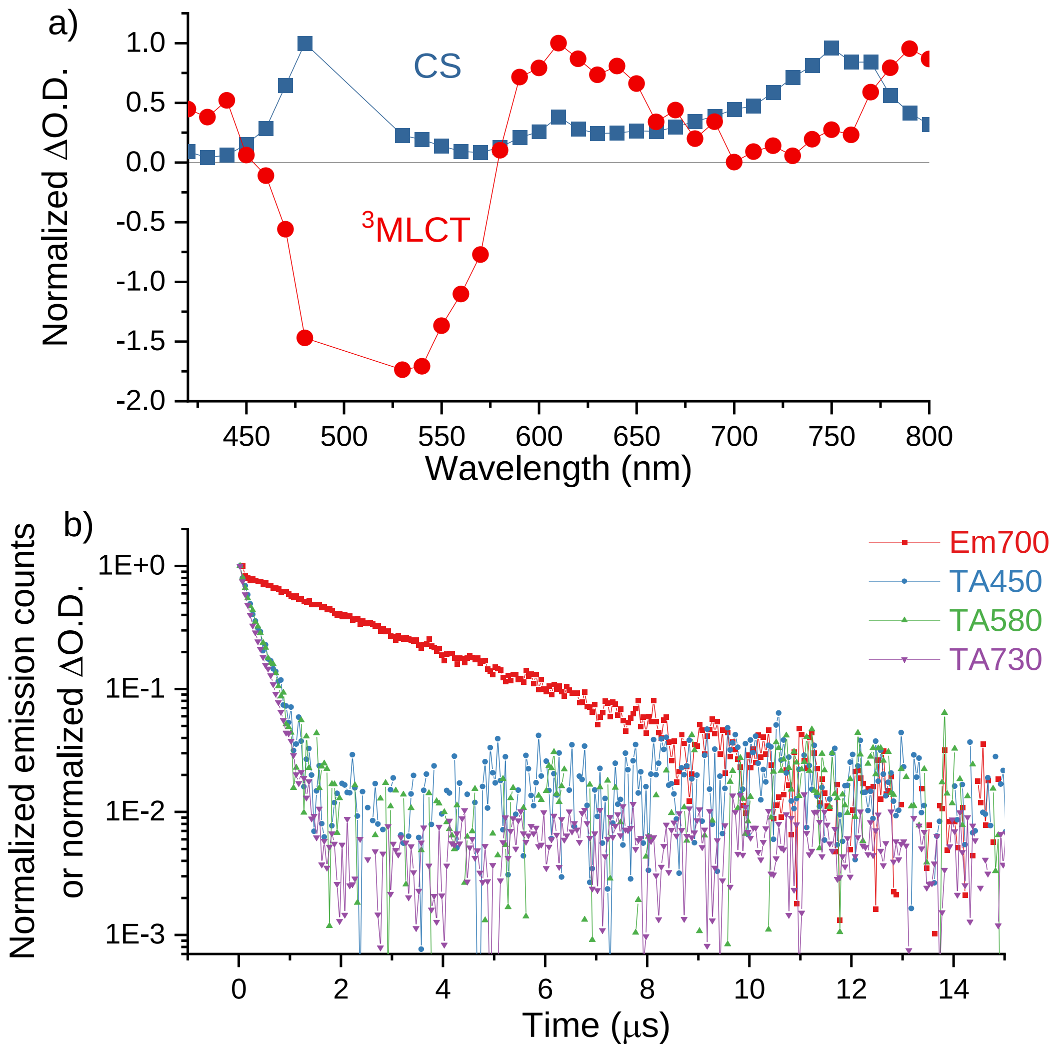

Figure S51. Data for molecular TARA-Ph/Ph-Ph-NDI triad: (a) Normalized decay-associated spectra from biexponential fit with characteristic ^{3}MLCT (red) and residual charge-separated state (blue). (b) Normalized TA decay profile at isosbestic points of ^{3}MLCT and residual emission decay (red) to illustrate mono-exponential decay (logarithmic scale). Data from glovebox-sample.

7.3 Polymer-based triad (III_b)

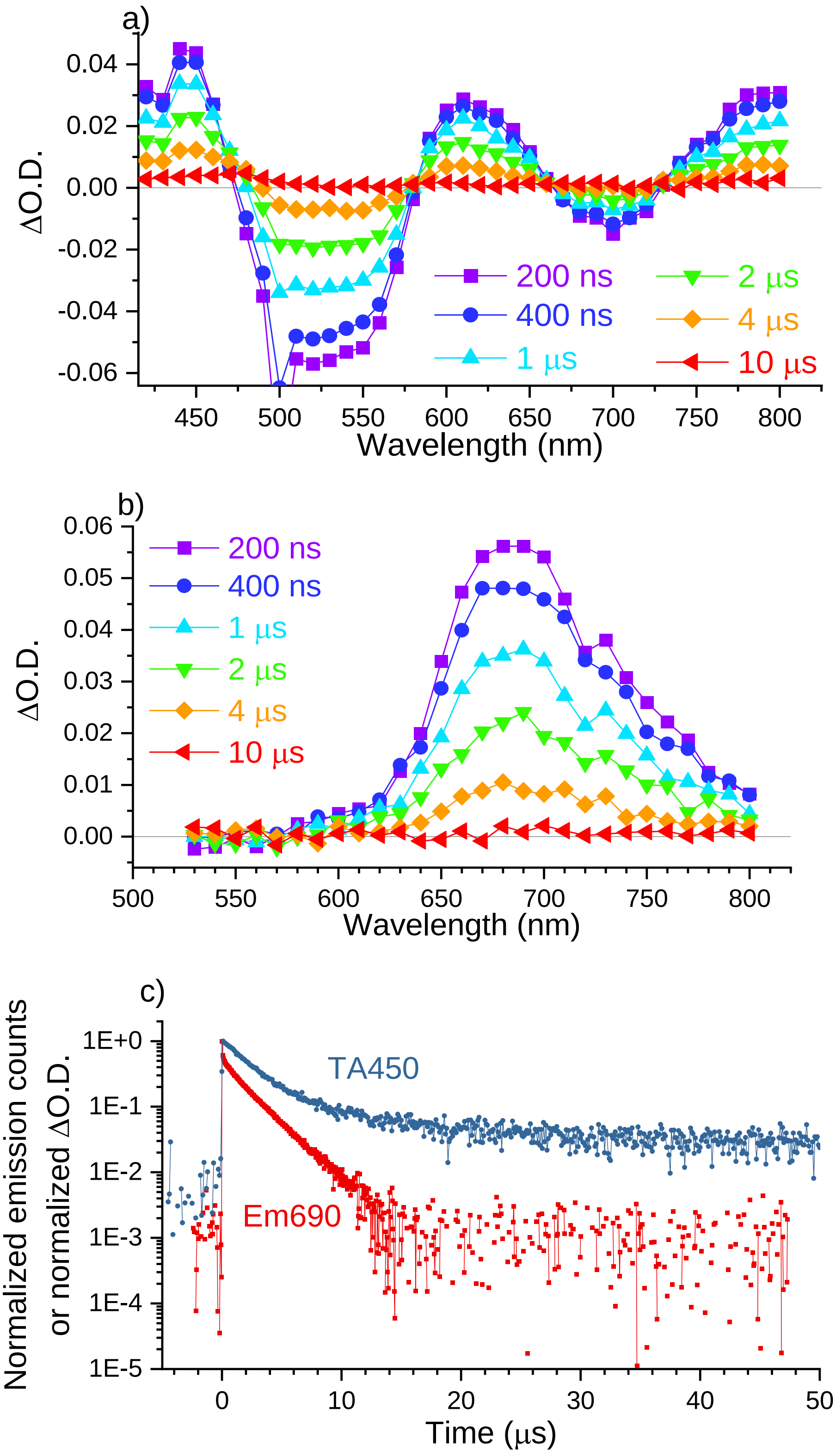

Figure S52. (a) Transient absorption spectra of Tol-Carb$_n$-Ph/Ph-Ph-NDI at selected delay times in CH_2Cl_2. (b) Respective emission spectra at selected delay times in CH_2Cl_2. (c) Decay profile to illustrate residual mono-exponential emission decay (red) and non-mono-exponential decay at selected wavelength (logarithmic scale). Data from freeze-pump-thawed sample.

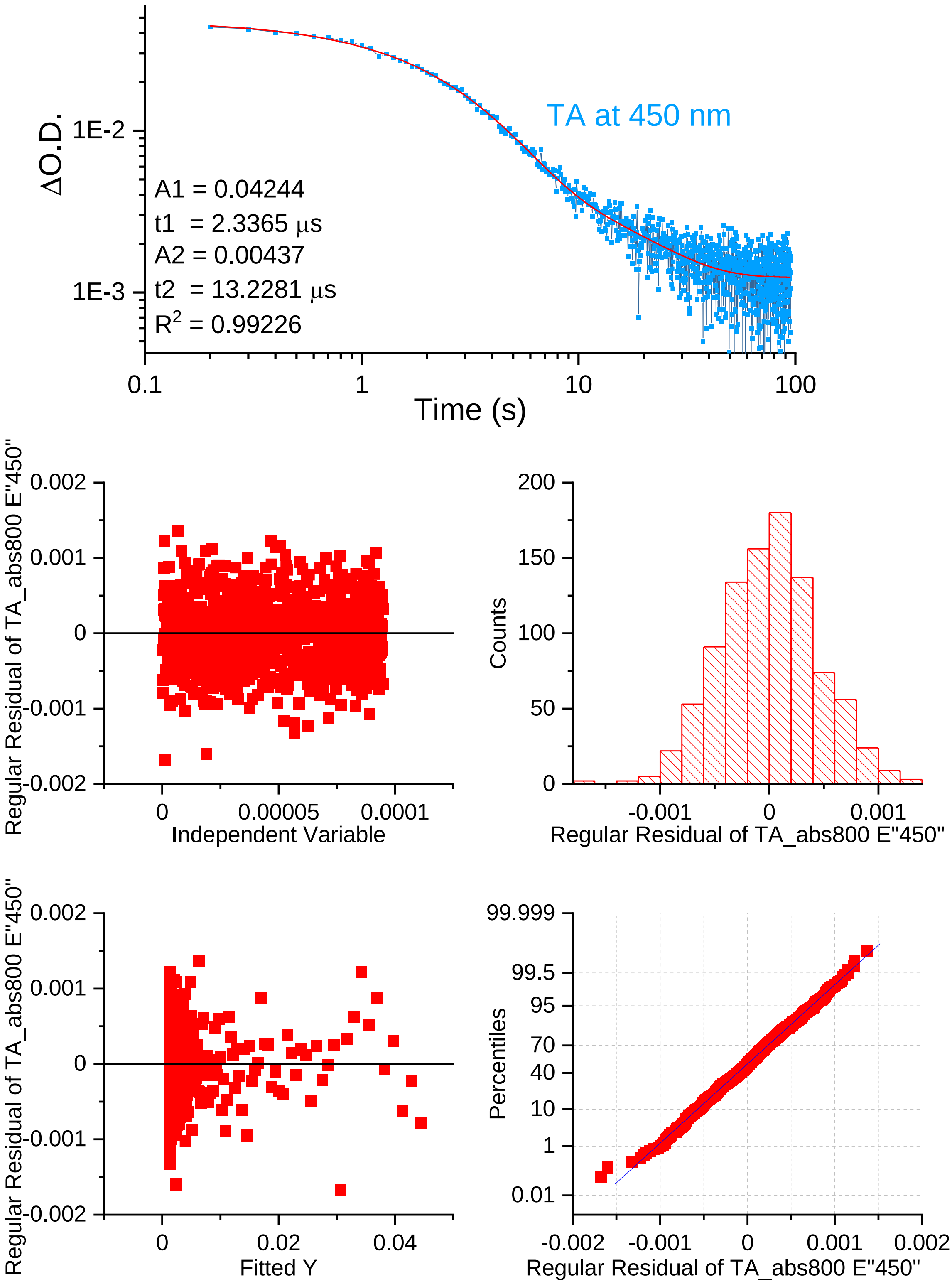

Figure S53. Bi-exponential fit of CS state decay of Tol-Carb$_n$-Ph/Ph-Ph-NDI at 450 nm (isosbestic with respect to the ^{3}MLCT decay) with 2.3 µs (91%) and 13.2 µs (9%).

7.4 Polymer-based tetrad (III$_c$)

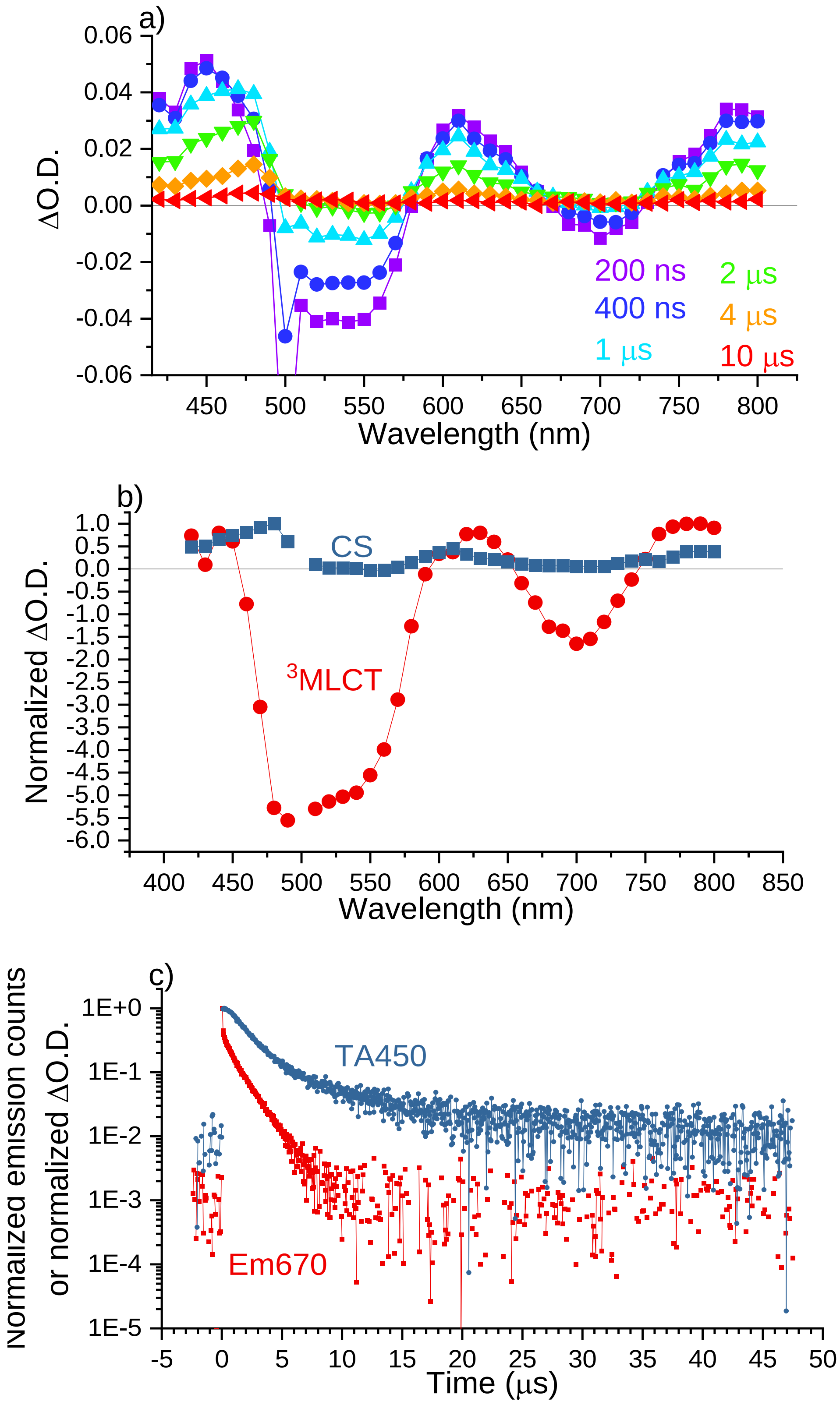

Figure S54. (a) Transient absorption spectra of TARA-Carb$_n$-Ph/Ph-Ph-NDI at selected delay times in CH_2Cl_2.(b) Extracted, normalized decay associated spectra from biexponential fit with characteristic ^{3}MLCT (red) and charge-separated state (blue). (c) Decay profile to illustrate mono-exponential emission decay (red) and non-mono-exponential decay at selected wavelengths (logarithmic scale). Data from freeze-pump-thawed sample.

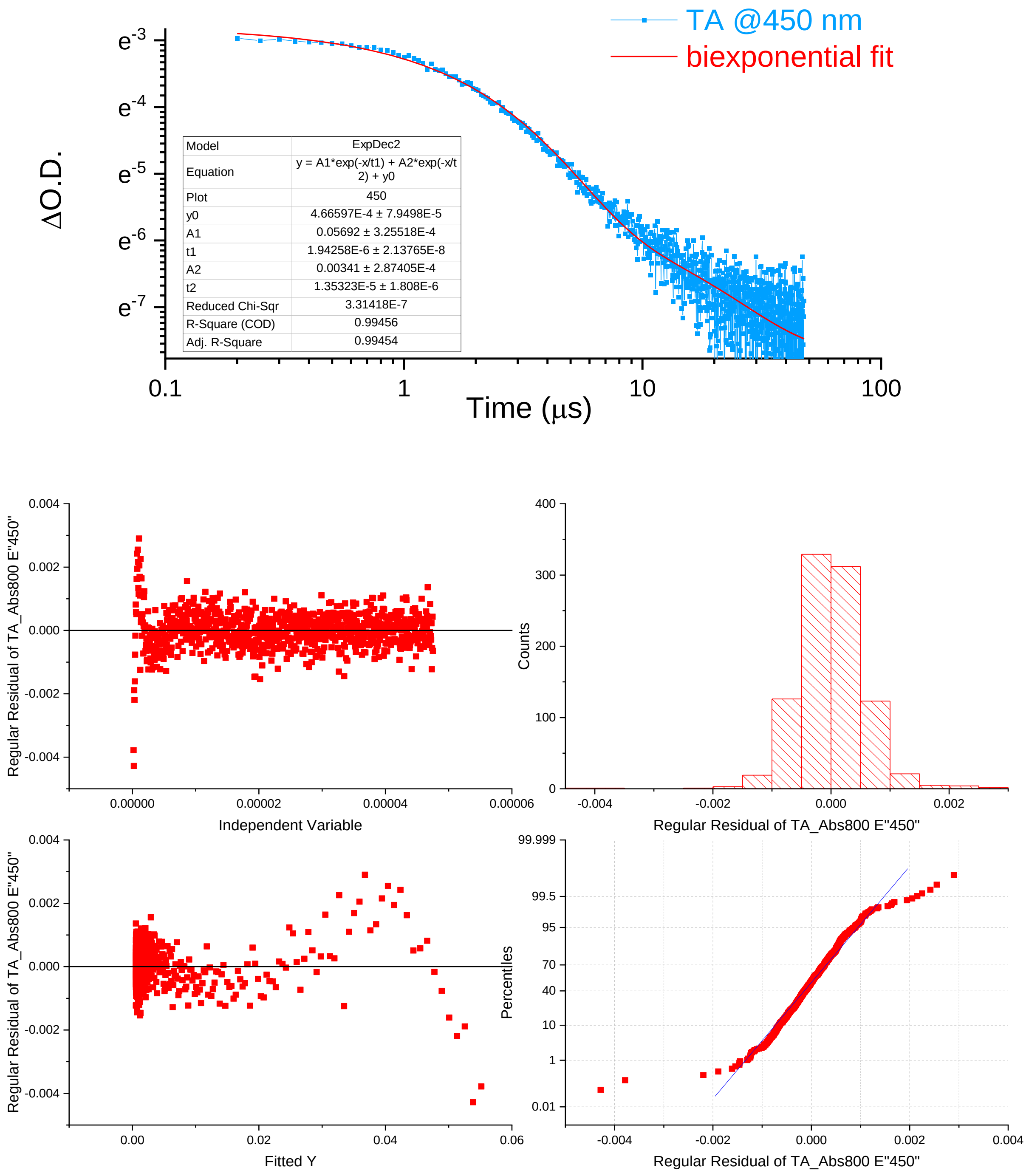

Figure S55: Bi-exponential fit of CS state decay of TARA-Carb$_n$-Ph/Ph-Ph-NDI at 450 nm (isosbestic with respect the ^{3}MLCT decay) with 1.9 µs (94%) and 13.5 µs (6%). Note the slight deviation at short timescale below 1 µs.

8 DFT calculations

The driving forces for oxidative or reductive excited state quenching on the basis of DFT data were estimated as follows: The frontier molecular orbitals (MOs) of the optimized singlet state (S_0) were identified as exemplified for the HOMO and LUMO depicted in Figure S56 to Figure S58. The orbitals were names according to their localization, i.e., “Ru”, “TARA”, “NDI”, “PTZ”,“BQ” or “qu” including the nature of the redox process *vs*. ferrocene (“ox” denotes oxidation, “red” denotes reduction”). In case of the NDI units, the alignment was specified. Table S2 summarizes the MO energies, the available electrochemical data (see manuscript for details) and the quadratic polynomial fit for their mutual correlation including the deviation between experimental and projected values. Figure S59 visualizes the good agreement and validity of the approach to estimate redox potentials by MO energies (note the error cancellation within the series). The driving forces were calculated according to equations (1) and (2) in the manuscript to corroborate the order of the oxidation/reduction processes. The driving force for oxidative quenching for triad $\mathbf{I_a}$ is found larger than for triad $\mathbf{III_a}$, the effect of π-stacking is comparable for an ideal NDI-NDI dimer (see $\mathbf{II_{b4}}$) or NDI-qu (see $\mathbf{II_{b1}}$ and $\mathbf{II_{b4}}$) and leads to a stabilization of as much as –0.2 eV *vs*. a free NDI unit (see $\mathbf{III_a}$ and $\mathbf{II_{b4}}$).

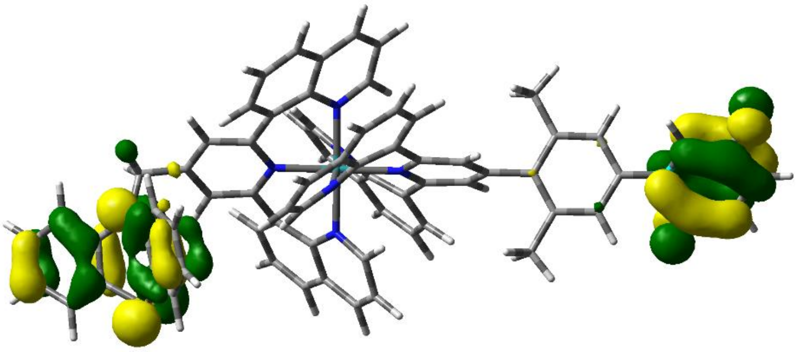

Figure S56. HOMO and LUMO of the triad PTZ-CH_2/Xyl-BQ (I_a) drawn at isovalue 0.04 to illustrate the quasi-axial CS state.

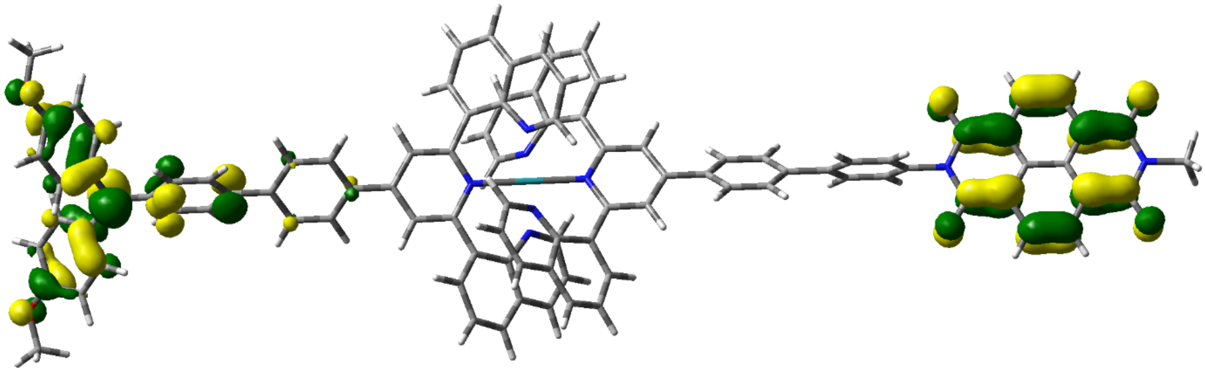

Figure S57. HOMO and LUMO of the triad TARA-Ph/Ph-Ph-NDI (III_a) drawn at isovalue 0.04 to illustrate the axial CS state.

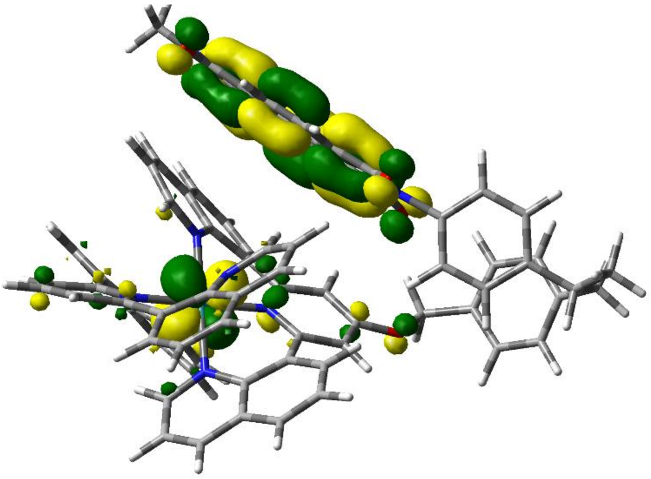

Figure S58. HOMO and LUMO of the dyad II_{b1} drawn at isovalue 0.04 to illustrate the π-stacking of the Ru complex and the NDI unit.

Table S2. Computational and experimental data for redox-active dyads and triads.[a]

Name	Assignment	MO energies (Hartree)	Experi-mental redox potentials (V)	Calcula-ted redox poten-tials (V)	error[b] (V)	ΔG_{ox} (eV)
TARA-Ph/ Ph-Ph-NDI (III_a)	Ru(ox)	–0.20858	0.73[c]	0.66	–0.07	n.a.
	TARA(ox)	–0.17902	0.23[c]	0.18	–0.05	n.a.
	NDI(red)	–0.12360	-1.05[c]	–1.01	0.04	–0.11
	qu(red)	–0.09735	-1.71[c]	–1.69	0.02	n.a.
PTZ-CH_2/ Xyl-BQ (I_a)	Ru(ox)	–0.21169	0.71[d]	0.71	–0.00	n.a.
	PTZ(ox)	–0.20080	0.40[d]	0.55	0.15	n.a.
	BQ(red)	–0.12959	-0.80[d]	–0.86	–0.06	–0.21
	qu(red)	–0.09642	-1.70[d]	–1.72	–0.02	n.a.
II_{b4}	Ru(ox)	–0.20901	n.a.	0.67	n.a.	n.a.
	NDI_{dimer}(red)[e]	–0.13123	n.a.	–0.82	n.a.	–0.29
	$NDI_{stacked}$(red)[e]	–0.13084	n.a.	–0.83	n.a.	–0.28
	$NDI_{peripheral}$(red)[e]	–0.12352	n.a.	–1.01	n.a.	–0.10
	$NDI_{stacked}$(red)[e]	–0.12220	n.a.	–1.04	n.a.	–0.07
	qu(red)	–0.09694	n.a.	–1.70	n.a.	n.a.
II_{b1}	Ru(ox)	–0.20970	n.a.	0.68	n.a.	n.a.
	$NDI_{stacked}$(red)	–0.13093	n.a.	-0.83	n.a.	–0.27
	Qu(red)	–0.09689	n.a.	–1.70	n.a.	n.a.

a. Redox potentials *vs.* $Fc^{+/0}$ b. Difference between the experimental and the calculated potentials. c. in CH_2Cl_2 containing 0.1 M $TBAPF_6$. d. in CH_3CN containing 0.1 M $TBAPF_6$. e. See manuscript for details. n.a. denotes not applicable or not available.

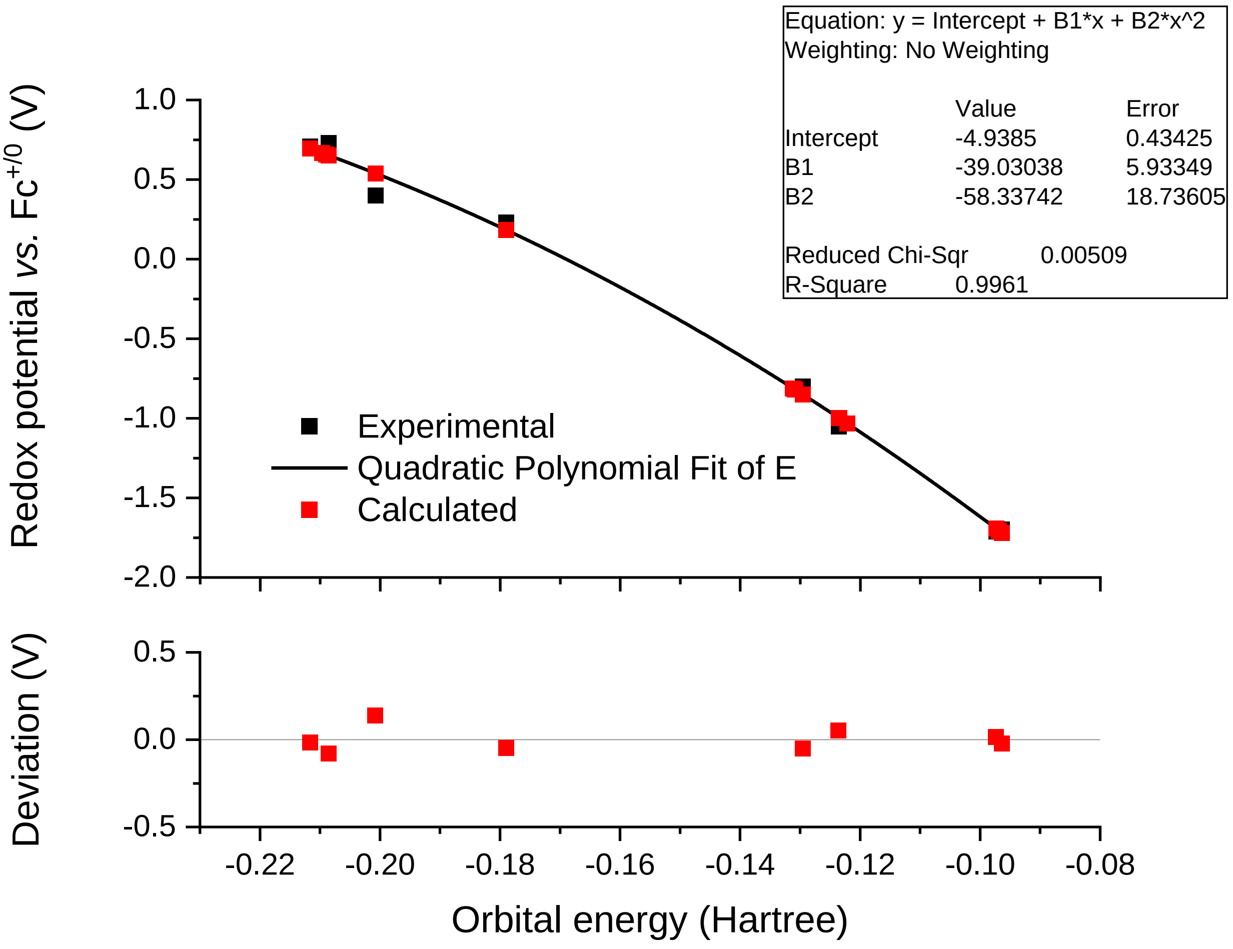

Figure S59. (Top) Calculated redox potentials based on MO energies (red) *vs*. experimental values and quadratic polynomial fit (black). (Bottom) Deviation between experimental and calculated redox potential. See also Table S2.
